# Resequencing of a Pekin duck breeding population provides insights into the genomic response to short-term artificial selection

**DOI:** 10.1093/gigascience/giad016

**Published:** 2023-03-27

**Authors:** Simeng Yu, Zihua Liu, Ming Li, Dongke Zhou, Ping Hua, Hong Cheng, Wenlei Fan, Yaxi Xu, Dapeng Liu, Suyun Liang, Yunsheng Zhang, Ming Xie, Jing Tang, Yu Jiang, Shuisheng Hou, Zhengkui Zhou

**Affiliations:** State Key Laboratory of Animal Nutrition; Key Laboratory of Animal (Poultry) Genetics Breeding and Reproduction, Ministry of Agriculture and Rural Affairs; Institute of Animal Science, Chinese Academy of Agricultural Sciences, Beijing 100193, China; Key Laboratory of Animal Genetics, Breeding and Reproduction of Shaanxi Province, College of Animal Science and Technology, Northwest A&F University, Yangling 712100, China; Key Laboratory of Animal Genetics, Breeding and Reproduction of Shaanxi Province, College of Animal Science and Technology, Northwest A&F University, Yangling 712100, China; Key Laboratory of Animal Genetics, Breeding and Reproduction of Shaanxi Province, College of Animal Science and Technology, Northwest A&F University, Yangling 712100, China; Key Laboratory of Animal Genetics, Breeding and Reproduction of Shaanxi Province, College of Animal Science and Technology, Northwest A&F University, Yangling 712100, China; Key Laboratory of Animal Genetics, Breeding and Reproduction of Shaanxi Province, College of Animal Science and Technology, Northwest A&F University, Yangling 712100, China; State Key Laboratory of Animal Nutrition; Key Laboratory of Animal (Poultry) Genetics Breeding and Reproduction, Ministry of Agriculture and Rural Affairs; Institute of Animal Science, Chinese Academy of Agricultural Sciences, Beijing 100193, China; State Key Laboratory of Animal Nutrition; Key Laboratory of Animal (Poultry) Genetics Breeding and Reproduction, Ministry of Agriculture and Rural Affairs; Institute of Animal Science, Chinese Academy of Agricultural Sciences, Beijing 100193, China; State Key Laboratory of Animal Nutrition; Key Laboratory of Animal (Poultry) Genetics Breeding and Reproduction, Ministry of Agriculture and Rural Affairs; Institute of Animal Science, Chinese Academy of Agricultural Sciences, Beijing 100193, China; State Key Laboratory of Animal Nutrition; Key Laboratory of Animal (Poultry) Genetics Breeding and Reproduction, Ministry of Agriculture and Rural Affairs; Institute of Animal Science, Chinese Academy of Agricultural Sciences, Beijing 100193, China; State Key Laboratory of Animal Nutrition; Key Laboratory of Animal (Poultry) Genetics Breeding and Reproduction, Ministry of Agriculture and Rural Affairs; Institute of Animal Science, Chinese Academy of Agricultural Sciences, Beijing 100193, China; State Key Laboratory of Animal Nutrition; Key Laboratory of Animal (Poultry) Genetics Breeding and Reproduction, Ministry of Agriculture and Rural Affairs; Institute of Animal Science, Chinese Academy of Agricultural Sciences, Beijing 100193, China; State Key Laboratory of Animal Nutrition; Key Laboratory of Animal (Poultry) Genetics Breeding and Reproduction, Ministry of Agriculture and Rural Affairs; Institute of Animal Science, Chinese Academy of Agricultural Sciences, Beijing 100193, China; Key Laboratory of Animal Genetics, Breeding and Reproduction of Shaanxi Province, College of Animal Science and Technology, Northwest A&F University, Yangling 712100, China; State Key Laboratory of Animal Nutrition; Key Laboratory of Animal (Poultry) Genetics Breeding and Reproduction, Ministry of Agriculture and Rural Affairs; Institute of Animal Science, Chinese Academy of Agricultural Sciences, Beijing 100193, China; State Key Laboratory of Animal Nutrition; Key Laboratory of Animal (Poultry) Genetics Breeding and Reproduction, Ministry of Agriculture and Rural Affairs; Institute of Animal Science, Chinese Academy of Agricultural Sciences, Beijing 100193, China

**Keywords:** duck, genome, artificial selection, breast muscle weight, selection signatures

## Abstract

**Background:**

Short-term, intense artificial selection drives fast phenotypic changes in domestic animals and leaves imprints on their genomes. However, the genetic basis of this selection response is poorly understood. To better address this, we employed the Pekin duck Z2 pure line, in which the breast muscle weight was increased nearly 3-fold after 10 generations of breeding. We *denovo* assembled a high-quality reference genome of a female Pekin duck of this line (GCA_003850225.1) and identified 8.60 million genetic variants in 119 individuals among 10 generations of the breeding population.

**Results:**

We identified 53 selected regions between the first and tenth generations, and 93.8% of the identified variations were enriched in regulatory and noncoding regions. Integrating the selection signatures and genome-wide association approach, we found that 2 regions covering 0.36 Mb containing *UTP25* and *FBRSL1* were most likely to contribute to breast muscle weight improvement. The major allele frequencies of these 2 loci increased gradually with each generation following the same trend. Additionally, we found that a copy number variation region containing the entire *EXOC4* gene could explain 1.9% of the variance in breast muscle weight, indicating that the nervous system may play a role in economic trait improvement.

**Conclusions:**

Our study not only provides insights into genomic dynamics under intense artificial selection but also provides resources for genomics-enabled improvements in duck breeding.

## Background

Artificial selection experiments can help to understand the mechanism that allows populations to adapt to strong selection pressure [1, 2]. In combination with population-level genome sequencing, attempts have been made to identify alleles whose frequencies change systematically during selection experiments [3–5]. However, most studies were focused on lower organisms and were retrospective or used a single time point to characterize the dynamic changes in allele frequencies [1, 6–8]. Selecting a suitable domestic animal model for continuous high-intensity artificial selection trials may improve our understanding of the genetic basis of complex traits in domestic animals.

Studies of 2 representative bidirectional selected resource populations in which chicken body weight [9–13] and abdominal fat [14–18] were selected have shown that continuous artificial selection results in obvious phenotypic and genomic differentiation in poultry. Artificial selection signals have also been identified in animals, such as rabbits [19], sheep [20], goats [21, 22], and pigs [23]. However, many domestic animal populations established for selection experiments are not as complete as chicken populations because of the long generation interval and sequencing costs in these species. Ducks (*Anas platyrhynchos*) (NCBI:txid8839) are among the most economically important waterfowl; they can provide meat, eggs, and down for humans and show important characteristics, such as a short generation interval, high reproductive ability, and a long but traceable history of artificial selection [24]. Moreover, ducks, like other birds, have smaller genomes than nonavian terrestrial vertebrates. These characteristics make the duck an effective model for studying genomic footprints of artificial selection.

**Figure 1:**
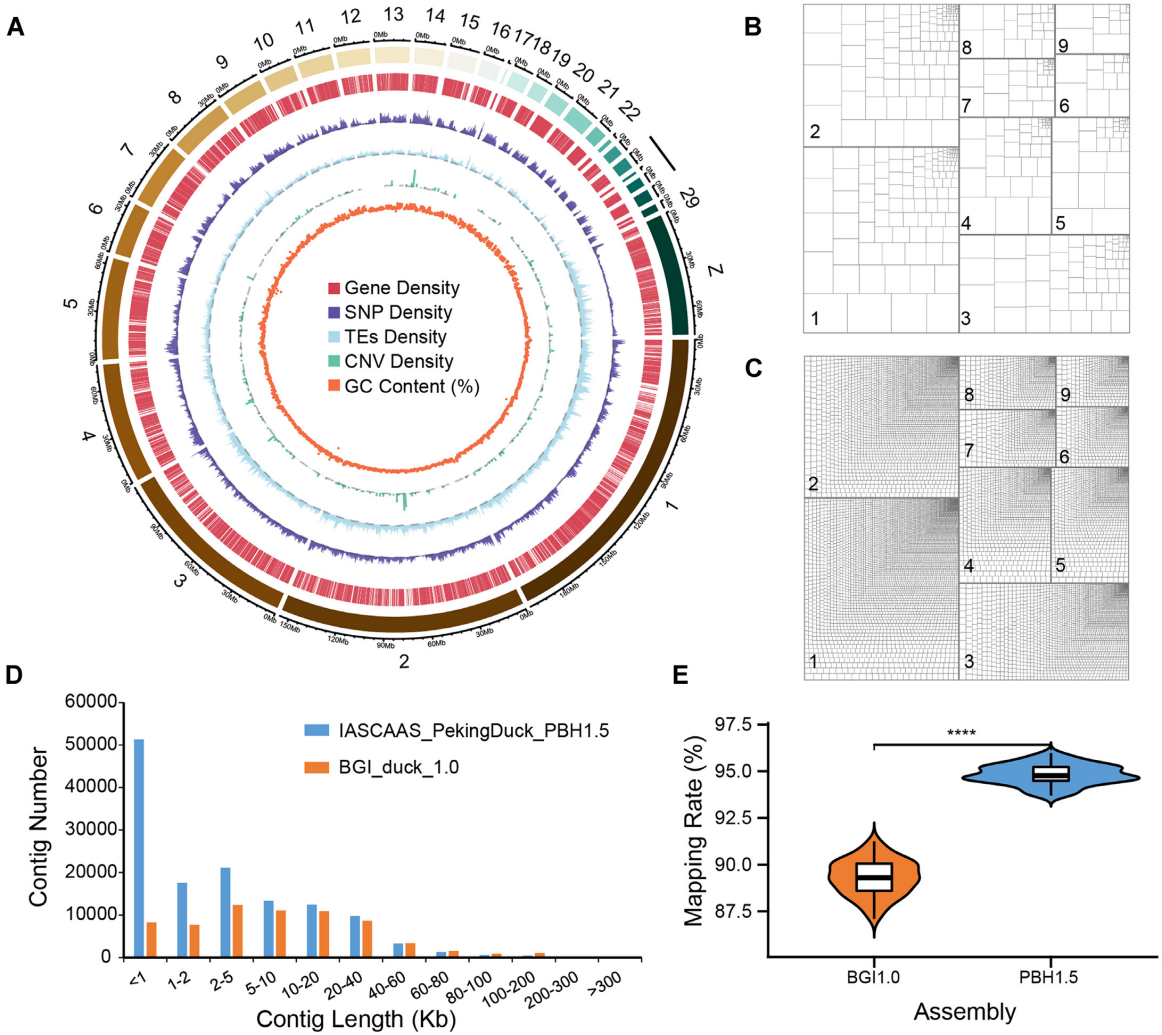
Overview of the assembly quality and characteristics of the Pekin duck genome. (A) Circular diagram depicting the characteristics of the GCA_003850225.1 assembly. The tracks from the outer to inner circles represent the following: chromosomes, gene density (window size of 200 kb), SNP density (window size of 200 kb), TE density (window size of 200 kb), CNV density (window size of 3 Mb), and GC content (%) (window size of 200 kb). (B, C) Tree maps of fragmentation differences between long-read (GCA_003850225.1) and short-read (BGI_duck1.0) Pekin duck genome assemblies. The size of each rectangle of each chromosome is scaled to that of the contig sequence. The larger and fewer the internal boxes are, the more contiguous the contigs. (D) Comparison of the sequence contig length distribution between long-read (GCA_003850225.1) and short-read (BGI_duck_1.0) Pekin duck genome assemblies. (E) Comparison of mapping rates when the Pekin duck population (119 birds from 10 generations) whole-genome resequencing data were mapped to GCA_003850225.1 and the short-read genome assembly (BGI_duck_1.0). A 2-tailed paired *t* test was used for statistical assessment. ****P < 0.0001.

High-quality reference genomes are the foundation for genetic research and molecular marker breeding, which can support innovations in sustainable animal production [25, 26]. To date, reference genomes have been published for a variety of domestic animals such as ducks, chickens, pigs, cattle, sheep, and goats [24, 27–31], providing important resources for livestock and poultry genetic breeding. To completely capture the variation present in the genome of our study population, we chose a female individual from our population for PacBio long-read sequencing and genome assembly to obtain a high-quality chromosome-level reference genome of Pekin duck (GCA_003850225.1) (Fig. 1A).

Herein, we utilized a Pekin duck pure line selected for breast muscle weight for 10 generations. The foundation stock was the local conserved population in Beijing, China. After 10 generations of high-intensity artificial selection, the breast muscle weight of the Pekin duck Z2 line increased from 80 g to 220 g. These data were investigated to elucidate the dynamic response patterns in the Pekin duck genome under artificial selection.

## Data Description

To understand the genetic basis of short-term, intense artificial selection, we utilized a Pekin duck pure line selected for breast muscle weight at 6 weeks of age. We implemented the whole-genome resequencing of 119 ducks across 10 generations (1 generation per year, 2005–2014) and mapped the resequencing data to the high-quality reference genomes assembled in this study. Using 8.60 million of single-nucleotide polymorphisms (SNPs), we tested for dynamic changes in population structure under short-term intense selection, and we assessed the genome for signatures of short-term intense selection associated with breast muscle weight.

## Analyses

### An improved Pekin duck genome assembly

To carry out the *de novo* assembly of the Pekin duck genome, we adopted a combination of PacBio long-read sequencing, BioNano optical mapping, and Hi-C technologies. We first generated 65.9 Gb of PacBio long reads with 50× genome coverage, 49 Gb of BioNano high-quality reads with 41× genome coverage, and 106 Gb of Hi-C reads with 82× genome coverage (Supplementary Table S1). Then, these data were used to assemble the new duck genome (Fig. 1A). Assembly was performed in a stepwise fashion (Supplementary Fig. S1), to generate assemblies with improvements for each process. First, we used the initial PacBio subreads to construct 2,682 contigs, yielding a contig N50 of 4.17 Mb (Table 1). Second, we used optical mapping (BioNano Genomics Irys) data to link the PacBio contigs into scaffolds, resulting in the population of 1,788 scaffolds, and the N50 was 6.24 Mb (Table 1). Then, the Hi-C data (Supplementary Table S2) were used to cluster the scaffolds at the chromosomal scale, resulting in 1,852 scaffolds (Supplementary Fig. S2). The final assembled genome length was 1.12 Gb, with scaffold N50 and contig N50 values of 76.13 Mb and 4.10 Mb, respectively (Table 1).

**Table 1:** Assembly statistics of the GCA_003850225.1 genome

Assembly	Number of scaffolds	Scaffold N50 (Mb)	Genome size
PacBio	─	─	1,154,570,803
PacBio + BioNano	1,788	6.24	1,153,069,495
PacBio + BioNano + Hi-C	1,852	41.14	1,134,893,859
GCA_003850225.1	1,330	76.13	1,134,894,103

We next evaluated the quality of the new assembly. BUSCO (RRID:SCR 015 008) [32] (v5.1.2) assessments of the new assembly revealed 93.3% completeness (Supplementary Fig. S3). The continuity of our new assembly yielded a 62-fold improvement compared to that of BGI_duck_1.0 (76.13 vs. 1.23 Mb) (Supplementary Table S3). GCA_003850225.1 (IASCAAS_PekinDuck_PBH1.5) had fewer gaps than BGI_duck_1.0 (0.26% vs. 3.17%), which also indicated that our new assembly presented a higher level of integrity (Fig. 1B-D, Supplementary Table S3). After the annotation of the newly assembled reference genome, we obtained 22,079 annotated genes, representing an increase of 34.22% relative to BGI_duck_1.0. All of the above results indicated that the quality of our newly assembled Pekin duck genome was greatly improved compared with that of the BGI_duck_1.0 assembly. Furthermore, we mapped the resequencing data of 119 Pekin duck individuals (Supplementary Table S4) to BGI_duck_1.0 and GCA_003850225.1, and the mapping rate was significantly improved (Fig. 1E) (*t* test, *P* < 2.22e-16) when using the new assembly.

To track the dynamic change process in the Pekin duck genome driven by intense selection, we conducted the resequencing of 30 individuals (15 males, 15 females) from G1 to G10 in intervals of 3 generations. We mapped all of the paired-end reads to the GCA_003850225.1 assembly with an average coverage rate of 94.77% and an average depth of 7.08× (6.26–8.01×) (Supplementary Table S4). These sequencing data enabled us to identify a total of 8.60 million SNPs and 81 high-confidence copy number variation regions (CNVRs) among the 119 individuals.

### Breeding process in the Pekin duck Z2 line

**Figure 2:**
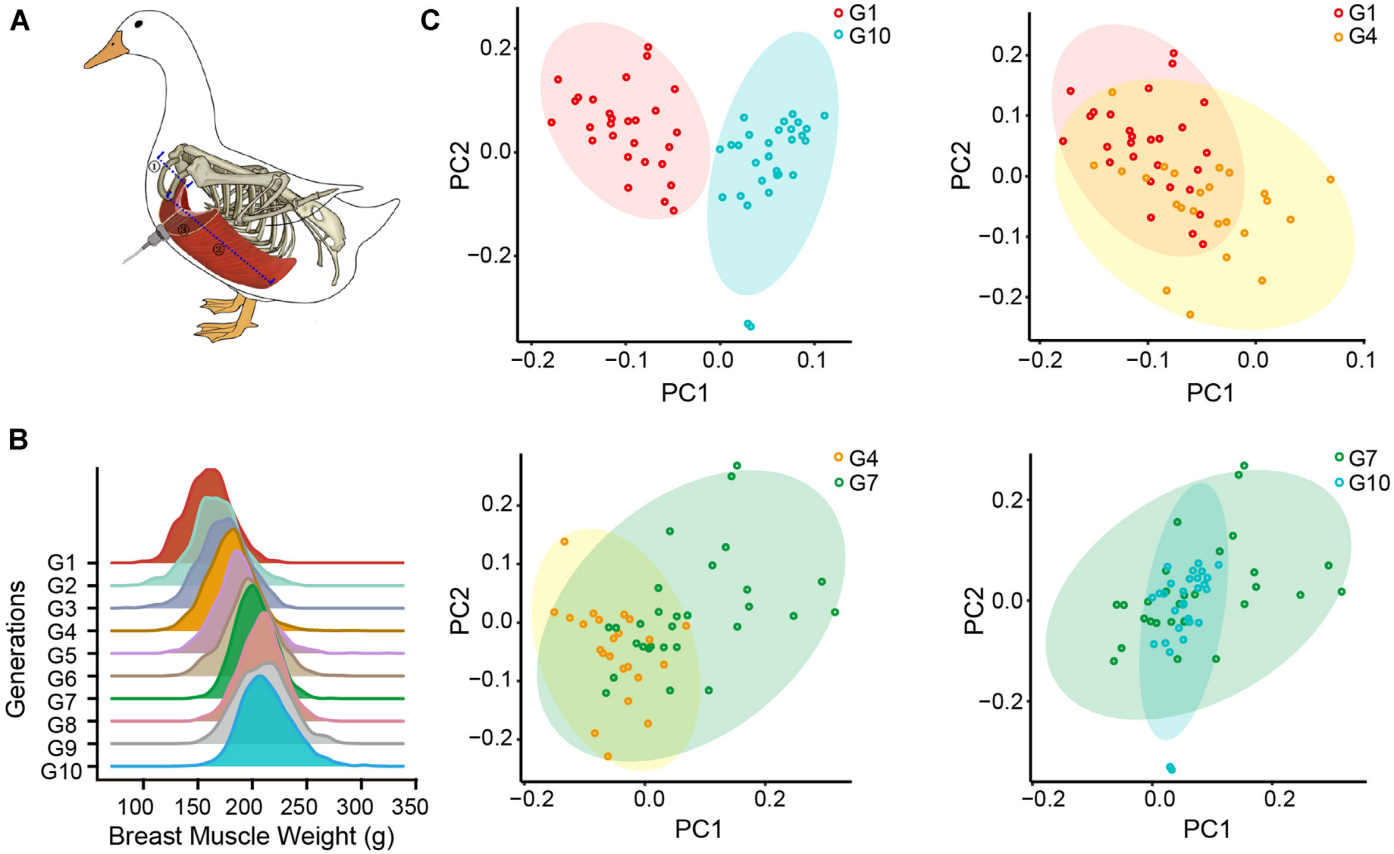
Phenotypic and population genetic structure variation over 10 generations. (A) Measurement of duck breast muscle volume (BMV) *in vivo* at 6 weeks of age. The 3 measured values were breast breadth (BB), keel length (KL), and breast muscle thickness (BMT). The weight of breast muscle was calculated based on the following formulas: BMV = BB × KL × BMT, and BMW = 0.6228 × BMV + 17.042. (B) Changes in breast muscle weight in the Pekin duck Z2 line over 10 generations. (C) Principal component analysis (PCA) of 10 generations. The red circles represent first-generation (G1) individuals, the yellow circles represent fourth-generation (G4) individuals, the green circles represent seventh-generation (G7) individuals, and the blue circles represent tenth-generation (G10) individuals.

Breast muscle weight was calculated according to the following equations. First, breast muscle volume (BMV) = BB × KL × BMT, and BMW = 0.6228 × BMV + 17.042 [33], where BB is breast breadth, KL is keel length, and BMT is breast muscle thickness. Herein, we used a Vernier caliper to measure BB and KL, while BMT was measured with B-ultrasound scanning technology (Fig. 2A). BMW was then estimated according to the BMV derived from the BB, KL, and BMT values. The statistical results showed that after 10 generations of high-intensity artificial selection, the breast muscle weight of Pekin duck increased from 80 to 220 g (Fig. 2B, Supplementary Fig. S4).

### Dynamic changes in population structure under short-term intense selection

To examine genetic differentiation at the whole genome level in 10 generations, we performed principal component analysis (PCA) [34] using the whole-genome SNP data of G1, G4, G7, and G10. Individuals across generations separated along the 2 principal component dimensions, and ducks between the G1 and G10 generations could be clearly separated into 2 clusters (Fig. 2C). In the G10 generation, population diversity showed a decreasing trend (Fig. 2C, bottom right). However, there were no significant differences in the proportions of nonsynonymous mutations and synonymous mutations (dN/dS) in the coding region between the 4 generations (Supplementary Table S5). The dN/dS ratio of all generations was below 1, indicating that the Pekin duck population was subjected to continuous negative selection.

**Figure 3:**
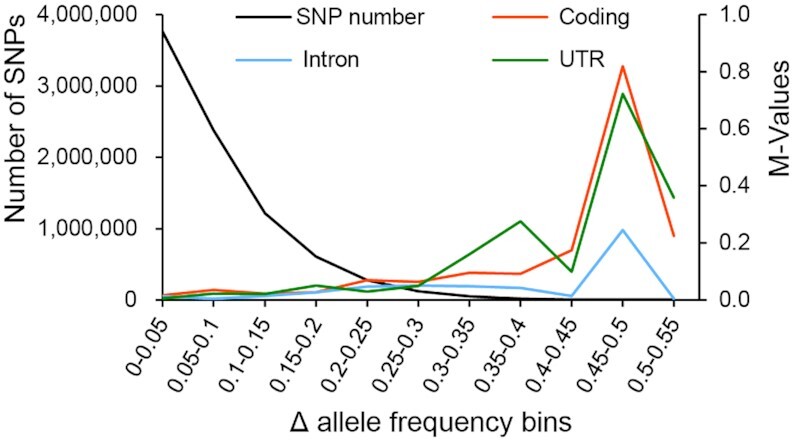
Allele frequency difference (Δ*AF*) analyses. The majority of SNPs showed low Δ*AF* values between the first- and tenth-generation ducks. The black line indicates the number of SNPs in nonoverlapping Δ*AF* bins (left y-axis). The colored lines denote the M values (log2-fold changes) of the relative frequencies of SNPs in coding regions (yellow), UTRs (green), and introns (blue), according to Δ*AF* bins (right y-axis).

To identify the dynamic changes in duck genomic variations, we calculated the allele frequency difference (Δ*AF*) between the G1 and G10 generations for each SNP and sorted these values into 5% bins (Δ*AF* = 0 to 0.05, etc.). We evaluated the enrichment of the SNPs in each bin in exons, introns, and untranslated regions (UTRs) to illustrate the numbers and distributions of sites that played a major role during artificial selection in the sequenced genomes. We observed a large number of allele frequency shifts in the entire data set, but no SNPs with Δ*AF* > 0.55 were identified (Fig. 3), implying that the directional selection event related to the breast muscle weight of Pekin ducks was in accord with polygenetic and soft selective sweep patterns [35]. We found significant enrichments of high Δ*AF* SNPs (Δ*AF* > 0.3) in both UTRs and introns ( χ^2^ test, *P* < 0.05), whereas in exons, the excess was only 2 SNPs (Fig. 3, Supplementary Table S6). We found that exonic SNPs tended to be significantly enriched in bins with Δ*AF* < 0.1 (Supplementary Table S6). Therefore, changes in noncoding regions played an important role during the breeding process in the Pekin duck Z2 line.

### Identification of signals under artificial selection

**Figure 4:**
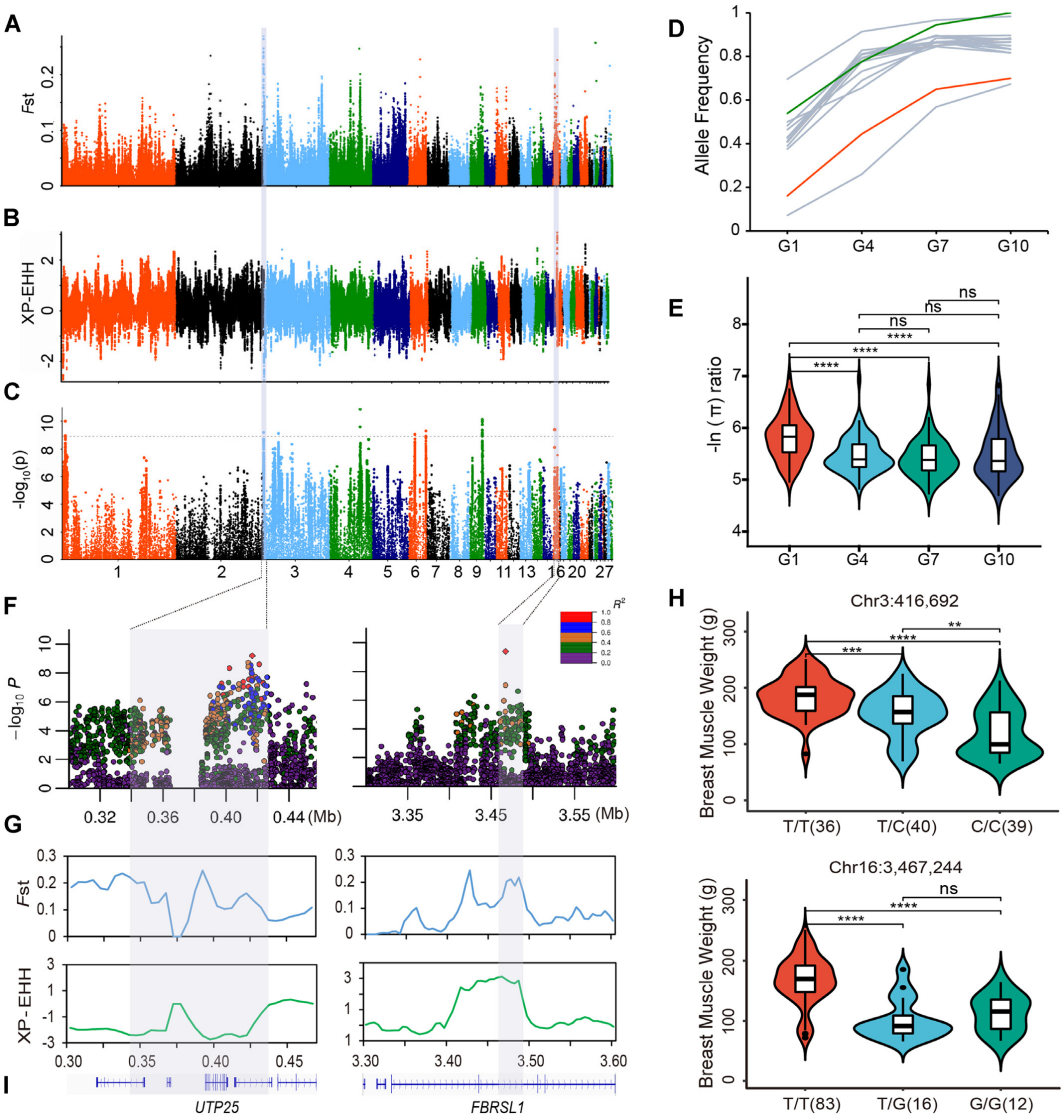
Overlapping selection signals in the genomes of the first and tenth generations. (A) Manhattan plot of selected regions between the first generation (G1) and the tenth generation (G10). Pairwise fixation index values (*F*st) are calculated in 10-kb sliding windows and 5-kb steps. The significance threshold for *F*st is 0.103 (1%). (B) Manhattan plot of selected regions between the first generation (G1) and the tenth generation (G10). Cross-population extended haplotype homozygosity (XP-EHH) values were calculated in 10-kb sliding windows and 5-kb steps. The significance thresholds for XP-EHH were 1.475 and −1.480 (1%). (C) Manhattan plots for a GWAS of breast muscle weight. The gray horizontal dashed lines indicate the Bonferroni-corrected significance threshold of the GWAS (−log_10_  *P* = 8.94), and the selection signals are indicated with a gray background. (D) Allele frequency trajectories of 22 SNPs. (E) The variation trend of genetic diversity in selected regions among generations (Chr3:0.18–0.44 Mb; Chr16:3.40–3.50 Mb). (F) Regions containing loci associated with breast muscle weight ranging from 0.18 to 0.44 Mb along chromosome 3 and 3.40 to 3.50 Mb along chromosome 16. All genotyped SNPs are color coded according to their pairwise LD, with the leader SNP (Chr3:416,692; Chr16:3,467,244) calculated by comparing the first generation (G1) and tenth-generation (G10) populations. SNPs are colored based on the strength of the LD values (*r*^2^ values) considering the most strongly associated SNP and the other SNPs in the region. (G) The blue line diagrams refer to the fixation indexes (*F*st) on selected regions (Chr3:0.18–0.44 Mb; Chr16:3.40–3.50 Mb) between G1 and G10. *F*st values are calculated in 10-kb sliding windows in 5-kb steps. The selection signals that overlapped with characterized GWAS loci are indicated with a gray background. The green line diagrams refer to XP-EHH for selected regions (Chr3:0.18–0.44 Mb; Chr16:3.40–3.50 Mb) between G1 and G10. XP-EHH values are calculated in 10-kb sliding windows in 5-kb steps. The selection signals that overlapped with characterized GWAS loci are indicated with a gray-blue background. (H) Associations between genotypes of 2 leader SNPs in candidate regions and breast muscle weight. Boxplots indicate the median (centerline), 25th to 75th percentiles (limits), and minimum and maximum values (whiskers). The indicated *P* values are based on 1-way ANOVA. ****P < 0.0001, ***P < 0.001, **P < 0.01, and ns indicates that the *P* value was not significant. (I) Schematic diagram showing the genes distributed within the candidate regions (Chr3:0.34–0.43 Mb and Chr16:3.46–3.49 Mb).

We employed a joint analysis strategy to calculate fixation index (*F*st) [36] values and cross-population extended haplotype homozygosity (XP-EHH) [37] values (10-kb window, 5-kb step) to identify potential selected regions between the G1 and G10 populations (Fig. 4A and B, Supplementary Fig. S5). Using the empirical quantiles of the top 1% of SNPs and taking the intersection of the *F*st (*F*st > 0.09) and XP-EHH (XP-EHH >1.47, XP-EHH <−1.48) analysis values, we identified a total of 187 regions as potentially containing selective signals (Supplementary Table S7). Among these candidate regions, 59 genes were found across ∼1.22 Mb (Supplementary Table S8). In addition, the results illustrated that the allele frequency of the potential selected regions identified by different approaches showed an upward trend over the 10 generations (Supplementary Fig. S6A and B). The genetic diversity of the potential selected regions exhibited the opposite change trend among different generations (Supplementary Fig. S7). To exclude the potential selected regions that may be due to genetic drift, we then employed a genome-wide association study (GWAS) to identify the overlapping selection signatures associated with breast muscle weight. We applied a Bonferroni threshold of a −log_10_  *P* > 8.94 value for outliers to identify selected SNPs associated with breast muscle weight, and a total of 22 SNPs reached the association analysis threshold (Fig. 4C, Supplementary Table S9). The allele frequencies of the 22 SNPs increased gradually over 10 generations (Fig. 4D, Supplementary Fig. S6C), indicating that these loci were subjected to continuous selection. After overlapping the associated SNPs with candidate selected regions, we identified 2 regions associated with breast muscle weight. These signals were located on chromosomes 3 and 16 (Chr3: 0.18–0.44 Mb; Chr16: 3.40–3.50 Mb). The genetic diversity of the 2 selected regions was significantly different between generations (Fig. 4E).

To accurately detect the genomic footprints left by artificial selection, we examined the linkage disequilibrium (LD, expressed as *r*^2^) of the top SNPs (Chr3:416,692 bp; Chr16:3,467,244 bp) and surrounding SNPs within these candidate regions. Then, we detected corrected signals (*r*^2^ > 0.4) in the 0.34 to 0.43 Mb region of chromosome 3 and the 3.46 to 3.49 Mb region of chromosome 16, which were associated with breast muscle weight (Fig. 4F and G). The extent of LD between the variants within the candidate regions and the lead SNPs increased gradually over 10 generations (Supplementary Fig. S8). These regions spanned 120 kb and contained 2,072 SNPs. Notably, we found that a large proportion (93.87%, 1,945/2,072) of the SNPs in the candidate regions were located in noncoding regions (Supplementary Fig. S9). Thus, continuous breeding for breast muscle weight in Pekin ducks has a polygenic basis with many loci responding to continuous artificial selection, and noncoding sequences may play an important role in Pekin duck breast muscle weight improvement.

Genotyping the ducks using the lead SNP located at Chr3:416,692 (T>C) revealed that individuals carrying the variant T alleles exhibited heavier breast muscles (Fig. 4H). The top SNP on chromosome 16 (Chr16:3,467,244 T>G) showed the same trend (Fig.   4H). In addition, 2 genes ( *UTP25* and *FBRSL1*) were identified in the 2 putative selected regions (Fig. 4I). Combined analysis with the global transcriptomic data of ducks revealed that *UTP25* and *FBRSL1* were widely expressed in various tissues of Pekin ducks (Supplementary Fig. S10). We then tracked the expression levels of *UTP25* and *FBRSL1* genes in the breast muscles of different developmental periods of the Pekin duck Z2 line. The results illustrated that the expression level of *FBRSL1* decreased with the increase of days, while *UTP25* was continuously expressed in breast muscle (Supplementary Fig. S11).

### Identification of CNVRs under artificial selection

**Figure 5:**
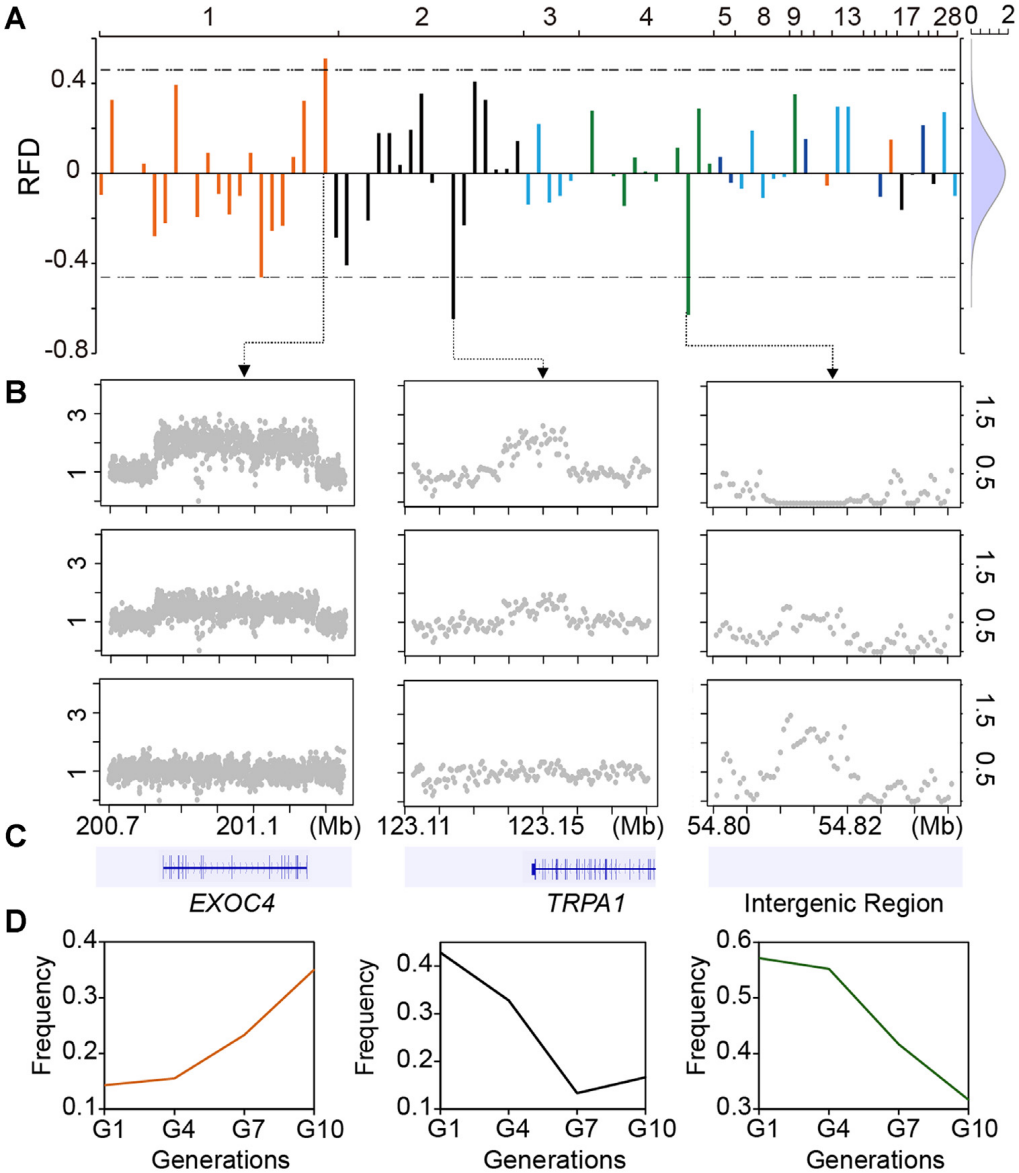
Genome-wide screening of selected copy number variations (CNVs) between the first and tenth generations. (A) The relative frequency difference (RFD) between the first generation (G1) and the tenth generation (G10) is plotted against the position on each of the autosomes. The 2 horizontal dashed lines indicate the genome-wide thresholds of selection signals, which showed the highest absolute RFD value of 5% (>4.1). (B) Examples of copy number variant form of selected CNVs. The abscissa represents the selected copy number variation region, and the ordinates on both sides represent standardized absolute copy numbers (0, homozygous deletion; 1, normal copy number, i.e., normal diploid; 0.5, loss of heterozygosity; 1.5, heterozygous repetition; 2, homozygous repetition; more than 2 denotes complex multicopy). CNV1 (Chr1:200.83–201.27 Mb) and CNV2 (Chr2:123.14–123.16 Mb) mainly consisted of multiple copies, and CNV3 (Chr4:54.81–54.82 Mb) was a deletion. (C) Schematic diagram showing the genes distributed within the candidate regions. (D) Frequency changes in candidate CNVs over 10 generations.

Copy number variations (CNVs) show higher mutational rates than SNPs [38], typically involve larger genomic regions, and potentially affect a wide range of phenotypic traits [39–41]. Based on our new assembly, we obtained 81 CNVRs with high credibility and accuracy on autosomes (Fig. 5A, Supplementary Table S10). In total, we identified 2 duplicated CNVRs and 1 deletion with significant allele frequency changes between the G1 and G10 populations (Fig. 5A and B). The CNVRs detected in most individuals were duplications located on chromosome 1 at 200.83 to 201.27 Mb (CNV1) and chromosome 2 at 123.14 to 123.16 Mb (CNV2) (Fig. 5B), and these 2 CNVRs were annotated in 2 genes, *EXOC4* and *TRPA1* (Fig. 5C). In addition, the CNVR identified in most individuals (78%) was a homozygous copy number loss or hemizygous copy number loss on chromosome 4 at 54.81 to 54.82 Mb (CNV3) (Supplementary Table S11), and the allele frequency of this CNVR increased gradually over the 10 generations (Fig. 5D).

Interestingly, the *EXOC4* gene was completely encompassed by CNV1. Combining this analysis with transcriptome data showed that *EXOC4* was widely expressed in various periods and tissues, and the expression level in breast muscle, sebum, and brain was higher than that in other tissues (Supplementary Fig. S12A). Similar to CNV1, only 1 gene, *TRPA1*, was located in CNV2. A study has shown that *TRPA1* is associated with growth traits (including body weight, body length, body height, etc.) in bovine [42]. In Pekin ducks, we did not observe the high expression level of TRPA1 in breast muscle (Supplementary Fig. S12B), but the body weight increased with the increase of breast muscle weight (Supplementary Fig. S4).

## Discussion

Sequencing technology and comparative genomics have furthered our understanding of selection and variation. Based on long-term selection and phenotypic differentiation in livestock populations, several major genes controlling economically important traits in livestock have been identified [43–46]. However, the genetic mechanism of short-term, intense artificial selection remains unclear. Our work demonstrated how the response to short-term intense artificial selection targeting a complex trait—8-week breast muscle weight—in the Pekin duck Z2 line has been predominantly achieved by recruiting a large number of loci in the genome to undergo frequency shifts. This result was supported by the conclusions of previous studies on chicken experimental models [3, 4, 13].

Based on our new assembly, we analyzed the SNP information of each generation. We found that noncoding sequences have played a prominent role during Pekin duck breeding. This is can potentially be explained as, on the one hand, compared with coding sequences, *cis*-regulatory elements of pleiotropic loci are now considered the main source of phenotypic differentiation [47–49]. And, on the other hand, mutation bias reduces the mutation rate of functionally constrained regions [50]. After a new gene is formed by gene duplication and establishes a clear function, the mutation frequency of its coding region is restricted [51]. Thus, most of the mutations found in coding regions are synonymous mutations, and the mutation frequency in these regions is lower than that in noncoding region.

We did not find completely fixed loci in the genome of the Pekin duck Z2 line, although this population was subjected to high-intensity artificial selection for 10 generations. However, we detected allele frequency shifts at many loci throughout the Pekin duck genome. Two possible explanations may account for this finding. First, it is more common for complex quantitative traits to involve a combination of multiple standing variations at many loci. Such polygenic adaptive patterns achieve fast phenotypic optimization through allele frequency shifts at many loci but do not necessarily lead to the fixation of any variation [13, 35]. Once selection pressure relaxes, the phenotype regresses toward the original (preselection) stat [52]. Second, no single genetic variation was shown to be either a necessary or sufficient condition for population breeding in this work.

In this study, we identified 2 genes, *UTP25* and *FBRSL1*, significantly associated with breast muscle weight. *FBRSL1* belongs to the Polycomb group (PcG) gene and is essential for many biological processes in mammals, including stem cell maintenance and differentiation [53, 54]. Our results showed that the expression level of *FBRSL1* in the breast muscle of Pekin duck Z2 line decreased with the increase of age. This may hint that *FBRSL1* was strongly selected to promote the development of Pekin duck breast muscle. *UTP25* (also named DIEXF in human) reportedly affects digestive organ expansion by regulating the p53 pathway [55, 56]. However, the function of *UTP25* has not been characterized in animal muscles. *UTP25* was strongly selected in the continuous breeding process of the Pekin duck Z2 line, and further investigation would be necessary to elucidate its functions.

It can be anticipated that CNVs may be more important hotspots to reveal selection-induced molecular changes in Pekin ducks. First of all, CNVs are widespread in domesticated animals, such as pigeon, sheep, pig, chicken, cattle, horse, and dog [57–66]. Moreover, the identified CNVs were largely found in genes that encode growth factors and receptors, as well as genes related to development, and they play a role mainly through duplication [67]. Second, CNVs may have a role in rapid adaptation under strong selective pressure. This phenomenon was found in our experimental population that 81 CNVs were identified in the Pekin duck genome under 10 generations of high-intensity selection. Similar phenomena were found in experimental evolution studies in microbes under nutrient limitation and multicellular systems [68–70]. Finally, and most important, there is evidence for CNVs under selection in domesticated species. During domestication, CNVs underlying domestication traits increase in frequency in the population in response to selection, and genomic signatures for selection can sometimes be detected associated with these CNVs [57, 71, 72]. In this study, we identified 2 duplication CNVRs that were annotated in 2 genes, *EXOC4* and *TRPA1*, respectively. *EXOC4* may affect the development of breast muscle in Pekin ducks by affecting glucose transport and insulin synthesis. Studies have found that *EXOC4* was involved in insulin synthesis and glucose transport in skeletal muscle [73–76]. As a component of the exocyst complex, *EXOC4* is required for targeting of Glut4 to the plasma membrane by insulin [75]. Our results showed that after 10 years of artificial selection, the number of individuals with multiple copies of *EXOC4* gene increases in the population of Pekin ducks. We speculate that the duplication makes fold increase of Exoc4 proteins, and a large number of Exoc4 proteins facilitate glucose transport to cells. Since cells become more efficient at taking up glucose, the excess glucose can be converted to fat and amino acids and stored by the body. Therefore, this may be one of the factors affecting the change of breast muscle weight of Pekin ducks. However, this requires confirmation. There is solid evidence demonstrating that *TRPA1* is expressed throughout the mammalian body and has potential beneficial effects on systemic metabolism, including glucose metabolism [77]. Growing experimental evidence suggests that *TRPA1* plays an important role in weight gain, obesity, and insulin secretion [42, 77–81]. In Pekin ducks, we did not observe the high expression level of *TRPA1* in breast muscle, but the body weight increased with the increase of breast muscle weight. Previously, our group has also demonstrated that there is a high genetic and phenotypic correlation between breast muscle weight and body weight in Pekin ducks (0.83 and 0.80) [33]. We surmise that *TRPA1* indirectly affects the breast muscle weight of Pekin ducks by affecting body weight.

In summary, our research enabled us to understand the genetic variation mechanism of farm animal genomes under intense artificial selection and will provide useful information for the establishment of an efficient molecular breeding system for livestock.

## Materials and Methods

### Subject details and sampling

All duck samples for this study were collected from Pekin Duck Breeding Base, Changping District, Beijing. The Z2 line originated from the initially conserved population of Pekin duck in Beijing. The Pekin duck Z2 line was selected as the research object because it has many characteristics, including (i) it is bred in a closed group, with a pure pedigree and a clear genetic background; (ii) the selection pressure of this line is constant, which is favorable for the accumulation of alleles gradually; (iii) at the age of 6 weeks each generation, 15 male and 15 female ducks were randomly selected from a large population for a slaughter test to measure their breast muscle weight and retain blood samples; and (iv) the breeding of all generations was completed in Pekin duck breeding base, Changping District, Beijing, and the performance measurement was done in spring, resulting in little difference in environmental effect between generations. Furthermore, all ducks were kept in a similar environment and had free access to water and feed pellets [33]. We measured the breast muscle weight of the whole population *in vivo* at the age of 6 weeks of each generation and selected ducks with the heavier breast muscle weight as parents to produce the next generation. Roughly 750 individuals in each generation were retained for breeding, with a 35% to 40% retention rate for female ducks and a 7% to 8% retention rate for male ducks. The inbreeding was strictly avoided by calculating the inbreeding coefficient of each generation. The breast muscle weight (BMW) trait was estimated by breast muscle volume (BMV), breast width (BB), keel length (KL), and breast muscle thickness (BMT) (Fig. 2A, Supplementary Fig. S4). The correlation equation between these traits was as follows: BMV = BB × KL × BMT; BMW = 0.6228 × BMV + 17.042 [19]. BB and KL were measured by vernier calipers, while BMT was measured by ultrasound scanning technology. The correlation parameters of BMW and BMV are real and reliable numerical results based on the measured breast muscle weight of years of slaughter experiments and fitted by a linear regression equation model.

In this study, we collected the blood of 30 ducks and phenotypic data of each individual (15 males and 15 females per generation) in the first, fourth, seventh, and tenth generations, respectively. A total of 120 duck samples were obtained (one sample was lost in the fourth generation), and a total of 119 samples were obtained finally.

In addition, we randomly selected an adult female Pekin duck from the Pekin duck Z2 line to collect its liver for PacBio sequencing. We also collected breast muscle tissue from a male Pekin duck for BioNano and Hi-C sequencing. Furthermore, phenotype and pedigree data from all samples were collated for subsequent analysis. All individuals used for resequencing were collected wing vein blood and rapidly frozen at −20 °C. The phenol–chloroform method was used to extract blood DNA. The quality and quantity of the DNA were examined via Nanodrop and agarose gel electrophoresis. Then, the Illumina (San Diego, CA, USA) HiSeq X Ten platform (RRID:SCR_016385) was used to sequence the paired-end sequencing libraries with an inserted fragment length of approximately 500 bp in 8× .

### Reference genome assembly and annotation

We used the combined strategy of long-reads single-molecule sequencing (PacBio, Beijing, China, RRID:SCR_017988) [82, 83], optical mapping (Bionano Genomics, Beijing, China) [84, 85], and chromosome interaction mapping (Hi-C) [86], which improved contiguity and completeness relative to the BGI_duck_1.0 [87]. We first used Canu (RRID:SCR_015880) [88] (v1.7.1) to correct and trim the subreads of PacBio with the default parameters. Then we assembled the high-quality sequence obtained in the previous step into contigs and adjusted the “correctedErrorRate” parameter to 0.05. Pilon (RRID:SCR_014731) [89] (v1.23) was used to polish the assembled contigs twice. Then scaffolds were assembled using Irys optical mapping data. We first adopted IrysSolve (Bionano Genomics) to assemble the raw Bionano data into an optical map with default parameters. Next, the runBNG pipelines [90] (v1.02) were used to construct the scaffolds based on the overlapping information between the optical map and PacBio contigs.

Then we adopted Hi-C technology to anchor the scaffolds near the chromosome level. We first used bowtie2 to align the clean Hi-C raw reads to scaffolds. We generated ∼1.06 Gb pair-end reads, and ∼593.5 million were uniquely mapped to the scaffolds (Supplementary Table S10). After filtering out reads with low mapping, multiple hits, duplications, and singletons, only valid pairs were retained for subsequent analysis. After that, HiC-Pro (RRID:SCR_017643) [91] (v2.10.0) was used to construct an interaction matrix for valid interaction pairs of ∼371.6 million, and HiCPlotter [92] (v0.8.1) was used to draw the interaction heatmap. Then, Juicer was used to align the clean Hi-C reads to the draft assembly, and then the extracted data were automatically generated into a nearly chromosomal length assembly using the 3-dimensional DNA pipeline. The final draft was corrected using PBJelly (RRID:SCR_012091) [93].

The genome assembly was annotated by the NCBI Eukaryotic Genome Annotation Pipeline [66], an automated pipeline that annotates genes, transcripts, and proteins on draft and finished genome assemblies.

### Variant calling and filtering

The raw reads from Illumina sequencing were filtered before downstream analyses by removing adapter sequences, contaminated reads, and low-quality reads. Then the reads were mapped to the assembly (GCA_003850225.1) with Burrows–Wheeler alignment (RRID:SCR_010910) [94] (v0.7.17-r1198) using the default parameters. SAMtools (RRID:SCR_002105) [95] (v1.13–14) software was used to convert mapping results into the BAM format and to filter the unmapped and nonunique reads. The paired reads that were mapped to the exact same position on the reference genome were identified with MarkDuplicates in Picard [96] (RRID:SCR_006525) to avoid any influence on variant detection. After the comparative evaluation of the depth and coverage of the results, we used the HaplotypeCaller program of GATK (RRID:SCR_001876) [97] (v1.90) software to call SNPs and indels to ensure the accuracy. In addition, this method can avoid the interference of false-positive sites in the follow-up analysis. For SNPs and indels, we restricted the variant form to biallelic variants by setting the option of GATK to "-T SelectVariants -SelectType SNP – RestrictTallelesto Biallelic." For the total variants, we set the GATK option “-T SelectVariants -select 'AF < 1.00'” to limit the allele frequency. We then filtered the output by using VCFtools (RRID:SCR_001235) [98] (v0.1.14). SNPs that did not meet the following criteria were excluded: (i) 3× < mean sequencing depth (over all included individuals) < 30×, (ii) a minor allele frequency >0.05 and a max allele frequency <0.99, (iii) maximum missing rate <0.1, and (iv) only 2 alleles.

We used the CNVcaller (RRID:SCR_015752) [99] software to detect CNVs across 119 individuals, and this method also took into account the depth of reads and pair relationship, so as to identify the CNV interval. We first specified a 1,000-bp sliding window and a 500-bp step to count the GC, repeat, and gap contents of each window in the reference genome to generate the reference genome database. Then we calculated the absolute number of copies per window. Third, we used the “CNV.Discovery.sh” script to detect the CNV of the genomes with the parameter settings “-f 0.05 -h 5 -r 0.01 -p primaryCNVR -m mergeCNVR.” Finally, we used the “Genotype.py” script to genotype the copy numbers of each sample and generate the VCF file.

### Analysis of population genetic differences

The Smartpca program of EIGENSOFT (RRID:SCR_004965) [100] (v4.2) software was used for PCA of whole-genome SNPs. We plotted the first 2 eigenvectors in 2 dimensions with our own R script for G1 and G4, G4 and G7, G7 and G10, and G1 and G10 populations, respectively.

For estimations of allele frequencies of single SNPs, we used VCFTools [98] (v0.1.14) to filter the raw sequencing data of G1 and G10 generations. The parameters are set to be “–max-missing 0.9 –maf 0.01 –min-meanDP 5 –max-meanDP 30.” After filtering, 8,433,767 reliable SNPs were obtained for allele frequency estimation. The per-SNP absolute allele frequency difference (Δ*AF*) between the G1 and G10 generations was then calculated using the following formula: Δ*AF* = abs (RefAF_G10_ − RefAF_G1_). We next binned SNPs by Δ*AF* in steps of 0.05 (i.e., Δ*AF* = 0–0.05, 0.05–0.10, etc. until 0.95–1.00) and intersected these binned SNPs with coding exons, introns, and UTRs.

### Genome-wide association analysis of traits

A GWAS was performed using a mixed linear model of EMMAX [101] program using genome-wide SNP data and breast muscle weight of 119 individuals from the resequenced population. The analysis model was


\begin{equation*} y = Xb + Ga{\mathrm{ }} + e. \end{equation*}

where *y* is the phenotypic value (the breast muscle weight of per duck), *X* is the matrix corresponding to the fixed effect, and *b* is the fixed effect size. Fixed effects include sex effects. *G* is the genetic matrix corresponding to the population kinship, and *e* is the random residual. PCA was performed based on all SNPs, and the top 3 components were set as fixed effects in the mixed model to correct for population stratification. We defined a Bonferroni correction threshold of 0.01/N (−log_10_  *P* = 8.94) to identify the significant loci of the GWAS results, where *N* was the number of whole-genome SNPs.

For the identified associated genes, we also referred to the Pekin duck Panoramic transcription map (transcriptome data of all tissues in the 3 development stages) established by our previous study [46] to check whether these genes were expressed in the development stage of breast weight muscle, so as to further confirm that they did participate in the regulation of breast muscle development.

### Identification of selected regions

We used the VCFtools [98] (v0.1.14) software to calculate *F*st between G1 and G10 generations by selecting parameters of 10-kb windows with a 5-kb step size. We used selscan software [102] (v1.2.0a) to calculate the XP-EHH values of G1 and G10 groups with the same parameter settings of 10-kb sliding windows and 5-kb step size, in which the G1 generation was taken as the reference group and the G10 generation as the query group. The overlapping regions of 2 windows with statistical values above the top 1% of the quantile would be selected as preliminary candidate regions.

### Linkage disequilibrium analysis

In addition, to further narrow the candidate interval, we also used the Haploview (RRID:SCR_003076) [103] to analyze the linkage disequilibrium of the candidate region (related to Fig. 4). We adopted the squared correlation coefficient (*r*^2^) as the coefficient to measure the linkage disequilibrium between the leader SNP and the surrounding SNPs. The parameters settings were “–ld-window 99999 –ld-window-kb 1000 –ld-window-r2 0 –r2.” Then we integrated and plotted the LD data with GWAS results (related to Supplementary Fig. S8).

### Identification of selected CNVRs

Based our new assembly, we used the CNV caller [99] to identify genome-wide CNVs and CNVRs. To avoid false positives, 2 parameters, silhouette coefficient (>0.7) and minor allele frequency (>0.05) were adopted to filter the CNVRs obtained, and then we got 81 CNVRs with high credibility and accuracy on autosomes. Subsequently, we used the relative frequency difference (RFD) [104] to detect the CNVs that occurred on the Pekin duck genome during population differentiation. After that, we adopted the topmost 5% RFD value (absolute RFD value >4.1) as the threshold to screen the potentially selected CNVRs.

## Supplementary Material

giad016_GIGA-D-22-00268_Original_Submission

giad016_GIGA-D-22-00268_Revision_1

giad016_Response_to_Reviewer_Comments_Original_Submission

giad016_Reviewer_1_Report_Original_Submission Gene Ng -- 11/15/2022 Reviewed

giad016_Reviewer_1_Report_Revision_1 Gene Ng -- 2/17/2023 Reviewed

giad016_Reviewer_2_Report_Original_Submission Surya kanta Mishra -- 12/11/2022 Reviewed

giad016_Supplemental_Figures_and_Tables

## Data Availability

The assembly and annotation of Pekin duck has been deposited in GenBank under the Bioproject accession code PRJNA496533 (accession No. RHJV01000000). All supporting data are available in the *GigaScience* GigaDB database [105].

## Additional Files

**Supplemental Fig. S1**. The pipeline for multilevel chromosome assembly. Canu was used for constructing initial contigs. Then, polishing was performed with Pilon using PacBio-only long reads. Hybrid scaffolding of the PacBio-corrected contigs and the BioNano-based consensus map was performed using the hybrid scaffolding module within runBNG software. The Hi-C sequencing data were first aligned to the assembled contigs/scaffolds using the Bowtie end-to-end algorithm, and then the assembled scaffolds were clustered, ordered, and directed into a chromosome level using 3-dimensional DNA. The final draft was corrected using PBJelly.

**Supplemental Fig. S2**. Hi-C interactions among 29 chromosomes with a 40-kb resolution.

**Supplemental Fig. S3**. BUSCO completeness assessment for the new assembly. In summary, it covered 93.3% (7,780/8,338) of complete BUSCO genes and 1.3% (111/8,338) of fragmented BUSCO genes.

**Supplemental Fig. S4**. Generational average phenotypic values of prime traits of the Pekin duck Z2 line. The abscissa denotes the generation.

**Supplemental Fig. S5**. Distribution of *F*st and XP-EHH of 10-kb windows size for whole-genome-wide variants between the G1 and G10 generations. Bins of *F*st and XP-EHH are presented along the x-axes. μ, mean; δ, standard deviation.

**Supplemental Fig. S6**. The variation of allele frequency of putative regions over 10 generations (corresponding to Fig. 2). (A, B) Red and green lines indicate the frequency variation of representative loci in selective regions of *F*st and XP-EHH, respectively (corresponding to a 1% significance level of *F*st in 10-kb sliding windows and a 1% significance level of XP-EHH in 10-kb sliding windows; each line represents the top SNP of a sliding window). (C) Blue lines indicate the frequency variation of 22 SNPs that reached the Bonferroni significance threshold of the GWAS (−log_10_  *P* > 8.94).

**Supplementary Fig. S7**. The variation trend of genetic diversity in potentially selective regions among generations. This figure shows the trend of genetic diversity in the common selection signatures obtained by *F*st and XP-EHH tests across generations. The indicated *P* values are based on 1-way ANOVA. ***P* < 0.01, **P* < 0.05, and ns indicates that the *P* value was not significant.

**Supplemental Fig. S8**. Linkage disequilibrium (LD) in 2 candidate regions of 10 generations. Each diamond contains a level of LD (*r*^2^) between all SNP pairs.

**Supplemental Fig. S9**. SNP distribution in candidate regions.

**Supplemental Fig. S10**. Expression levels of the *UTP25* and *FBRSL1* genes in different tissues of Pekin duck. (A) *FBRSL1* expression levels in Pekin duck tissues. (B) *UTP25* expression levels in Pekin duck tissues. The expression data were obtained from a global gene expression database for ducks generated from transcriptome analyses. The database lists the expression levels of all genes in breast muscle, skin, liver, fat (abdominal fat), brain, heart, kidney, lung, spleen, sternum, and shank tissues at different developmental periods in Pekin ducks.

**Supplemental Fig. S11**. Expression levels of the *UTP25* and *FBRSL1* genes at different developmental periods of the Pekin duck Z2 line.

**Supplemental Fig. S12**. Expression levels of the *EXOC4* and *TRPA1* genes in different tissues of Pekin duck. (A) *EXOC4* expression levels in Pekin duck tissues. (B) *TRPA1* expression levels in Pekin duck tissues. The expression data were obtained from a global gene expression database for ducks generated from transcriptome analyses. The database lists the expression levels of all genes in breast muscle, skin, liver, fat (abdominal fat), brain, heart, kidney, lung, spleen, sternum, and shank tissues at different developmental periods in Pekin ducks.

**Supplemental Table S1**. Summary of sequencing data.

**Supplemental Table S2**. Summary of Hi-C reads mapping results.

**Supplemental Table S3**. Comparison of genome assemblies between the GCA_003850225.1 and BGI_duck_1.0.

**Supplemental Table S4**. Number of ducks used for 10 generations for the genome resequencing and mapping summary.

**Supplemental Table S5**. Genomic SNP statistics.

**Supplemental Table S6**. Distributions of SNP counts in the different delta allele frequency bins for coding sequences, introns, and UTRs.

**Supplemental Table S7**. Overlapping regions between the analysis results of *F*st and XP-EHH. Pairwise fixation index values (*F*st) and cross-population extended haplotype homozygosity (XP-EHH) values were calculated in 10-kb windows sliding in 5-kb steps. The top 1% of the overlap regions between the analysis results of *F*st and XP-EHH were considered candidate regions.

**Supplemental Table S8**. Summary of putative regions on the genome. These regions sum to 2.06 Mb, and ∼2.14 Mb were annotated into 65 genes.

**Supplemental Table S9**. The significant SNPs of GWAS analyses. We performed GWAS analyses of breast muscle weight. The Bonferroni significance threshold of the GWAS was −log_10_  *P* > 8.94. There were 22 SNPs that passed the Bonferroni significance threshold. These SNPs are located in noncoding sequences.

**Supplemental Table S10**. Putative CNVRs under artificial selection.

**Supplemental Table S11**. The copy number variation genotypes of 3 candidate CNVRs in each individual.

## Abbreviations

BB: breast breadth; BMT: breast muscle thickness; BMV: breast muscle volume; BMW: breast muscle weight; bp: base pairs; BUSCO: Benchmarking Universal Single-Copy Orthologs; BWA: Burrows–Wheeler aligner; CNV: copy number variation; CNVR: copy number variation region;*F*st: fixation index; g: gram; G: generation; Gb: gigabase pairs; GC: guanine-cytosine; GWAS: genome-wide association study; Hi-C: High-throughput Chromosome Conformation Capture; kb: kilobase pairs; KL: keel length; LD: linkage disequilibrium; Mb: megabase pairs; NCBI: National Center for Biotechnology Information; PCA: principal component analysis; RFD: relative frequency difference; SNP: single-nucleotide polymorphism; TE: transposable element; UTR: untranslated regions; XP-EHH: population extended haplotype homozygosity; Δ*AF*: allele frequency difference.
