# Supplementary material for: Resequencing of a Pekin duck breeding population provides insights into the genomic response to short-term artificial selection
Source: Gigascience. 2023 Mar 27;12:giad016. doi: 10.1093/gigascience/giad016 (PMC10041536; doi:10.1093/gigascience/giad016)

GigaScience

Resequencing of a Pekin duck breeding population provides insights into the genomic response to short-term artificial selection

--Manuscript Draft--

Manuscript Number:	GIGA-D-22-00268
Full Title:	Resequencing of a Pekin duck breeding population provides insights into the genomic response to short-term artificial selection
Article Type:	Research
Funding Information:	the CAAS Innovation Team Project (ASTIP-IAS-9, CAAS-ZDRW202104) — Not applicable<br>National Natural Science Foundation of China (31972523) — Prof. Zhengkui Zhou<br>National Ten Thousand Talent Program — Prof. Zhengkui Zhou<br>China Agricultural Research System (CARS-42-5) — Not applicable
Abstract:	Background<br><br>Short-term, intense artificial selection drives fast phenotypic changes in domestic animals and leaves imprints on their genomes. However, the genetic basis of this selection response is poorly understood. Here, we employed the Pekin duck Z2 pure line, in which the breast muscle weight was increased nearly three fold after ten generations of breeding. We de - novo assembled a high-quality reference genome of a female Pekin duck of this line (GCA_003850225.1) and identified 8.60 million genetic variants in 119 individuals among 10 generations of the breeding population.<br><br>Results<br><br>We identified 53 selected regions between the first and tenth generations, and 93.8% of the identified variations were enriched in regulatory and noncoding regions. Integrating the selection signatures and genome-wide association approach, we found that two regions covering 0.36 Mb containing UTP25 and FBRSL1 were most likely to contribute to breast muscle weight improvement. The major allele frequencies of these two loci increased gradually with each generation following the same trend. Additionally, we found that a copy number variation region containing the entire EXOC4 gene could explain 1.9% of the variance in breast muscle weight, indicating that the nervous system may play a role in economic trait improvement.<br><br>Conclusions<br><br>Our study not only provides insights into genomic dynamics under intense artificial selection but also provides resources for genomics-enabled improvements in duck breeding.
Corresponding Author:	Zhengkui Zhou<br>CAAS IAS: Chinese Academy of Agricultural Sciences Institute of Animal Science<br>Beijing, CHINA
Corresponding Author Secondary Information:	
Corresponding Author's Institution:	CAAS IAS: Chinese Academy of Agricultural Sciences Institute of Animal Science
Corresponding Author's Secondary Institution:	
First Author:	Simeng Yu
First Author Secondary Information:	
Order of Authors:	Simeng Yu

Powered by Editorial Manager® and ProduXion Manager® from Aries Systems Corporation

	Zihua Liu
	Ming Li
	Dongke Zhou
	Ping Hua
	Hong Cheng
	Wenlei Fan
	Yaxi Xu
	Dapeng Liu
	Suyun Liang
	Yunsheng Zhang
	Ming Xie
	Jing Tang
	Yu Jiang
	Shuisheng Hou
	Zhengkui Zhou
**Order of Authors Secondary Information:**	
**Additional Information:**	
**Question**	**Response**
Are you submitting this manuscript to a special series or article collection?	No
**Experimental design and statistics**<br><br>Full details of the experimental design and statistical methods used should be given in the Methods section, as detailed in our Minimum Standards Reporting Checklist. Information essential to interpreting the data presented should be made available in the figure legends.<br><br>Have you included all the information requested in your manuscript?	Yes
**Resources**<br><br>A description of all resources used, including antibodies, cell lines, animals and software tools, with enough information to allow them to be uniquely identified, should be included in the Methods section. Authors are strongly encouraged to cite Research Resource	Yes

Identifiers (RRIDs) for antibodies, model organisms and tools, where possible.<br><br>Have you included the information requested as detailed in our Minimum Standards Reporting Checklist?	
**Availability of data and materials**<br><br>All datasets and code on which the conclusions of the paper rely must be either included in your submission or deposited in publicly available repositories (where available and ethically appropriate), referencing such data using a unique identifier in the references and in the “Availability of Data and Materials” section of your manuscript.<br><br>Have you have met the above requirement as detailed in our Minimum Standards Reporting Checklist?	Yes

Manuscript
Click here to access/download;Manuscript;genetic basis of artificial selection-0928.docx

Resequencing of a Pekin duck breeding population provides insights into the genomic response to short-term artificial selection

Authors

Simeng Yu[1], Zihua Liu[2], Ming Li[2], Dongke Zhou[2], Ping Hua[2], Hong Cheng[2], Wenlei Fan[1], Yaxi Xu[1], Dapeng Liu[1], Suyun Liang[1], Yunsheng Zhang[1], Ming Xie[1], Jing Tang[1], Yu Jiang[2], Shuisheng Hou[1], Zhengkui Zhou[1*]

[1]State Key Laboratory of Animal Nutrition; Key Laboratory of Animal (Poultry) Genetics Breeding and Reproduction, Ministry of Agriculture and Rural Affairs; Institute of Animal Science, Chinese Academy of Agricultural Sciences, Beijing 100193, China

[2]Key Laboratory of Animal Genetics, Breeding and Reproduction of Shaanxi Province, College of Animal Science and Technology, Northwest A&F University, Yangling 712100, China

[*]Corresponding author. E-mail: zhouzhengkui@caas.cn.

Abstract

Background: Short-term, intense artificial selection drives fast phenotypic changes in domestic animals and leaves imprints on their genomes. However, the genetic basis of this selection response is poorly understood. Here, we employed the Pekin duck Z2 pure line, in which the breast muscle weight was increased nearly three fold after ten generations of breeding. We *de-novo* assembled a high-quality reference genome of a female Pekin duck of this line (GCA_003850225.1) and identified 8.60 million genetic variants in 119 individuals among 10 generations of the breeding population. **Results:** We identified 53 selected regions between the first and tenth generations, and 93.8% of the identified variations were enriched in regulatory and noncoding regions. Integrating the selection signatures and genome-wide association approach, we found that two regions covering 0.36 Mb containing *UTP25* and *FBRSL1* were most likely to contribute to breast muscle weight improvement. The major allele frequencies of these two loci increased gradually with each generation following the same trend. Additionally, we found that a copy number variation region containing the entire *EXOC4* gene could explain 1.9% of the variance in breast muscle weight, indicating that the nervous system may play a role in economic trait improvement. **Conclusions:** Our study not only provides insights into genomic dynamics under intense artificial selection but also provides resources for genomics-enabled improvements in duck breeding.

Keywords: duck; genome; artificial selection; breast muscle weight; selection signatures

Background

Genetic changes underlying long-term selection in animals have been well studied[1-3]. However, the genetic basis of short-term, intense artificial selection remains poorly understood. Although many studies have elucidated genetic mechanisms involved in intense artificial selection[4-8], most of them have not been genome-scale investigations.

Studies of two representative bidirectional selected resource populations in which chicken body weight[9-13] and abdominal fat[14-18] were selected have shown that continuous artificial selection results in obvious phenotypic and genomic differentiation in poultry. Artificial selection signals have also been identified in animals, such as rabbits[1], sheep[19], goats[20, 21] and pigs[22]. However, many domestic animal populations established for selection experiments are not as complete as chicken populations because of the long generation interval and sequencing costs in these species. Ducks (*Anas platyrhynchos*) are among the most economically important waterfowl; they can provide meat, eggs and down for humans, and show important characteristics, such as a short generation interval, high reproductive ability and a long but traceable history of artificial selection[23]. Moreover, ducks, like other birds, have smaller genomes than nonavian terrestrial vertebrates. These characteristics make the duck an effective model for studying genomic footprints of artificial selection.

High-quality reference genomes are the foundation for genetic research and molecular marker breeding, which can support innovations in sustainable animal production[24, 25]. To date, reference genomes have been published for a variety of domestic animals such as ducks, chickens, pigs, cattle, sheep and goats [2, 23, 26-29], providing important resources for livestock and poultry genetic breeding. To completely capture the variation present in the genome of our study population, we chose a female individual from our population for PacBio long-read sequencing and genome assembly to obtain a high-quality chromosome-level reference genome of Pekin duck (GCA_003850225.1) (**Fig. 1A**).

Herein, we adopted a Pekin duck pure line selected for breast muscle weight at 6 weeks of age. The foundation stock was the local conserved population in Beijing, China. After 10 generations of high-intensity artificial selection, the breast muscle weight of the Pekin duck Z2 line increased from 80 g to 220 g. We implemented the whole-genome resequencing of 119 ducks across 10

generations (one generation per year, 2005-2014) and mapped the resequencing data to the high-quality reference genomes assembled in this study. These data were investigated to elucidate the dynamic response patterns in the Pekin duck genome under artificial selection.

Data Description

To understand the genetic basis of short-term, intense artificial selection, we adopted a Pekin duck pure line selected for breast muscle weight at 6 weeks of age. We implemented the whole-genome resequencing of 119 ducks across 10 generations (one generation per year, 2005-2014) and mapped the resequencing data to the high-quality reference genomes assembled in this study. Using 8.60 million of single nucleotide polymorphisms (SNPs), we tested for dynamic changes in population structure under short-term intense selection and we assessed the genome for signatures of short-term intense selection associated with breast muscle weight.

Analyses

An improved Pekin duck genome assembly

To carry out the *de novo* assembly of the Pekin duck genome, we adopted a combination of PacBio long-read sequencing, BioNano optical mapping and Hi-C technologies. We first generated 65.9 Gb of PacBio long reads with 50× genome coverage, 49 Gb of BioNano high-quality reads with 41× genome coverage, and 106 Gb of Hi-C reads with 82× genome coverage (**Table S1**). Then, these data were used to assemble the new duck genome (**Fig. 1A**). Assembly was performed in a stepwise fashion (**Supplementary Fig. S1**), to generate assemblies with improvements for each process. First, we used the initial PacBio subreads to construct 2682 contigs, yielding a contig N50 of 4.17 Mb (**Table 1**). Second, we used optical mapping (BioNano Genomics Irys) data to assist in splicing the PacBio contigs into scaffolds, resulting in the population of 1,788 scaffolds, and the N50 was 6.24 Mb (**Table 1**). Then, the Hi-C data (**Supplementary Table S2**) were used to cluster the scaffolds at the chromosomal scale, resulting in 1852 scaffolds (**Supplementary Fig. S2**). The final assembled genome length was 1.12 Gb, with scaffold N50 and contig N50 values of 76.13 Mb and 4.10 Mb, respectively (**Table 1**).

Assembly	Number of scaffolds	Scaffold N50 (Mb)	Genome size
PacBio	—	—	1,154,570,803
PacBio+BioNano	1788	6.24	1,153,069,495
PacBio+BioNano+Hi-C	1852	41.14	1,134,893,859
GCA_003850225.1	1330	76.13	1,134,894,103

We next evaluated the quality of the new assembly. BUSCO (BUSCO, RRID:SCR 015008)[30] (v5.1.2) assessments of the new assembly revealed 93.3% completeness (**Supplementary Fig. S3**). The continuity of our new assembly yielded a 62-fold improvement compared to that of BGI_duck_1.0 (76.13 vs. 1.23 Mb) (**Supplementary Table S3**). GCA_003850225.1 (IASCAAS_PekinDuck_PBH1.5) had fewer gaps than BGI_duck_1.0 (0.26% vs. 3.17%), which also indicated that our new assembly presented a higher level of integrity (**Fig. 1B-D, Supplementary Table S3**). After the annotation of the newly assembled reference genome, we obtained 22,079 annotated genes, representing an increase of 34.22% relative to BGI_duck_1.0. All of the above results indicated that the quality of our newly assembled Pekin duck genome was greatly improved compared with that of the BGI_duck_1.0 assembly. Furthermore, we mapped the resequencing data of 119 Pekin duck individuals (**Supplementary Table S4**) to BGI_duck_1.0 and GCA_003850225.1, and the mapping rate was significantly improved (**Fig. 1E**) (t test, $P <$ 2.22e-16) when using the new assembly.

To track the dynamic change process in the Pekin duck genome driven by intense selection, we conducted the resequencing of 30 individuals (15 males, 15 females) from G1 to G10 in intervals of 3 generations. We mapped all of the paired-end reads to the GCA_003850225.1 assembly with an average coverage rate of 94.77%, and an average depth of 7.08 × (6.26-8.01 ×) (**Supplementary Table S4**). These sequencing data enabled us to identify a total of 8.60 million single-nucleotide polymorphisms (SNPs) and 81 high-confidence copy number variation regions (CNVRs) among the 119 individuals.

Breeding process in the Pekin duck Z2 line

Breast muscle weight was calculated according to the following equations. First, breast muscle volume (BMV) = BB×KL×BMT, and BMW = 0.6228 × BMV + 17.042[31], where BB is breast breadth, KL is keel length, and BMT is breast muscle thickness. Herein, we used a Vernier caliper

to measure BB and KL, while BMT was measured with B-ultrasound scanning technology (**Fig. 2A**). BMW was then estimated according to the BMV derived from the BB, KL, and BMT values. The statistical results showed that after ten generations of high-intensity artificial selection, the breast muscle weight of Pekin duck increased from 80 g to 220 g (**Fig. 2B, Supplementary Fig. S4**).

Dynamic changes in population structure under short-term intense selection

To examine genetic differentiation at the whole genome level in ten generations, we performed principal component analysis (PCA)[32] using the whole-genome SNP data of G1, G4, G7 and G10. Individuals across generations separated along the two principal component dimensions, and ducks between the G1 and G10 generations could be clearly separated into two clusters (**Fig. 2C**). In the G10 generation, population diversity showed a decreasing trend (**Fig. 2C, bottom right**). However, there were no significant differences in the proportions of nonsynonymous mutations and synonymous mutations (dN/dS) in the coding region between the four generations (**Supplementary Table S5**). The dN/dS ratio of all generations was below 1, indicating that the Pekin duck population was subjected to continuous negative selection.

To identify the dynamic changes in duck genomic variations, we calculated the allele frequency difference (ΔAF) between the G1 and G10 generations for each SNP and sorted these values into 5% bins (ΔAF = 0 to 0.05, etc.). We evaluated the enrichment of the SNPs in each bin in exons, introns, and untranslated regions (UTRs) to illustrate the numbers and distributions of sites that played a major role during artificial selection in the sequenced genomes. We observed a large number of allele frequency shifts in the entire data set, but no SNPs with $\Delta AF > 0.55$ were identified (**Fig. 3**), implying that the directional selection event related to the breast muscle weight of Pekin ducks was in accord with polygenetic and soft selective sweep patterns[33]. We found significant enrichments of high ΔAF SNPs ($\Delta AF > 0.3$) in both UTRs and introns (χ^2 test, $P < 0.05$), whereas in exons, the excess was only 2 SNPs (**Fig. 3, Supplementary Table S6**). We found that exonic SNPs tended to be significantly enriched in bins with $\Delta AF < 0.1$ (**Supplementary Table S6**). Therefore, changes in noncoding regions played an important role

during the breeding process in the Pekin duck Z2 line.

Identification of signals under artificial selection

We employed a joint analysis strategy to calculate fixation index (*F*st)[34] values and cross-population extended haplotype homozygosity (XP-EHH)[35] values (10 kb window, 5 kb step) to identify potential selected regions between the G1 and G10 populations (**Fig. 4A and B, Supplementary Fig. S5**). Using the empirical quantiles of the top 1% of SNPs and taking the intersection of the *F*st (*F*st > 0.09) and XP-EHH (XP-EHH > 1.47, XP-EHH < -1.48) analysis values, we identified a total of 187 regions as potentially containing selective signals (**Supplementary Fig. S6**, **Supplementary Table S7**). Among these candidate regions, 59 genes were found across ~1.22 Mb (**Supplementary Table S8**). In addition, the results illustrated that the allele frequency of the potential selected regions identified by different approaches showed an upward trend over the ten generations (**Supplementary Fig. S7A and B**). The genetic diversity of the potential selected regions exhibited the opposite change trend among different generations (**Supplementary Fig. S8**). To exclude the potential selected regions that may be due to genetic drift, we then employed a genome-wide association study (GWAS) to identify the overlapping selection signatures associated with breast muscle weight. We applied a Bonferroni threshold of a $-\log_{10} P > 8.94$ value for outliers to identify selected SNPs associated with breast muscle weight, and a total of 22 SNPs reached the association analysis threshold (**Fig. 4C, Supplementary Table S9**). The allele frequencies of the 22 SNPs increased gradually over 10 generations (**Fig. 4D, Supplementary Fig. S7C**), indicating that these loci were subjected to continuous selection. After overlapping the associated SNPs with candidate selected regions, we identified 2 regions associated with breast muscle weight. These signals were located on chromosomes 3 and 16 (Chr3: 0.18-0.44 Mb; Chr16: 3.40-3.50 Mb). The genetic diversity of the two selected regions was significantly different between generations (**Fig. 4E**).

To accurately detect the genomic footprints left by artificial selection, we examined the linkage disequilibrium (LD, expressed as r^2) of the top SNPs (Chr3:416,692 bp; Chr16:3,467,244 bp) and surrounding SNPs within these candidate regions. Then, we detected corrected signals ($r^2 > 0.4$) in the 0.34-0.43 Mb region of chromosome 3 and the 3.46-3.49 Mb region of chromosome 16, which were associated with breast muscle weight (**Fig. 4F and G, Supplementary Fig. S9**). The extent

of LD between the variants within the candidate regions and the lead SNPs increased gradually over 10 generations (**Supplementary Fig. S10**). These regions spanned 120 kb and contained 2,072 SNPs. Notably, we found that a large proportion (93.87%, 1,945/2,072) of the SNPs in the candidate regions were located in noncoding regions (**Supplementary Fig. S11**). Thus, continuous breeding for breast muscle weight in Pekin ducks has a polygenic basis with many loci responding to continuous artificial selection and noncoding sequences may play an important role in Pekin duck breast muscle weight improvement.

Genotyping the ducks using the lead SNP located at Chr3:416,6929 (T>C) revealed that individuals carrying the variant T alleles exhibited heavier breast muscles (**Fig. 4I**). The top SNP on chromosome 16 (Chr16:3,467,244 T>G) showed the same trend (**Fig. 4I**). In addition, two genes (*UTP25* and *FBRSL1*) were identified in the two putative selected regions **(Fig. 4H**). Combined analysis with the global transcriptomic data of ducks, revealed that *UTP25* and *FBRSL1* were widely expressed in various tissues of Pekin ducks (**Supplementary Fig. S12**).

Identification of CNVRs under artificial selection

Copy number variations (CNVs) show higher mutational rates than SNPs[36], typically involve larger genomic regions and potentially affect a wide range of phenotypic traits[37-39]. Based on our new assembly, we obtained 81 CNVRs with high credibility and accuracy on autosomes (**Fig. 5A, Supplementary Table S10**). In total, we identified 2 duplicated CNVRs and 1 deletion between the G1 and G10 populations (**Fig. 5A and B**). The CNVRs detected in most individuals were duplications located on chromosome 1 at 200.83-201.27 Mb (CNV1) and chromosome 2 at 123.14-123.16 Mb (CNV2) (**Fig. 5B**), and these two CNVRs were annotated in two genes *EXOC4* and *TRPA1* (**Fig. 5C**). In addition, the CNVR identified in most individuals (78%) was a homozygous copy number loss or hemizygous copy number loss on chromosome 4 at 54.81-54.82 Mb (CNV3) (**Supplementary Table S11**) and the allele frequency of this CNVR increased gradually over the 10 generations (**Fig. 5D**).

Interestingly, the *EXOC4* gene was completely encompassed by CNV1. Combined with analysis with transcriptome data, showed that *EXOC4* was highly expressed in the brains of Pekin ducks (**Supplementary Fig. S13A**). *EXOC4* is mainly expressed in the central nervous system and is significantly correlated with intelligence and cognitive ability in humans[40]. Accordingly, we

speculated that *EXOC4* may be selected because of its influence on the intelligence of Pekin ducks to contribute to intensive production. This is to be expected, given that many genes affecting brain and neuronal development have been targeted during animal domestication and improvement to meet the human needs of animal production and allow animals to tolerate the environment provided by humans [1]. Similar to CNV1, only one gene *TRPA1* was located in CNV2. Previous studies have suggested that *TRPA1* regulates gastrointestinal motility through serotonin release from enterochromaffin cells[41]. Our results indicated that *TRPA1* was significantly expressed in the kidneys of Pekin ducks at 8 weeks of age (**Supplementary Fig. S13B**).

Discussion

Sequencing technology and comparative genomics have furthered our understanding of selection and variation. Based on long-term selection and phenotypic differentiation in livestock populations, several major genes controlling economically important traits in livestock have been identified[42-45]. However, the genetic mechanism of short-term, intense artificial selection remains unclear. Our work demonstrated how the response to short-term intense artificial selection targeting a complex trait—8-week breast muscle weight—in the Pekin duck Z2 line has been predominantly achieved by recruiting a large number of loci in the genome to undergo frequency shifts. This result was supported by the conclusions of previous studies on chicken experimental models[13, 46, 47].

Based on our new assembly, we analyzed the SNP information of each generation. We found that noncoding sequences have played a prominent role during Pekin duck breeding. This is not difficult to understand. On the one hand, compared with coding sequences, *cis*-regulatory elements of pleiotropic loci are now considered to be the main source of phenotypic differentiation[48-50]. On the other hand, mutation bias reduces the mutation rate of functionally constrained regions[51]. After a new gene is formed by gene duplication, and establishes a clear function, the mutation frequency of its coding region is restricted[52]. Thus, most of the mutations found in coding regions are synonymous mutations, and the mutation frequency in these regions is lower than that in noncoding region.

We did not find completely fixed loci in the genome of the Pekin duck Z2 line, although this population was subjected to high intensity artificial selection for ten generations. However, we

detected allele frequency shifts at many loci throughout the Pekin duck genome. Two possible explanations may account for this finding. First, it is more common for complex quantitative traits to involve a combination of multiple standing variations at many loci. Such polygenic adaptive patterns achieve fast phenotypic optimization through allele frequency shifts at many loci, but do not necessarily lead to the fixation of any variation[13, 33]. Once selection pressure relaxes, the phenotype regresses toward the original (preselection) stat[53]. Second, no single genetic variation was shown to be either a necessary or sufficient condition for population breeding in this work. In summary, our research enabled us to understand the genetic variation mechanism of farm animal genomes under intense artificial selection, and will provide useful information for the establishment of an efficient molecular breeding system for livestock.

Methods

1. Subject details and sampling

All duck samples for this study were collected from Pekin Duck Breeding Base, Changping District, Beijing. Z2 line originated from the initial conserved population of Pekin duck in Beijing. Pekin duck Z2 line was selected as the research object, because it has many characteristics, including: **a** it is bred in closed group, with pure pedigree and clear genetic background; **b** the selection pressure of this line is constant, which is favorable for the accumulation of alleles gradually; **c** at the age of 6 weeks each generation, 15 male and 15 female ducks were randomly selected from a large population for slaughter test to measure their breast muscle weight and retain blood samples; **d** the breeding of all generations was completed in Pekin duck breeding Base, Changping District, Beijing, and the performance measurement was done in spring, resulting in little difference in environmental effect between generations. Besides that, all ducks were kept in similar environment and had free access to water and feed pellets[31]. We measured the breast muscle weight of the whole population in vivo at the age of 6 weeks of each generation, and selected ducks with the heavier breast muscle weight as parents to produce the next generation. About 750 individuals in each generation were retained for breeding, of which 35 % – 40 % retention rate for female ducks and 7 % – 8 % retention rate for male ducks. The inbreeding was strictly avoided by calculating the inbreeding coefficient of each generation. The breast muscle weight (BMW) trait was estimated by breast muscle volume (BMV), breast width (BB), keel

length (KL) and breast muscle thickness (BMT) (**Fig. 2A, Supplementary Fig. S5**). The correlation equation between these traits was: BMV=BB×KL×BMT; BMW = 0.6228×BMV+17.042 [19]. BB and KL were measured by vernier calipers, while BMT was measured by ultrasound scanning technology. The correlation parameters of BMW and BMV are real and reliable numerical results based on the measured breast muscle weight of years of slaughter experiments and fitted by linear regression equation model.

In this study, we randomly selected an adult female Pekin duck from Pekin duck Z2 line to collect its blood for PacBio sequencing, and collected its breast muscle tissue for BioNano and Hi-C sequencing. Furthermore, phenotype and pedigree data from all samples were collated for subsequent analysis. All individuals are collected wing venous blood and rapidly frozen at -20 ℃. Phenol-chloroform method was used to extract blood DNA. The quality and quantity of the DNA were examined via Nanodrop and agarose gel electrophoresis. Then, Illumina HiSeq X Ten platform (Illumina HiSeq X Ten, RRID:SCR 016385) was used to sequence the paired-end sequencing libraries with an inserted fragment length of approximately 500 bp in 8 ×.

2. Reference genome assembly

We used the combined strategy of long-reads single-molecule sequencing (PacBio, RRID:SCR_017988)[54, 55], optical mapping (BioNano)[56, 57] and chromosome interaction mapping (Hi-C)[58] which improved contiguity and completeness relative to the BGI_duck_1.0[59]. We first used Canu[60] (v1.7.1) to correct and trim the subreads of PacBio with the default parameters. Then we assembled the high-quality sequence obtained in the previous step into contigs and adjusted the “correctedErrorRate” parameter to 0.05. Pilon (Pilon, RRID:SCR_014731)[61] (v1.23) was used to polish the assembled contigs twice. Then scaffolds were assembled using Irys optical mapping data. We first adopted IrysSolve (BioNano Genomics) to assembled the raw BioNano data into optical map with default parameters. Next, the runBNG pipelines[62] (v1.02) were used to construct the scaffolds based on the overlapping information between optical map and PacBio contigs.

Then we adopted Hi-C technology to anchor the scaffolds near-chromosome level. We first used bowtie2 to align the clean Hi-C raw reads to scaffolds. We totally generated ~1.06 Gb pair-end reads and ~593.5 million were uniquely mapped to the scaffolds (Supplementary Table 10). After

filtering out reads with low mapping, multiple hits, duplications, and singletons, only valid pairs were retained for subsequent analysis. After that, HiC-Pro (HiC-Pro, RRID:SCR_017643)[63] (v2.10.0) was used to construct interaction matrix for valid interaction pairs of ~371.6 million, and HiCPlotter[64] (v0.8.1) was used to draw interaction heat map. Then Juicer was used to align the clean Hi-C reads to the draft assembly, and then the extracted data were automatically generated into a nearly chromosomal length assembly using the 3d-DNA pipeline. The final draft was corrected using PBJelly (PBJelly, RRID:SCR_012091)[65].

3. Variant calling and filtering

The raw reads from Illumina sequencing were filtered before downstream analyses by removing adapter sequences, contaminated reads, and low-quality reads. Then the reads were mapped to the assembly (GCA_003850225.1) with Burrows-Wheeler alignment(BWA aln, RRID:SCR_010910)[66] (v0.7.17-r1198) using the default parameters. SAMtools (Samtools, RRID:SCR_002105)[67] (v1.13-14) software was used to convert mapping results into the BAM format and to filter the unmapped and non-unique reads. The paired reads that were mapped to the exact same position on the reference genome were identified with MarkDuplicates in Picard[68] (Picard, RRID:SCR_006525) to avoid any influence on variant detection. After the comparative evaluation of the depth and coverage of the results, we used the HaplotypeCaller program of GATK (GATK, RRID:SCR_001876)[69] (v1.90) software to call SNP and InDel to ensure the accuracy. Meanwhile, this method can avoid the interference of false positive sites in the follow-up analysis. For SNPs and InDels, we restricted the variant form to biallelic variants by setting the option of GATK to "-T SelectVariants -SelectType SNP -- RestrictTallelesto Biallelic". For the total variants, we set the GATK option '-T SelectVariants -select " AF < 1.00 " ' to limit the allele frequency. We then filtered the output by using VCFtools VCFtools (VCFtools, RRID:SCR_001235)[70] (v0.1.14). SNPs that did not meet the following criteria were excluded: (1) 3 × < mean sequencing depth (over all included individuals) < 30 ×; (2) a minor allele frequency > 0.05 and a max allele frequency < 0.99; (3) maximum missing rate < 0.1; and (4) only two alleles.

We used the CNVcaller (CNVcaller, RRID:SCR_015752) [71] software to detect copy number variations across 119 individuals, and this method also took into account the depth of reads and

pair relationship, so as to identify the copy number variation interval. We firstly specified a 1000bp sliding window and a 500 bp step to count the GC, repeat, and gap contents of each window in the reference genome to generate the reference genome database. Then we calculate the absolute number of copies per window. Thirdly, we used the “CNV.Discovery.sh” script to detect the copy number variation region of the genomes with the parameter settings “-f 0.05 -h 5 -r 0.01 -p primaryCNVR -m mergeCNVR”. Finally, we used the “Genotype.py” script to genotype the copy numbers of each sample and generate the VCF file.

4. Analysis of population genetic differences

Smartpca program of EIGENSOFT (Eigensoft, RRID:SCR_004965) [72] (v4.2) software was used for principal component analysis (PCA) of whole genome SNPs. We plotted the first two eigenvectors in two dimensions with our own R script for G1 and G4, G4 and G7, G7 and G10, as well as G1 and G10 populations, respectively.

For estimations of allele frequencies of single SNPs, we used VCFTools[70] (v0.1.14) to filter the raw sequencing data of G1 and G10 generations. The parameters are set to be “--max-missing 0.9 --maf 0.01 --min-meanDP 5 --max-meanDP 30”. After filtering, 8,433,767 reliable SNPs were obtained for allele frequency estimation. The per-SNP absolute allele frequency difference (ΔAF) between G1 generation and G10 generation was then calculated using the formula: ΔAF= abs ($RefAF_{G10}$-$RefAF_{G1}$). We next binned SNPs by ΔAF in steps of 0.05 (i.e. ΔAF=0–0.05, 0.05–0.10, etc. until 0.95-1.00) and intersected these binned SNPs with coding exons, introns and UTRs.

5. Genome-wide association analysis of traits

A genome-wide association analysis (GWAS) was performed using a mixed linear model (MLM) of EMMAX[73] program using genome-wide SNP data and breast muscle weight of 119 individuals from the re-sequenced population. The analysis model was:

$y = Xb + G\mathrm{a} + e.$

Where, y is the phenotypic value (the breast muscle weight of per duck), X is the matrix corresponding to the fixed effect, and b is the fixed effect size. Fixed effects include sex effects. G is the genetic matrix corresponding to the population kinship, and e is the random residual. PCA was performed based on all SNPs and the top three components were set as fixed effects in the mixed model to correct for population stratification. We defined Bonferroni correction threshold

of 0.01/N (-$\log_{10} P$ = 8.94) to identify the significant loci of the GWAS results, where N was the number of whole-genome SNPs.

For the identified associated genes, we also referred to the Pekin duck Panoramic transcription map (transcriptome data of all tissues in the three development stages) established by our previous study[45] to check whether these genes were expressed in the development stage of breast weight muscle, so as to further confirm that them did participate in the regulation of breast muscle development.

6. Identification of selected regions

We used the VCFtools [70](v0.1.14) software to calculate *F*st between G1 and G10 generations by selecting parameters of 10 kb windows with a 5 kb step size. We used selscan software[74](v1.2.0a) to calculate the XP-EHH values of G1 and G10 groups with the same parameter settings of 10 kb sliding windows and 5 kb step size, in which the G1 generation was taken as the reference group and the G10 generation as the query group. The overlapping regions of two windows with statistical values above the top 1 % of the quantile would be selected as preliminary candidate regions.

7. Linkage disequilibrium analysis

In addition, to further narrow the candidate interval, we also used the Haploview (Haploview, RRID:SCR_003076)[75] to analyze the linkage disequilibrium of the candidate region (Related to **Fig. 4**). We adopted the square correlation coefficient (r^2) as the coefficient to measure the linkage disequilibrium between the leader SNP and the surrounding SNPs. The parameters settings were "--ld-window 99999 --ld-window- kb 1000 --ld-window-r2 0 --r2". Then we integrated and plotted the LD data with GWAS results. (Related to **Supplementary Fig. S4**).

8. Identification of selected CNVRs

Based our new assembly, we used CNV caller[71], to identify genome-wide CNVs and CNVRs. To avoid false positives, two parameters, silhouette coefficient (silhouette coefficient > 0.7) and minor allele frequency (MAF>0.05), were adopted to filter the CNVRs obtained, and then we got 81 CNVRs with high credibility and accuracy on autosomes. Subsequently, we used the relative frequency difference (RFD)[76], to detect the copy number variations occurred on the Pekin duck

genome during population differentiation. After that, we adopted the topmost 5% RFD value (absolute RFD value > 4.1) as the threshold to screen the potentially selected CNVRs.

Data Availability

The assembly and annotation of Pekin duck has been deposited in GenBank under the Bioproject accession code PRJNA496533 (accession No. RHJV01000000).

Figure Legends

Fig. 1 Overview of the assembly quality and characteristics of the Pekin duck genome.

A Circular diagram depicting the characteristics of the GCA_003850225.1 assembly. The tracks from the outer to inner circles represent the following: chromosomes, gene density (window size of 200 kb), SNP density (window size of 200 kb), TE density (window size of 200 kb), CNV density (window size of 3 Mb) and GC content (%) (window size of 200 kb). **B, C** Tree maps of fragmentation differences between long-read (GCA_003850225.1) and short-read (BGI_duk1.0) Pekin duck genome assemblies. The size of each rectangle of each chromosome is scaled to that of the contig sequence. The larger and fewer the internal boxes are, the more contiguous the contigs. **D** Comparison of the sequence contig length distribution between long-read (GCA_003850225.1) and short-read (BGI_duk_1.0) Pekin duck genome assemblies. **E** Comparison of mapping rates when the Pekin duck population (119 birds from 10 generations) whole-genome resequencing data were mapped to GCA_003850225.1 and the short-read genome assembly (BGI_duk_1.0). A two-tailed paired t test was used for statistical assessment. ****P* < 0.001.

Fig. 2 Phenotypic and population genetic structure variation over ten generations.

A Measurement of duck breast muscle volume (BMV) in vivo at 6 weeks of age. The three measured values were: ① breast breadth (BB), ② keel length (KL), and ③ breast muscle thickness (BMT). The weight of breast muscle was calculated based on the following formulas: BMV = BB×KL×BMT, and BMW = 0.6228×BMV+17.042. **B** Principal component analysis (PCA) of ten generations. The red circles represent first-generation (G1) individuals, the yellow circles represent fourth-generation (G4) individuals, the green circles represent seventh-generation (G7) individuals, and the blue circles represent tenth-generation (G10) individuals. **C** Changes in breast muscle weight in the Pekin duck Z2 line over ten generations.

Fig. 3 Δallele frequency analyses. The majority of SNPs showed low Δ*AF* values between the first and tenth generation ducks. The black line indicates the number of SNPs in nonoverlapping Δ*AF* bins (left y axis). The colored lines denote the M values (log2-fold changes) of the relative frequencies of SNPs in coding regions (yellow), UTRs (green), and introns (blue), according to Δ*AF* bins (right y-axis).

Fig. 4 Overlapping selection signals in the genomes of the first and tenth generations. A Manhattan plot of selected regions between the first generation (G1) and the tenth generation (G10). Pairwise fixation index values (*F*st) are calculated in 10-kb sliding windows and 5-kb steps. The significance threshold for *F*st is 0.103 (1%). **B** Manhattan plot of selected regions between the first generation (G1) and the tenth generation (G10). Cross-population extended haplotype homozygosity (XP-EHH) values were calculated in 10-kb sliding windows and 5-kb steps. The significance thresholds for XP-EHH were 1.475 and -1.480 (1%). **C** Manhattan plots for a GWAS of breast muscle weight. The gray horizontal dashed lines indicate the Bonferroni-corrected significance threshold of the GWAS ($-\log_{10} P = 8.94$), and the selection signals are indicated with a gray background. **D** Allele frequency trajectories of 22 SNPs. **E** The variation trend of genetic diversity in selected regions among generations (Chr3:0.18-0.44 Mb; Chr16:3.40-3.50 Mb). The indicated *P* values are based on one-way ANOVA. *** indicates $P < 0.001$, ** indicates $P < 0.01$, * indicates $P < 0.05$, and ns indicates that the *P* value was not significant. **F** Regions containing loci associated with breast muscle weight ranging from 0.18 to 0.44 Mb along chromosome 3 and 3.40 to 3.50 Mb along chromosome 16. All genotyped SNPs are color coded according to their pairwise LD with the leader SNP (Chr3:416692; Chr16:3467244) calculated by comparing the first generation (G1) and tenth-generation (G10) populations. SNPs are colored based on the strength of the LD values (r^2 values) considering the most strongly associated SNP and the other SNPs in the region. **G** The blue line diagrams refer to the fixation indexes (*F*st) on selected regions (Chr3:0.18-0.44 Mb; Chr16:3.40-3.50 Mb) between G1 and G10. *F*st values are calculated in 10-kb sliding windows in 5-kb steps. The selection signals that overlapped with characterized GWAS loci are indicated with a gray background. The green line diagrams refer to XP-EHH for selected regions (Chr3:0.18-0.44 Mb; Chr16:3.40-3.50 Mb) between G1 and G10.

XP-EHH values are calculated in 10-kb sliding windows in 5-kb steps. The selection signals that overlapped with characterized GWAS loci are indicated with a gray-blue background. **H** Schematic diagram showing the genes distributed within the candidate regions (Chr3:0.34-0.43 Mb and Chr16:3.46-3.49 Mb). **I** Associations between genotypes of two leader SNPs in candidate regions and breast muscle weight. Box plots indicate the median (centerline), 25th-75th percentiles (limits), and minimum and maximum values (whiskers). The indicated *P* values are based on one-way ANOVA. *** indicates $P < 0.001$, ** indicates $P < 0.01$, * indicates $P < 0.05$, and ns indicates that the *P* value was not significant.

Fig. 5 Genome-wide screening of selected copy number variations (CNVs) between the first and tenth generations. **A** The relative frequency difference (RFD) between the first generation (G1) and the tenth generation (G10) is plotted against the position on each of the autosomes. The two horizontal dashed lines indicate the genome-wide thresholds of selection signals, which showed the highest absolute RFD value of 5% (> 4.1). **B** Copy number variant form of selected CNVs. CNV1 (Chr1:200.83-201.27 Mb) and CNV2 (Chr2:123.14-123.16 Mb) mainly consisted of multiple copies, and CNV3 (Chr4:54.81-54.82 Mb) was a deletion. **C** Schematic diagram showing the genes distributed within the candidate regions. **D** Frequency changes in candidate CNVs over 10 generations.

Table 1 Assembly statistics of the GCA_003850225.1 genome.

Additional Files

Supplemental Figure S1. The pipeline for multi-level chromosome assembly. Canu was used for constructing initial contigs. Then polishing was performed with Pilon using PacBio-only long reads. Hybrid scaffolding of the PacBio-corrected contigs and the BioNano-based consensus map was performed using the hybrid scaffolding module within runBNG software. The Hi-C sequencing data were first aligned to the assembled contigs/scaffolds using the Bowtie end-to-end algorithm, and then the assembled scaffolds were clustered, ordered, and directed into chromosome level using 3d-DNA. The final draft was corrected using PBJelly.

Supplemental Figure S2. Hi–C interactions among 29 chromosomes with a 40-kb resolution.

Supplemental Figure S3. BUSCO completeness assessment for the new assembly. In summary, it covered 93.3% (7780/8338) of complete BUSCO genes and 1.3% (111/8338) of fragmented BUSCO genes.

Supplemental Figure S4. Generational average phenotypic values of body weight (BW) and breast muscle volume (BMV).

Supplemental Figure S5. Distribution of *F*st and XP-EHH of 10-kb windows size for whole-genome-wide variants between G1 and G10 generation. Bins of *F*st and XP-EHH are presented along the x axes. μ, mean; δ, standard deviation.

Supplemental Figure S6. Venn diagram showing the number of unique and overlapping regions from Top 1% of *F*st and XP-EHH. Numbers represent the counts of candidate regions identified by each strategy.

Supplemental Figure S7. The variation of allele frequency of putative regions over ten generations (correspond to Figure 2). (A) and (B) Red and green lines indicate the frequency variation of representative loci in selective regions of *F*st and XP-EHH, respectively (corresponding to a 1% significance level of *F*st in 10-kb sliding windows and a 1% significance level of XP-EHH in 10-kb sliding windows; and each line represents the top SNP of a sliding window). **(C)** Blue lines indicate the frequency variation of 22 SNPs reached the Bonferroni significance threshold of the GWAS ($-\log_{10} P > 8.94$).

Supplementary Figure S8. The variation trend of genetic diversity in potential selective regions among generations. This figure shows the trend of genetic diversity in the common

selection signatures obtained by *F*st and XP-EHH tests across generations. The indicated *P* values are based on one-way ANOVA. ** indicates $P < 0.01$, * indicates $P < 0.05$ and ns indicates that *P* value was not significant.

Supplemental Figure S9. Fixation index (*F*st) and cross-population extended haplotype homozygosity (XP-EHH) for selection regions (Chr3:0.18-0.44 Mb; Chr16:3.40-3.50 Mb) between G1 and G10. Each blue dot represents a locus.

Supplemental Figure S10. Linkage disequilibrium (LD) in two candidate regions of 10 generations. Each diamond contains a level of LD (r^2) between all SNP pairs.

Supplemental Figure S11. SNPs distribution in candidate regions.

Supplemental Figure S12. Expression levels of the *UTP25* and *FBRSL1* genes in different tissues of Pekin duck. (A) *UTP25* expression levels in Pekin duck tissues. **(B)** *FBRSL1* expression levels in Pekin duck tissues. The expression data were obtained from a global gene expression database for ducks generated from transcriptome analyses. The database lists the expression levels of all genes in breast muscle, skin, liver, fat (abdominal fat), brain, heart, kidney, lung, spleen, sternum and shank tissues at different developmental periods in Pekin ducks.

Supplemental Figure S13. Expression levels of the *EXOC4* and *TRPA1* genes in different tissues of Pekin duck. (A) *EXOC4* expression levels in Pekin duck tissues. **(B)** *TRPA1* expression levels in Pekin duck tissues. The expression data were obtained from a global gene expression database for ducks generated from transcriptome analyses. The database lists the expression levels of all genes in breast muscle, skin, liver, fat (abdominal fat), brain, heart, kidney, lung, spleen, sternum and shank tissues at different developmental periods in Pekin ducks.

Supplemental Table S1. Summary of sequencing data.

Supplemental Table S2. Summary of Hi-C reads mapping results.

Supplemental Table S3. Comparison of genome assemblies between the GCA_003850225.1 and BGI_duck_1.0.

Supplemental Table S4. Number of ducks used for 10 generations genome re-sequencing and mapping summary.

Supplemental Table S5. Genomic SNP statistics.

Supplemental Table S6. Distributions of SNP counts in the different delta allele frequency bins for coding sequences, introns and UTRs.

Supplemental Table S7. Overlapping regions between the analysis results of *F*st and XP-EHH. Pairwise fixation index values (*F*st) and cross-population extended haplotype homozygosity (XP-EHH) values are calculated in 10-kb windows sliding in 5-kb steps. The top 1% of the overlap regions between the analysis results of *F*st and XP-EHH were considered as candidate regions.

Supplemental Table S8. Summary of putative regions on the genome. These regions sum to 2.06 Mb and ~2.14 Mb were annotated into 65 genes.

Supplemental Table S9. The significant SNPs of GWAS analyses. We performed GWAS analyses of breast muscle weight. The Bonferroni significance threshold of the GWAS was $-\log_{10} P > 8.94$. There were 22 SNPs that passed the Bonferroni significance threshold. These SNPs are located in non-coding sequences.

Supplemental Table S10. Putative CNVRs under artificial selection.

Supplemental Table S11. The copy number variation genotypes of three candidate CNVRs in each individual.

Abbreviations

BB: breast breadth; BMT: breast muscle thickness; BMV: breast muscle volume; BMW: breast muscle weight; bp: base pairs; BUSCO: Benchmarking Universal Single-Copy Orthologs; BWA: BurrowsWheeler Aligner; CNV: Copy number variation; *F*st: Fixation index; g: gram; G:generation; CNVR: copy number variation regions; Gb: gigabase pairs; GC: guanine-cytosine; GWAS: genome-wide association study; Hi-C: High-throughput Chromosome Conformation Capture; kb: kilobase pairs; KL: keel length; PCA: principle component analysis; LD: linkage disequilibrium; Mb: megabase pairs; MLM: mixed linear model; NCBI: National Center for Biotechnology Information; PacBio: Pacific Biosciences; RFD: relative frequency difference; SNP: single-nucleotide polymorphism; TE: transposable element; UTR: untranslated regions; XP-EHH: population extended haplotype homozygosity; ΔAF: allele frequency difference.

Competing interests

The authors have declared that no competing interests exist.

Ethics statement

All animals used in the study were treated following the guidelines for the experimental animals established by the Council of China Animal Welfare. Protocols of the experiments were approved by the Science Research Department of the Institute of Animal Sciences, Chinese Academy of Agricultural Sciences (CAAS) (Beijing, China).

Funding

This work was supported by grants from the National Natural Science Foundation of China (31972523), the Young Top-notch Talent Project of the National Ten Thousand Talent Program, the China Agriculture Research System of MOF and MARA (CARS-42-5) and the CAAS Innovation Team Project (ASTIP-IAS-9, CAAS-ZDRW202104).

Authors' contributions

Z.Zhou. and S.H. conceived the project and designed the research. managed the project. S.H., Z.Zhou., Z.G., J.Hu., M.Xie., W.Huang., Y.Zhang., and Q.Zhang. constructed the population. M.Xie., J.T., W.Huang., J.Hu., Y.Zhang., Z.G., G.Xing, W.F., Y.Xu, S.Liang. and D.Liu. collected the phenotype data. Y. Jiang., Z.Liu., M.Li., H.C., Z.Zhou. performed the genome assembly. S.Y., Z.Zhou., Z.Liu., D.Zhou. and P.Hua. performed bioinformatics analysis. S.Y., and Z.Zhou. wrote the manuscript.

Acknowledgments

We are grateful to all the members who involved in sample collection and processing.

References

1. Carneiro M, Rubin CJ, Di Palma F, Albert FW, Alfoldi J, Barrio AM, et al. Rabbit genome analysis reveals a polygenic basis for phenotypic change during domestication. Science. 2014;345(6200):1074-9. doi: 10.1126/science.1253714.

2. Li M, Tian S, Jin L, Zhou G, Li Y, Zhang Y, et al. Genomic analyses identify distinct patterns of selection in domesticated pigs and Tibetan wild boars. Nat Genet. 2013;45(12):1431-8. doi: 10.1038/ng.2811.

3. Rubin C-J, Zody MC, Eriksson J, Meadows JRS, Sherwood E, Webster MT, et al. Whole-genome resequencing reveals loci under selection during chicken domestication. Nature. 2010;464(7288):587-91. doi: 10.1038/nature08832.

4. Enfield FD, Comstock RE, Braskerud O. Selection for Pupa Weight in TRIBOLIUM CASTANEUM. I. Parameters in Base Populations Genetics Selection Evolution. 1966;54(1):523-33.

5. Marks HL. Long-term selection for body weight in Japanese quail under different environments. Poult Sci. 1996;75(10):1198-203. Epub 1996/10/01. doi: 10.3382/ps.0751198.

6. Aggrey SE, Ankra-Badu GA, Marks HL. Effect of long-term divergent selection on growth characteristics in Japanese quail. Poult Sci. 2003;82(4):538-42. Epub 2003/04/25. doi: 10.1093/ps/82.4.538.

7. Mott IW, Ivarie R. cDNA array analysis of Japanese quail lines divergently selected for four-week body weight. Poult Sci. 2004;83(9):1524-9. Epub 2004/09/24. doi: 10.1093/ps/83.9.1524.

8. Moe HH, Shimogiri T, Kamihiraguma W, Isobe H, Kawabe K, Okamoto S, et al. Analysis of polymorphisms in the insulin-like growth factor 1 receptor (IGF1R) gene from Japanese quail selected for body weight. Anim Genet. 2007;38(6):659-61. Epub 2007/10/25. doi: 10.1111/j.1365-2052.2007.01653.x.

9. Siegel PB. Selection for Body Weight at Eight Weeks of Age: 1. Short term Response and Heritabilities1. Poult Sci. 1962;41(3):954-62. doi: 10.3382/ps.0410954.

10. Johansson AM, Pettersson ME, Siegel PB, Carlborg O. Genome-wide effects of long-term divergent selection. PLoS Genet. 2010;6(11):e1001188. Epub 2010/11/17. doi: 10.1371/journal.pgen.1001188.

11. Dunnington EA, Siegel PB. Long-Term Divergent Selection for Eight-Week Body Weight in White Plymouth Rock Chickens. Poult Sci. 1996;75(10):1168-79. doi: 10.3382/ps.0751168.

12. Lillie M, Sheng ZY, Honaker CF, Andersson L, Siegel PB, Carlborg Ö. Genomic signatures of 60 years of bidirectional selection for 8-week body weight in chickens. Poult Sci. 2018;97(3):781-90. doi: 10.3382/ps/pex383.

13. Mette L, F HC, B SP, Örjan C. Bidirectional Selection for Body Weight on Standing Genetic Variation in a Chicken Model. G3. 2019;9(4). doi: 10.1534/g3.119.40003810.25387/g3.7674281.

14. Wu X, Zhang Q, Xu S, Jin P, Luan P, Li Y, et al. Differential expression of six chicken genes associated with fatness traits in a divergently selected broiler population. Mol Cell Probes. 2016;30(1):1-5. Epub 2015/12/30. doi: 10.1016/j.mcp.2015.12.003

15. Wang HB, Li H, Wang QG, Zhang XY, Wang SZ, Wang YX, et al. Profiling of chicken adipose tissue gene expression by genome array. BMC Genom. 2007;8:193. Epub 2007/06/28. doi: 10.1186/1471-2164-8-193.

16. Zhang H, Hu X, Wang Z, Zhang Y, Wang S, Wang N, et al. Selection signature analysis implicates the PC1/PCSK1 region for chicken abdominal fat content. PloS One. 2012;7(7):e40736-e. Epub 2012/07/11. doi: 10.1371/journal.pone.0040736.

17. Zhang H, Wang S-Z, Wang Z-P, Da Y, Wang N, Hu X-X, et al. A genome-wide scan of selective sweeps in two broiler chicken lines divergently selected for abdominal fat content. BMC Genom. 2012;13. doi: 10.1186/1471-2164-13-704.

18. Yang Z-M, Wang W, Du Z-Q, Cheng B, Wang Y, Yao J, et al. Expression Profiling of Preadipocyte MicroRNAs by Deep Sequencing on Chicken Lines Divergently Selected for Abdominal Fatness. PLoS One. 2015;10(2). doi: 10.1371/journal.pone.0117843.

19. Li X, Yang J, Shen M, Xie XL, Liu GJ, Xu YX, et al. Whole-genome resequencing of wild and domestic sheep identifies genes associated with morphological and agronomic traits. Nat Commun. 2020;11(1):2815. doi: 10.1038/s41467-020-16485-1.

20. Zheng Z, Wang X, Li M, Li Y, Yang Z, Wang X, et al. The origin of domestication genes in goats. Sci Adv. 2020;6:eaaz5216. doi: 10.1126/sciadv.aaz5216.

21. Dong Y, Zhang X, Xie M, Arefnezhad B, Wang Z, Wang W, et al. Reference genome of wild goat (capra aegagrus) and sequencing of goat breeds provide insight into genic basis of goat domestication. BMC Genom. 2015;16:431. Epub 2015/06/06. doi: 10.1186/s12864-015-1606-1.

22. Ai H, Fang X, Yang B, Huang Z, Chen H, Mao L, et al. Adaptation and possible ancient interspecies introgression in pigs identified by whole-genome sequencing. Nat Genet. 2015;47(3):217-25. Epub 2015/01/27. doi: 10.1038/ng.3199.

23. Li J, Zhang J, Liu J, Zhou Y, Cai C, Xu L, et al. A new duck genome reveals conserved and convergently evolved chromosome architectures of birds and mammals. GigaScience. 2021;10(1):giaa142. doi: 10.1093/gigascience/giaa142.

24. Zhang L, Hu J, Han X, Li J, Gao Y, Richards CM, et al. A high-quality apple genome assembly reveals the association of a retrotransposon and red fruit colour. Nat Commun. 2019;10(1):1494. doi: 10.1038/s41467-019-09518-x.

25. Peace CP. DNA-informed breeding of rosaceous crops: promises, progress and prospects. Hortic Res. 2017;4:17006. doi: 10.1038/hortres.2017.6.

26. Bickhart DM, Rosen BD, Koren S, Sayre BL, Hastie AR, Chan S, et al. Single-molecule sequencing and chromatin conformation capture enable de novo reference assembly of the domestic goat genome. Nat Genet. 2017;49(4):643-50. Epub 2017/03/07. doi: 10.1038/ng.3802.

27. Zhu F, Yin Z-T, Wang Z, Smith J, Zhang F, Martin F, et al. Three chromosome-level duck genome assemblies provide insights into genomic variation during domestication. Nat Commun. 2021;12(1). doi: 10.1038/s41467-021-26272-1.

28. Li M, Sun C, Xu N, Bian P, Tian X, Wang X, et al. De Novo Assembly of 20 Chicken Genomes Reveals the Undetectable Phenomenon for Thousands of Core Genes on Microchromosomes and

Subtelomeric Regions. Mol Biol Evol. 2022;39(4). doi: 10.1093/molbev/msac066.

29. Wang K, Hu H, Tian Y, Li J, Scheben A, Zhang C, et al. The chicken pan-genome reveals gene content variation and a promoter region deletion in IGF2BP1 affecting body size. Mol Biol Evol. 2021. Epub 2021/07/31. doi: 10.1093/molbev/msab231.

30. Simao FA, Waterhouse RM, Ioannidis P, Kriventseva EV, Zdobnov EM. BUSCO: assessing genome assembly and annotation completeness with single-copy orthologs. Bioinformatics. 2015;31(19):3210-2. doi: 10.1093/bioinformatics/btv351.

31. Xu Y, Hu J, Zhang Y, Guo Z, Huang W, Xie M, et al. Selection response and estimation of the genetic parameters for multidimensional measured breast meat yield related traits in a long-term breeding Pekin duck line. Asian-australas J Anim Sci. 2018;31(10):1575-80. Epub 2018/04/13. doi: 10.5713/ajas.17.0837.

32. Reich D, Price AL, Patterson N. Principal component analysis of genetic data. Nat Genet. 2008;40(5):491-2. doi: 10.1038/ng0508-491.

33. Pritchard JK, Pickrell JK, Coop G. The genetics of human adaptation: hard sweeps, soft sweeps, and polygenic adaptation. Curr Biol. 2010;20(4):R208-15. Epub 2010/02/25. doi: 10.1016/j.cub.2009.11.055.

34. Wright S. The genetical strucyure of populations. Annals of Eugenics. 1949;15(1):323-54. doi: 10.1111/j.1469-1809.1949.tb02451.x.

35. Sabeti PC, Varilly P, Fry B, Lohmueller J, Hostetter E, Cotsapas C, et al. Genome-wide detection and characterization of positive selection in human populations. Nature. 2007;449(7164):913-8. Epub 2007/10/19. doi: 10.1038/nature06250.

36. Cooper GM, Nickerson DA, Eichler EE. Mutational and selective effects on copy-number variants in the human genome. Nat Genet. 2007;39(7 Suppl):S22-9. Epub 2007/09/05. doi: 10.1038/ng2054.

37. Bickhart DM, Hou Y, Schroeder SG, Alkan C, Cardone MF, Matukumalli LK, et al. Copy number variation of individual cattle genomes using next-generation sequencing. Genome Res. 2012;22(4):778-90. doi: 10.1101/gr.133967.111.

38. Weischenfeldt J, Symmons O, Spitz F, Korbel JO. Phenotypic impact of genomic structural variation: insights from and for human disease. Nat Rev Genet. 2013;14(2):125-38. doi: 10.1038/nrg3373.

39. Xu Y, Shi T, Cai H, Zhou Y, Lan X, Zhang C, et al. Associations of MYH3 gene copy number variations with transcriptional expression and growth traits in Chinese cattle. Gene. 2014;535(2):106-11. doi: 10.1016/j.gene.2013.11.057.

40. Sniekers S, Stringer S, Watanabe K, Jansen PR, Coleman JRI, Krapohl E, et al. Genome-wide association meta-analysis of 78,308 individuals identifies new loci and genes influencing human intelligence. Nat Genet. 2017;49(7):1107-12. Epub 2017/05/23. doi: 10.1038/ng.3869.

41. Nozawa K, Kawabata-Shoda E, Doihara H, Kojima R, Okada H, Mochizuki S, et al. TRPA1 regulates gastrointestinal motility through serotonin release from enterochromaffin cells. Proc Natl Acad Sci USA.. 2009;106(9):3408-13. doi: 10.1073/pnas.0805323106.

42. Van Laere A-S, Nguyen M, Braunschweig M, Carine N, Collette C, Moreau L, et al. A regulatory mutation in IGF2 causes a major QTL effect on muscle growth in the pig. Nature. 2003;425:832-6. doi: 10.1038/nature02064.

43. Clop A, Marcq F, Takeda H, Pirottin D, Tordoir X, Bibe B, et al. A mutation creating a potential illegitimate microRNA target site in the myostatin gene affects muscularity in sheep. Nature Genetics. 2006;38(7):813-8. Epub 2006/06/06. doi: 10.1038/ng1810.

44. Karim L, Takeda H, Lin L, Druet T, Arias JA, Baurain D, et al. Variants modulating the expression of a chromosome domain encompassing PLAG1 influence bovine stature. Nat Genet. 2011;43(5):405-13. doi: 10.1038/ng.814.

45. Zhou Z, Li M, Cheng H, Fan W, Yuan Z, Gao Q, et al. An intercross population study reveals genes associated with body size and plumage color in ducks. Nat Commun. 2018;9(1):2648. Epub 2018/07/19. doi: 10.1038/s41467-018-04868-4.

46. Zan Y, Sheng Z, Lillie M, Ronnegard L, Honaker CF, Siegel PB, et al. Artificial Selection Response due to Polygenic Adaptation from a Multilocus, Multiallelic Genetic Architecture. Mol Biol Evol. 2017;34(10):2678-89. doi: 10.1093/molbev/msx194

47. Zhang H, Liang Q, Wang N, Wang Q, Leng L, Mao J, et al. Microevolutionary dynamics of chicken genomes under divergent selection for adiposity. iScience. 2020;23(6):101193. doi: 10.1016/j.isci.2020.101193.

48. Carroll SB. Evo-devo and an expanding evolutionary synthesis: a genetic theory of morphological evolution. Cell. 2008;134(1):25-36. doi: 10.1016/j.cell.2008.06.030.

49. Stern DL, Orgogozo V. The loci of evolution: how predictable is genetic evolution? Evolution. 2008;62(9):2155-77. doi: 10.1111/j.1558-5646.2008.00450.x.

50. Wittkopp PJ, Kalay G. Cis-regulatory elements: molecular mechanisms and evolutionary processes underlying divergence. Nat Rev Genet. 2011;13(1):59-69. doi: 10.1038/nrg3095.

51. Monroe JG, Srikant T, Carbonell-Bejerano P, Becker C, Lensink M, Exposito-Alonso M, et al. Mutation bias reflects natural selection in Arabidopsis thaliana. Nature. 2022. doi: 10.1038/s41586-021-04269-6.

52. Nei M, Suzuki Y, Nozawa M. The neutral theory of molecular evolution in the genomic era. Annu Rev Genom Hum G 2010;11:265-89. doi: 10.1146/annurev-genom-082908-150129.

53. Dunnington EA, Honaker CF, McGilliard ML, Siegel PB. Phenotypic responses of chickens to long-term, bidirectional selection for juvenile body weight--historical perspective. Poult Sci. 2013;92(7):1724-34. doi: 10.3382/ps.2013-03069.

54. Eid J, Fehr A, Gray J, Luong K, Lyle J, Otto G, et al. Real-Time DNA sequencing from single polymerase molecules. Science. 2009;323(5910):133-8. doi: 10.1126/science.1162986.

55. Gordon D, Huddleston J, Chaisson MJ, Hill CM, Kronenberg ZN, Munson KM, et al. Long-read sequence assembly of the gorilla genome. Science. 2016;352(6281):aae0344. doi: 10.1126/science.aae0344.

56. Schwartz DC, Li X, Hernandez LI, Ramnarain SP, Huff EJ, Wang YK. Ordered restriction maps of Saccharomyces cerevisiae chromosomes constructed by optical mapping. Science.

1993;262(5130):110-4.
57. Hastie AR, Dong L, Smith A, Finklestein J, Lam ET, Huo N, et al. Rapid genome mapping in nanochannel arrays for highly complete and accurate de novo sequence assembly of the complex Aegilops tauschii genome. PLoS One. 2013;8(2):e55864. doi: 10.1371/journal.pone.0055864.
58. Lieberman-Aiden E, van Berkum NL, Williams L, Imakaev M, Ragoczy T, Telling A, et al. Comprehensive mapping of long-range interactions reveals folding principles of the human genome. Science. 2009;326(5950):289-93. doi: 10.1126/science.1181369.
59. Huang Y, Li Y, Burt DW, Chen H, Zhang Y, Qian W, et al. The duck genome and transcriptome provide insight into an avian influenza virus reservoir species. Nat Genet. 2013;45(7):776-83. Epub 2013/06/12. doi: 10.1038/ng.2657.
60. Koren S, Walenz BP, Berlin K, Miller JR, Bergman NH, Phillippy AM. Canu: scalable and accurate long-read assembly via adaptivek-mer weighting and repeat separation. Genome Res. 2017;27(5):722-36. doi: 10.1101/gr.215087.116.
61. Walker BJ, Abeel T, Shea T, Priest M, Abouelliel A, Sakthikumar S, et al. Pilon: an integrated tool for comprehensive microbial variant detection and genome assembly improvement. PLoS One. 2014;9(11):e112963. doi: 10.1371/journal.pone.0112963.
62. Yuan Y, Bayer PE, Lee H-T, Edwards D. runBNG: a software package for BioNano genomic analysis on the command line. Bioinformatics. 2017;33(19):3107-9. doi: 10.1093/bioinformatics/btx366.
63. Servant N, Varoquaux N, Lajoie BR, Viara E, Chen CJ, Vert JP, et al. HiC-Pro: an optimized and flexible pipeline for Hi-C data processing. Genome Biol. 2015;16:259. doi: 10.1186/s13059-015-0831-x.
64. Akdemir KC, Chin L. HiCPlotter integrates genomic data with interaction matrices. Genome Biol. 2015;16:198. doi: 10.1186/s13059-015-0767-1.
65. English AC, Richards S, Han Y, Wang M, Vee V, Qu J, et al. Mind the gap: upgrading genomes with Pacific Biosciences RS long-read sequencing technology. PLoS One. 2012;7(11):e47768. doi: 10.1371/journal.pone.0047768.
66. Li H, Durbin R. Fast and accurate short read alignment with Burrows-Wheeler transform. Bioinformatics. 2009;25(14):1754-60. doi: 10.1093/bioinformatics/btp324.
67. Li H, Handsaker B, Wysoker A, Fennell T, Ruan J, Homer N, et al. The sequence alignment/map format and SAMtools. Bioinformatics. 2009;25(16):2078-9. doi: 10.1093/bioinformatics/btp352.
68. https://github.com/broadinstitute/picard.
69. McKenna A, Hanna M, Banks E, Sivachenko A, Cibulskis K, Kernytsky A, et al. The Genome Analysis Toolkit: a MapReduce framework for analyzing next-generation DNA sequencing data. Genome Res. 2010;20(9):1297-303. doi: 10.1101/gr.107524.110.
70. Danecek P, Auton A, Abecasis G, Albers CA, Banks E, DePristo MA, et al. The variant call format and VCFtools. Bioinformatics. 2011;27(15):2156-8. doi: 10.1093/bioinformatics/btr330.
71. Xihong W, Zhuqing Z, Yudong C, Ting C, Chao L, Weiwei F, et al. CNVcaller: highly efficient and widely applicable software for detecting copy number variations in large populations. Gigascience.

2017;(12):1-12. doi: 10.1093/gigascience/gix115.

72. Price AL, Patterson NJ, Plenge RM, Weinblatt ME, Shadick NA, Reich D. Principal components analysis corrects for stratification in genome-wide association studies. Nat Genet. 2006;38(8):904-9. doi: 10.1038/ng1847.

73. Kang HM, Sul JH, Service SK, Zaitlen NA, Kong S-Y, Freimer NB, et al. Variance component model to account for sample structure in genome-wide association studies. Nat Genet. 2010;42(4):348-54. doi: 10.1038/ng.548.

74. Szpiech ZA, Hernandez RD. selscan: An Efficient Multithreaded Program to Perform EHH-Based Scans for Positive Selection. Mol Biol Evol. 2014;31(10):2824-7. doi: 10.1093/molbev/msu211.

75. Barrett JC, Fry B, Maller J, Daly MJ. Haploview: analysis and visualization of LD and haplotype maps. Bioinformatics. 2005;21(2):263-5. doi: 10.1093/bioinformatics/bth457.

76. Zhou Z, Jiang Y, Wang Z, Gou Z, Lyu J, Li W, et al. Resequencing 302 wild and cultivated accessions identifies genes related to domestication and improvement in soybean. Nat Biotechnol. 2015;33(4):408-14. doi: 10.1038/nbt.3096.

Click here to access/download;Figure;Figure1.tif

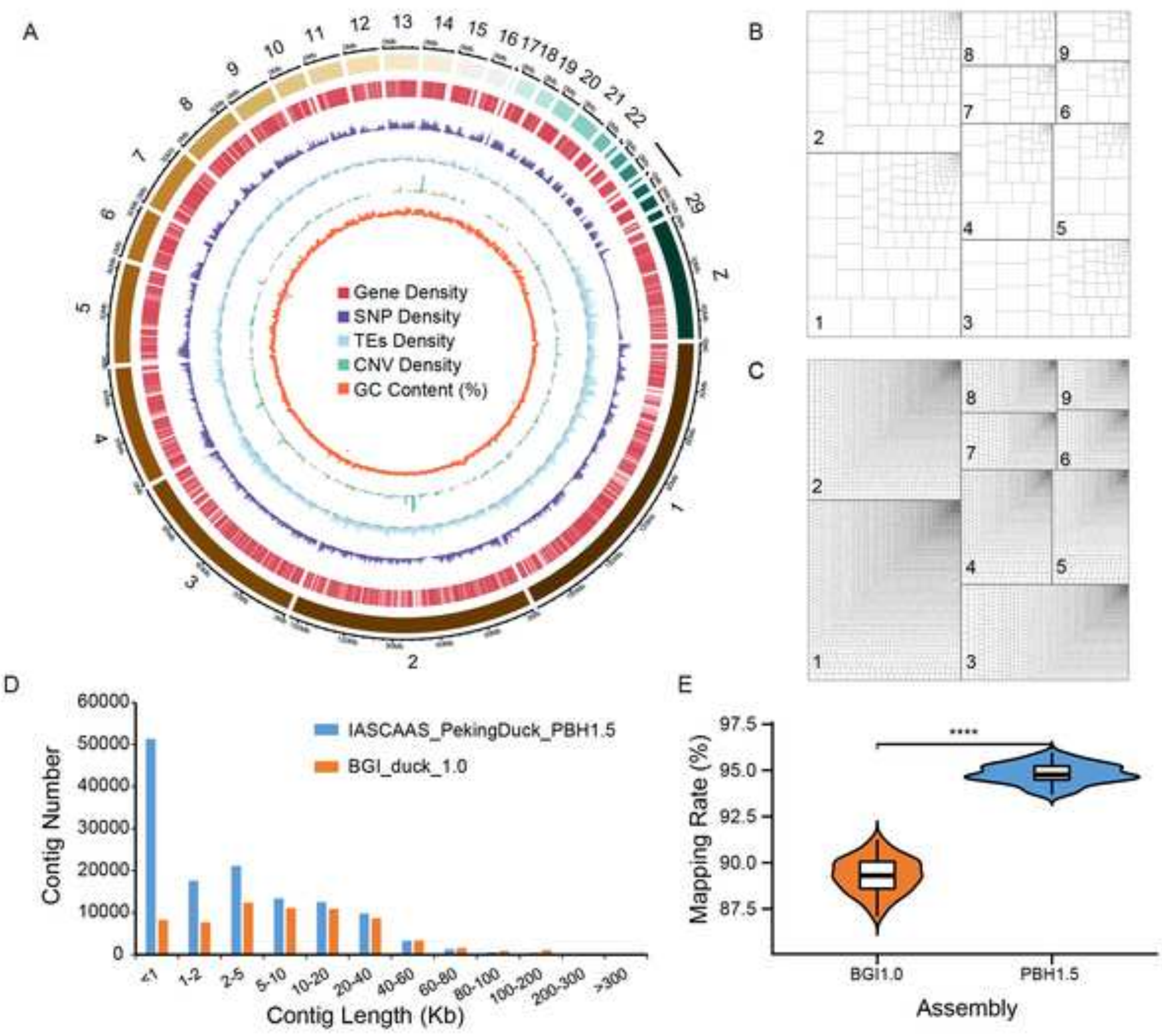

Figure

Click here to access/download;Figure;Figure2.tif

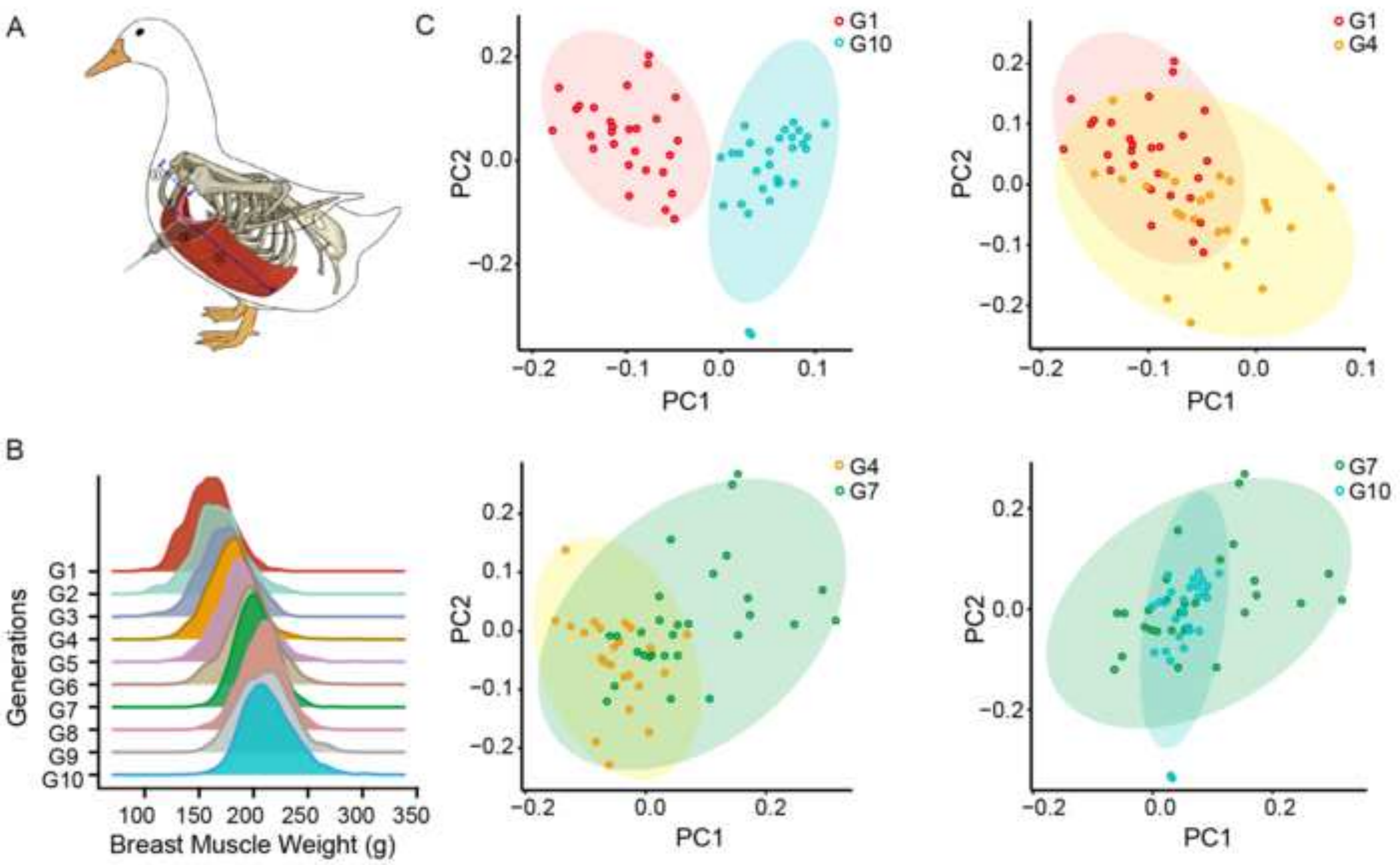

Figure
Click here to access/download;Figure;Figure3.tif

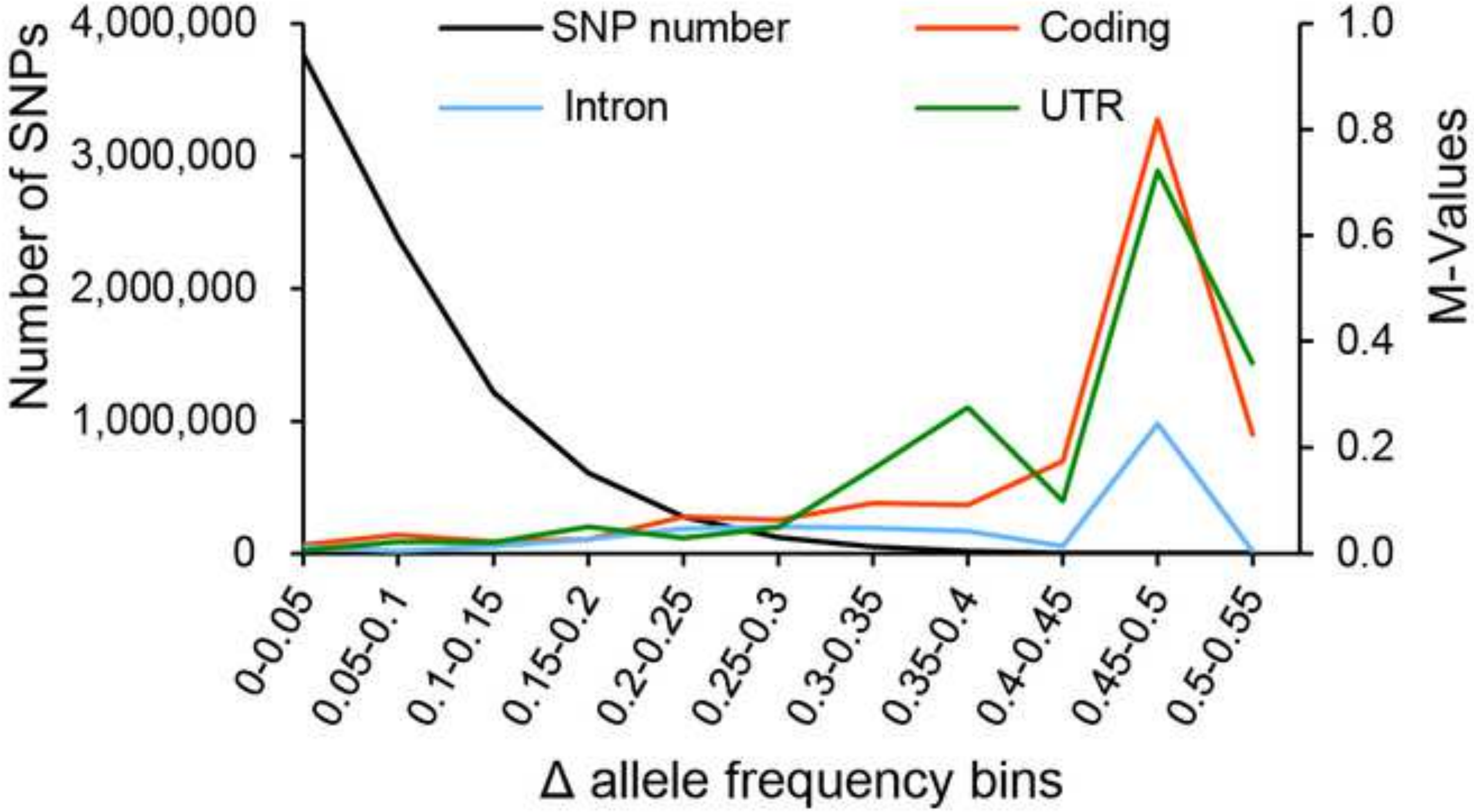

Figure

Click here to access/download;Figure;Figure4.tif

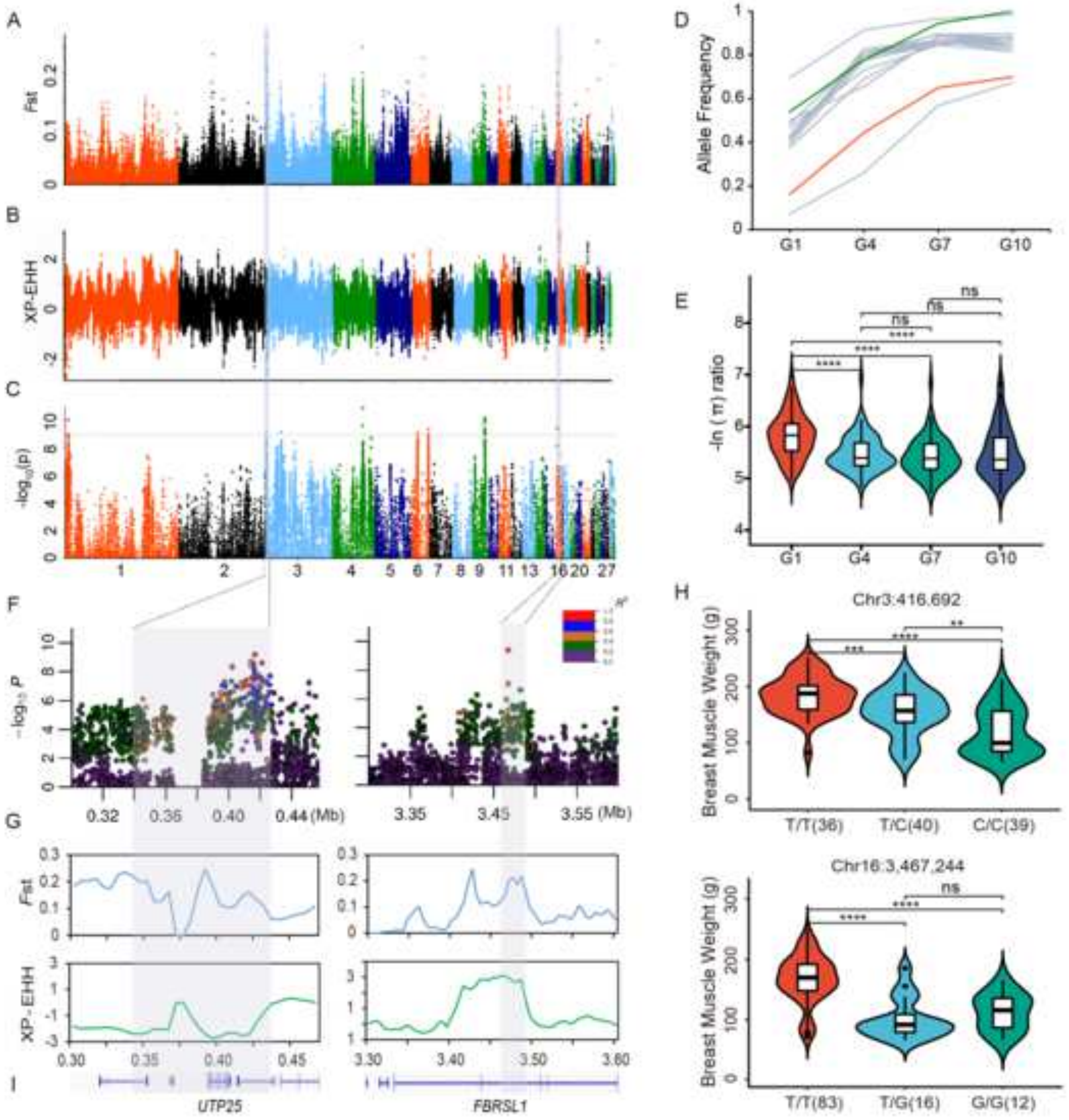

Figure Click here to access/download;Figure;Figure5.tif

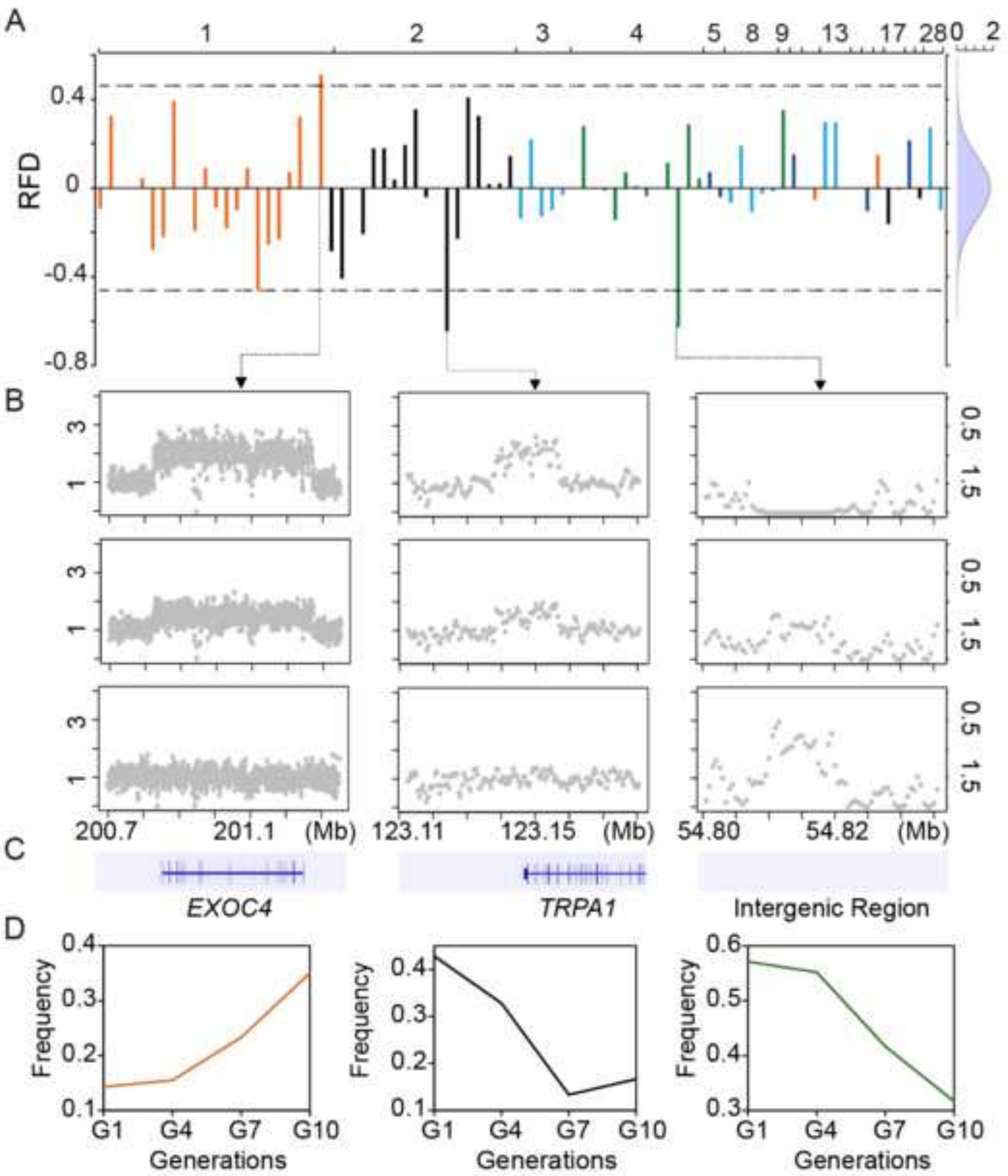

Supplement: giad016_GIGA-D-22-00268_Original_Submission [file giad016_giga-d-22-00268_original_submission.pdf]
