# Supplementary material for: Resequencing of a Pekin duck breeding population provides insights into the genomic response to short-term artificial selection
Source: Gigascience. 2023 Mar 27;12:giad016. doi: 10.1093/gigascience/giad016 (PMC10041536; doi:10.1093/gigascience/giad016)
Supplement: giad016_GIGA-D-22-00268_Revision_1 [file giad016_giga-d-22-00268_revision_1.pdf]

GigaScience

Resequencing of a Pekin duck breeding population provides insights into the genomic response to short-term artificial selection

--Manuscript Draft--

Manuscript Number:	GIGA-D-22-00268R1
Full Title:	Resequencing of a Pekin duck breeding population provides insights into the genomic response to short-term artificial selection
Article Type:	Research
Funding Information:	National Natural Science Foundation of China (31972523) — Prof. Zhengkui Zhou<br>National Ten Thousand Talent Program — Prof. Zhengkui Zhou<br>China Agricultural Research System (CARS-42-5) — Not applicable<br>the CAAS Innovation Team Project (ASTIP-IAS-9, CAAS-ZDRW202104) — Not applicable
Abstract:	Background<br><br>Short-term, intense artificial selection drives fast phenotypic changes in domestic animals and leaves imprints on their genomes. However, the genetic basis of this selection response is poorly understood. Here, we employed the Pekin duck Z2 pure line, in which the breast muscle weight was increased nearly three fold after ten generations of breeding. We de - novo assembled a high-quality reference genome of a female Pekin duck of this line (GCA_003850225.1) and identified 8.60 million genetic variants in 119 individuals among 10 generations of the breeding population.<br><br>Results<br><br>We identified 53 selected regions between the first and tenth generations, and 93.8% of the identified variations were enriched in regulatory and noncoding regions. Integrating the selection signatures and genome-wide association approach, we found that two regions covering 0.36 Mb containing UTP25 and FBRSL1 were most likely to contribute to breast muscle weight improvement. The major allele frequencies of these two loci increased gradually with each generation following the same trend. Additionally, we found that a copy number variation region containing the entire EXOC4 gene could explain 1.9% of the variance in breast muscle weight, indicating that the nervous system may play a role in economic trait improvement.<br><br>Conclusions<br><br>Our study not only provides insights into genomic dynamics under intense artificial selection but also provides resources for genomics-enabled improvements in duck breeding.
Corresponding Author:	Zhengkui Zhou<br>CAAS IAS: Chinese Academy of Agricultural Sciences Institute of Animal Science<br>Beijing, CHINA
Corresponding Author Secondary Information:	
Corresponding Author's Institution:	CAAS IAS: Chinese Academy of Agricultural Sciences Institute of Animal Science
Corresponding Author's Secondary Institution:	
First Author:	Simeng Yu
First Author Secondary Information:	
Order of Authors:	Simeng Yu

Powered by Editorial Manager® and ProduXion Manager® from Aries Systems Corporation

	Zihua Liu
	Ming Li
	Dongke Zhou
	Ping Hua
	Hong Cheng
	Wenlei Fan
	Yaxi Xu
	Dapeng Liu
	Suyun Liang
	Yunsheng Zhang
	Ming Xie
	Jing Tang
	Yu Jiang
	Shuisheng Hou
	Zhengkui Zhou
**Order of Authors Secondary Information:**	
**Response to Reviewers:**	Dear Dr. Zhang,<br>Thank you very much for handling our manuscript “Resequencing of a Pekin duck breeding population provides insights into the genomic response to short-term artificial selection” (GIGA-D-22-00268). We have carefully revised the manuscript according to the reviewers’ comments and your instructions. We appreciate all of the comments from the reviewers and have enclosed a revised manuscript as well as our point-by-point responses to the reviewers’ comments.<br>We believe that the revised manuscript has been greatly improved and hope it will meet the requirements of your journal. Thank you again for all of your assistance.<br><br>Sincerely yours,<br>Zhengkui Zhou and other coauthors<br><br>Reviewers’ Comments:<br>Reviewer #1:<br>1) In lines 35-38, The authors stated: "However, the genetic basis of short-term, intense artificial selection remains poorly understood. Although many studies have elucidated genetic mechanisms involved in intense artificial selection[4-8], most of them have not been genome-scale investigations." This might be true ten years ago, but there're many genomic-scale studies nowadays. Please cite important review papers instead and revise this paragraph.<br>Response: Thank you so much for your elaborative review and giving the precious advices. We have cited new important papers instead and rephrased this paragraph in line 35-42 of the revision into “Artificial selection experiments can help to understand the mechanism that allows populations to adapt to strong selection pressure[1, 2]. In combination with population-level genome sequencing, attempts have been made to identify alleles whose frequencies change systematically during selection experiments[3-5]. However, most studies were focused on lower organisms and were retrospective or used a single time point to characterize the dynamic changes in allele frequencies[1, 6-8]. Selecting a suitable domestic animal model for continuous high-intensity artificial selection trials may improve our understanding of the genetic basis of complex traits in domestic animals.”<br>References:<br>[1] Fuller RC, Baer CF and Travis J. How and When Selection Experiments Might Actually be Useful. Integr Comp Biol. 2005;45 3:391-404. doi:10.1093/icb/45.3.391.<br>[2] Lou RN, Therkildsen NO and Messer PW. The Effects of Quantitative Trait Architecture on Detection Power in Short-Term Artificial Selection Experiments. G3 (Bethesda). 2020;10 9:3213-27. doi:10.1534/g3.120.401287.

Powered by Editorial Manager® and ProduXion Manager® from Aries Systems Corporation

[3] Zhang H, Liang Q, Wang N, Wang Q, Leng L, Mao J, et al. Microevolutionary dynamics of chicken genomes under divergent selection for adiposity. iScience. 2020;23 6:101193. doi:10.1016/j.isci.2020.101193.
[4] Zan Y, Sheng Z, Lillie M, Ronnegard L, Honaker CF, Siegel PB, et al. Artificial Selection Response due to Polygenic Adaptation from a Multilocus, Multiallelic Genetic Architecture. Molecular Biology and Evolution. 2017;34 10:2678-89. doi:10.1093/molbev/msx194.
[5] Marks HL. Long-term selection for body weight in Japanese quail under different environments. Poult Sci. 1996;75 10:1198-203. doi:10.3382/ps.0751198.
[6] Castro JP, Yancoskie MN, Marchini M, Belohlavy S, Hiramatsu L, Kučka M, et al. An integrative genomic analysis of the Longshanks selection experiment for longer limbs in mice. Elife. 2019;8 doi:10.7554/eLife.42014.
[7] Kelly JK and Hughes KA. Pervasive Linked Selection and Intermediate-Frequency Alleles Are Implicated in an Evolve-and-Resequencing Experiment of Drosophila simulans. Genetics. 2019;211 3:943-61. doi:10.1534/genetics.118.301824.
[8] Seabra SG, Fragata I, Antunes MA, Faria GS, Santos MA, Sousa VC, et al. Different Genomic Changes Underlie Adaptive Evolution in Populations of Contrasting History. Molecular Biology and Evolution. 2018;35 3:549-63. doi:10.1093/molbev/msx247.

2) In lines 59-65, those sentences were repeated almost exactly in the Data Description section. Please rephrase.
Response: We have rephrased these sentences in line 63-67 of the revision into “Herein, we adopted a Pekin duck pure line selected for breast muscle weight for 10 generations. The foundation stock was the local conserved population in Beijing, China. After 10 generations of high-intensity artificial selection, the breast muscle weight of the Pekin duck Z2 line increased from 80 g to 220 g. These data were investigated to elucidate the dynamic response patterns in the Pekin duck genome under artificial selection.”

3) In line 84, "to assist in splicing the PacBio contigs into scaffolds", "splicing" should be the wrong word.
Response: Thanks for pointing out this error. We have modified the wrong words and expressions in line 84. The modified expression is “to link the PacBio contigs into scaffolds”.

4) In lines 85-87, please explain why the scaffold numbers were greater after clustering by the Hi-C data than those using optical mapping while the scaffold N50 was significantly improved but contig N50 decreased.
Response: Thanks for the pertinent evaluations. The software we used for Hi-C data-assisted assembly was 3d-DNA.This software has a process of re-interrupting contigs during chromosome loading. Thus, more fragmented fragments were generated. After manual error correction and two iterations of error correction, the quality of scaffolds was improved. However, many fragments that were considered inaccurate and could not be corrected by chromatin interaction were also retained and counted. Thus, the contig N50 decreased.

5) In lines 104-106, the authors stated: "we conducted the resequencing of 30 individuals (15 males, 15 females) from G1 to G10 in intervals of 3 generations.", I wonder how the total number could add up to 119.
Response: Apologize for our unclear statement. Our sampling schedule was to collect the blood samples of 30 ducks and phenotypic data of each individual (15 males and 15 females per generation) in the first, fourth, seventh and tenth generations, respectively. A total of 120 duck samples were obtained (one sample was lost in the fourth generation), and a total of 119 samples were obtained finally. We have added the description of the sampling plan to the 'Materials and Methods' section, on lines 309-312 of the revision.

6) In lines 189-190, the authors stated: "In total, we identified 2 duplicated CNVRs and 1 deletion between the G1 and G10 populations (Fig. 5A and B)." This can be misinterpreted as these CNVRs only occurred in the G10 population but not in the G1 population. Please rephrase.
Response: Apologize for our unclear description about the result. We have rephrased the sentence in lines 195-196 into “In total, we identified 2 duplicated CNVRs and 1

Powered by Editorial Manager® and ProduXion Manager® from Aries Systems Corporation

deletion with significant allele frequency changes between the G1 and G10 populations (Fig. 5A and B)" make its meaning clearer and more specific.

7) In line 201, I doubt that "intelligence" should be applied to animals in this case, and I found that the interpretation in lines 202-205 was pretty unconvincing, especially while EXOC4 is also highly expressed in mammalian skeletal muscle. TRPA1 is also involved in skeletal muscle repair in mammals. However, the functions of these genes in muscle were not properly considered in the manuscript.
Response: Thank you for the comment, we agree with you. We have deleted the interpretation in lines 203-205 in the original manuscript and rephrased the sentence into "Combined with analysis with transcriptome data, showed that EXOC4 was widely expressed in various periods and tissues and the expression level in breast muscle, sebum and brain was higher than that in other tissues (Supplementary Fig. S12A)". We also added the gene function of EXOC4 and TRPA1 to the Discussion section in the revision. A new section on the Discussion is reproduced here: "In this study, we identified 2 duplication CNVRs were annotated in two genes EXOC4 and TRPA1 respectively. EXOC4 may affect the development of breast muscle in Pekin ducks by affecting glucose transport and insulin synthesis. Studies have found that EXOC4 was involved in insulin synthesis and glucose transport in skeletal muscle [73-76]. As a component of the exocyst complex, EXOC4 is required for targeting of Glut4 to the plasma membrane by insulin [75]. Our results should that after ten years of artificial selection, the number of individuals with multiple copies of EXOC4 gene increases in the population of Pekin ducks. We speculate that the duplication makes fold increase of EXOC4 proteins, and a large number of EXOC4 proteins facilitate glucose transport to cells. Since cells become more efficient at taking up glucose, the excess glucose can be converted to fat and amino acids and stored by the body. Therefore, this may be one of the factors affecting the change of breast muscle weight of Pekin ducks. However, this requires confirmation. There is solid evidence demonstrating that TRPA1 is expressed throughout the mammalian body and has potential beneficial effects on systemic metabolism, including glucose metabolism [77]. Growing experimental evidence suggests that TRPA1 plays an important role in weight gain, obesity and insulin secretion [42, 77-81]. In Pekin ducks, we did not observe the high expression level of TRPA1 in breast muscle, but the body weight increased with the increase of breast muscle weight. Previously, our group has also demonstrated that there is a high genetic and phenotypic correlation between breast muscle weight and body weight in Pekin ducks (0.83 and 0.80) [33]. We surmise that TRPA1 indirectly affects the breast muscle weight of Pekin ducks by affecting body weight."
References:

[33] Xu Y, Hu J, Zhang Y, Guo Z, Huang W, Xie M, et al. Selection response and estimation of the genetic parameters for multidimensional measured breast meat yield related traits in a long-term breeding Pekin duck line. Asian-Australasian Journal Animal Science. 2018;31 10:1575-80. doi:10.5713/ajas.17.0837.
[42] Wu J, Wang J, Yue B, Xing-Tang F, Zhang C, Ma Y, et al. Research on association between variants and haplotypes of TRPV1 and TRPA1 genes with growth traits in three cattle breeds. Animal Biotechnology. 2019;30 3:202-11. doi:10.1080/10495398.2018.1470530.
[73] Ewart M-A, Clarke M, Kane S, Chamberlain LH and Gould GW. Evidence for a role of the exocyst in insulin-stimulated Glut4 trafficking in 3T3-L1 adipocytes. Journal of Biological Chemistry. 2005;280 5:3812-6. doi:10.1074/jbc.m409928200.
[74] Fulcher F, Smith B, Russ M and Patel Y. Dual role for myosin II in GLUT4-mediated glucose uptake in 3T3-L1 adipocytes. Experimental Cell Research. 2008;314 17:3264-74. doi:10.1016/j.yexcr.2008.08.007.
[75] Inoue M, Chang L, Hwang J, Chiang S-H and Saltiel AR. The exocyst complex is required for targeting of Glut4 to the plasma membrane by insulin. Nature. 2003;422 6932:629-33. doi:10.1038/nature01533.
[76] Kee AJ, Yang L, Lucas CA, Greenberg MJ, Martel N, Leong GM, et al. An actin filament population defined by the tropomyosin Tpm3.1 regulates glucose uptake. Traffic. 2015;16 7:691-711. doi:10.1111/tra.12282.
[77] Derbenev AV and Zsombok A. Potential therapeutic value of TRPV1 and TRPA1 in diabetes mellitus and obesity. Seminars In Immunopathology. 2016;38 3:397-406. doi:10.1007/s00281-015-0529-x.
[78] Mahajan N, Khare P, Kondepudi KK and Bishnoi M. TRPA1: Pharmacology, natural activators and role in obesity prevention. European Journal of Pharmacology. 2021;912:174553. doi:10.1016/j.ejphar.2021.174553.

Powered by Editorial Manager® and ProduXion Manager® from Aries Systems Corporation

[79] Kagawa Y, Ozaki-Masuzawa Y, Hosono T and Seki T. Garlic oil suppresses high-fat diet induced obesity in rats through the upregulation of UCP-1 and the enhancement of energy expenditure. Experimental and Therapeutic Medicine. 2020;19 2:1536-40. doi:10.3892/etm.2019.8386.
[80] Khare P, Jagtap S, Jain Y, Baboota RK, Mangal P, Boparai RK, et al. Cinnamaldehyde supplementation prevents fasting-induced hyperphagia, lipid accumulation, and inflammation in high-fat diet-fed mice. Biofactors. 2016;42 2:201-11. doi:10.1002/biof.1265.
[81] Kim MJ, Son HJ, Song SH, Jung M, Kim Y and Rhyu MR. The TRPA1 agonist, methyl syringate suppresses food intake and gastric emptying. PLoS One. 2013;8 8:e71603. doi:10.1371/journal.pone.0071603.

8) Potential functions of UTP25 and FBRSL1 in muscles were not considered and discussed.
Response: Thanks for your suggestion. We have adapted your advice and added some discussion about the potential functions of UTP25 and FBRSL1 in breast muscles in line 239-248 of the revision. Expression levels of the UTP25 and FBRSL1 genes at different developmental periods of Pekin duck Z2 line were tracked to increase the persuasiveness of the results and discussions. Thanks again for your comments and best wishes.
The new section in the Results is reproduced here: “We then tracked the expression levels of UTP25 and FBRSL1 genes in the breast muscles of different developmental periods of Pekin duck Z2 line. The results illustrated that the expression level of FBRSL1 decreased with the increase of days, while UTP25 was continuously expressed in breast muscle (Supplementary Fig. S11).”
The new section in the Discussion is reproduced here: “In this study, we identified two genes UTP25 and FBRSL1 significantly associated with breast muscle weight. FBRSL1 belongs to the Polycomb group (PcG) gene and is essential for many biological processes in mammals, including stem cell maintenance and differentiation [53, 54]. Our results showed that the expression level of FBRSL1 in the breast muscle of Pekin duck Z2 line decreased with the increase of age. This may hint at FBRSL1 was strongly selected to promote the development of Pekin duck breast muscle. UTP25 (also named DIEXF in human) reportedly affects digestive organ expansion by regulating p53 pathway [55, 56]. However, the function of UTP25 has not been characterized in animal muscles. UTP25 was strongly selected in the continuous breeding process of Pekin duck Z2 line, and further investigation would be necessary to elucidate its functions.”
References:
[53] Bathla S, Rawat P, Baithalu R, Yadav ML, Naru J, Tiwari A, et al. Profiling of urinary proteins in Karan Fries cows reveals more than 1550 proteins. J Proteomics. 2015;127 Pt A:193-201. doi:10.1016/j.jprot.2015.05.026.
[54] Chen Z, Yao Y, Ma P, Wang Q and Pan Y. Haplotype-based genome-wide association study identifies loci and candidate genes for milk yield in Holsteins. PLoS One. 2018;13 2:e0192695. doi:10.1371/journal.pone.0192695.
[55] Zhang S-J, Liu C-J, Yu P, Zhong X, Chen J-Y, Yang X, et al. Evolutionary Interrogation of Human Biology in Well-Annotated Genomic Framework of Rhesus Macaque. Molecular Biology and Evolution. 2014;31 5:1309-24. doi:10.1093/molbev/msu084.
[56] Chen J, Ruan H, Ng SM, Gao C, Soo HM, Wu W, et al. Loss of function of def selectively up-regulates Delta113p53 expression to arrest expansion growth of digestive organs in zebrafish. Genes & Development. 2005;19 23:2900-11.

Supplemental Figure S13. Expression levels of the UTP25 and FBRSL1 genes at different developmental periods of Pekin duck Z2 line.

9) Gene annotation methods were not described in the Methods section.

Powered by Editorial Manager® and ProduXion Manager® from Aries Systems Corporation

Response: The genome assembly was annotated by the NCBI Eukaryotic Genome Annotation Pipeline, an automated pipeline that annotates genes, transcripts and proteins on draft and finished genome assemblies. We did not manually correct the annotation of any gene. As suggested by the reviewer, we have described the pipeline used for the reference genome annotation in the Methods section of the revision and cited the relevant reference.

10) What are the three panels in Fig. 5B?
Response: Sorry for the confusing figure. Fig.5B is examples of copy number variation of selected CNV in different generations. We have added a detailed explanation of the meaning of Fig.5B to the Figure Legends section of the manuscript.

Reviewer #2:
1) While the Breast muscle yield of the Pekin line Z2 significantly changed from 80g to 220g over 10 generation of selection, details of this line with respect to its prime trait, 6Week's live weight, feed efficiency and other breast yield as a proportion of total meat-yield should be provided in the MS. It is obvious to detail that when breast yield improved more than 2 fold over the 10 generation's period, how good were the other meaty-cuts with reference to breast muscle yield. The Breeding process of Z2 (page 7) needs appropriate elaboration too.
Response: Thank you so much for your elaborative review and giving the precious advices, which help us to improve our manuscript. In the Supplementary Materials, we have added supplementary figures showing the changes of other prime traits of Pekin Duck Z2 line among generations. As evident from the supplementary figure that the live weight, breast muscle volume, leg muscle weight, subcutaneous fat rate, abdominal fat rate and total evisceration rate increased gradually among the ten generations. The supplementary figure is reproduced here:

Supplementary Figure S4. Generational average phenotypic values of prime traits of Pekin duck Z2 line. The abscissa denotes the generation.
The breeding process of Z2 line was described in detail in the section of Materials and Methods. Please refer to the 287-308 lines of the revision. We reproduce the detailed description here: "Z2 line originated from the initial conserved population of Pekin duck in Beijing. Pekin duck Z2 line was selected as the research object, because it has many characteristics, including: a it is bred in closed group, with pure pedigree and clear genetic background; b the selection pressure of this line is constant, which is favorable for the accumulation of alleles gradually; c at the age of 6 weeks each generation, 15 male and 15 female ducks were randomly selected from a large population for slaughter test to measure their breast muscle weight and retain blood samples; d the breeding of all generations was completed in Pekin duck breeding Base, Changping District, Beijing, and the performance measurement was done in spring, resulting in little difference in environmental effect between generations. Besides that, all ducks were kept in similar environment and had free access to water and feed pellets [33]. We measured the breast muscle weight of the whole population in vivo at the age of 6 weeks of each generation, and selected ducks with the heavier breast muscle weight as parents to produce the next generation. About 750 individuals in each generation were retained for breeding, of which 35 % – 40 % retention rate for female ducks and 7 % – 8 % retention rate for male ducks. The inbreeding was strictly avoided by calculating the inbreeding coefficient of each generation. The breast muscle weight (BMW) trait was estimated by breast muscle volume (BMV), breast width (BB), keel length (KL) and breast muscle thickness (BMT) (Fig. 2A, Supplementary Fig. S5). The correlation equation between these traits was: $BMV=BB\times KL\times BMT$; $BMW = 0.6228\times BMV+17.042$ [19]. BB and KL were measured by vernier calipers, while BMT was measured by ultrasound scanning technology. The correlation parameters of BMW and BMV are real and reliable numerical results based on the measured breast muscle weight of years of slaughter experiments and fitted by linear regression equation model."

2) While most narration on Re-sequencing of the generation specific DNA samples are relevant, making a lot of descriptions by including many digital large images appear redundant, and therefore, inclusion of too many images to drive home the points need to be looked at thoroughly. Why need more than 2 or 3 images, when the facts can be well-cited as texts!
Response: Sorry for our redundant information. We have deleted and merged the

redundant attached images.

3) For a study of such elaborate dimension, the discussion appears little too condensed, and therefore, be optimized to discuss, how the last 10 generations of selection brought so much of perceptible changes at molecular level, which were concerning more to non-coding regions than coding sequences. Discussions also may be beefed up citing experience of other genome studies (even beyond chickens and turkeys, to other food-purpose livestocks), for why CNV regions could be more important hotspots to reveal selection-induced molecular changes in Pekin ducks.
Response: Thank you for your valuable advice. We have optimized the discussion section based on your suggestions. The new section in the Discussion is reproduced here: “It can be anticipated that CNVs may be more important hotspots to reveal selection-induced molecular changes in Pekin ducks. First of all, CNVs are widespread in domesticated animals, such as pigeon, sheep, pig, chicken, cattle, horse, and dog [57-66]. Moreover, the identified CNVs were largely found in genes that encode growth factors and receptors, and genes related to development and play a role mainly through duplication [67]. Secondly, CNV may have a role in rapid adaptation under strong selective pressure. This phenomenon was found in our experimental population that 81 copy number variations were identified in the Pekin duck genome under ten generations of high-intensity selection. Similar phenomena were found in experimental evolution studies in microbes under nutrient limitation and multicellular systems [68-70]. Finally, and most importantly, there is evidence for CNVs under selection in domesticated species. During domestication, CNVs underlying domestication traits increase in frequency in the population in response to selection, and genomic signatures for selection can sometimes be detected associated with these CNVs [57, 71, 72].”

References:

[57] Bruders R, Van Hollebeke H, Osborne EJ, Kronenberg Z, Maclary E, Yandell M, et al. A copy number variant is associated with a spectrum of pigmentation patterns in the rock pigeon (Columba livia). PLoS Genetics. 2020;16 5:e1008274. doi:10.1371/journal.pgen.1008274.
[58] Ghosh S, Qu Z, Das PJ, Fang E, Juras R, Cothran EG, et al. Copy number variation in the horse genome. PLoS Genetics. 2014;10 10:e1004712. doi:10.1371/journal.pgen.1004712.
[59] Guo Y, Gu X, Sheng Z, Wang Y, Luo C, Liu R, et al. A Complex Structural Variation on Chromosome 27 Leads to the Ectopic Expression of HOXB8 and the Muffs and Beard Phenotype in Chickens. PLoS Genetics. 2016;12 6:e1006071. doi:10.1371/journal.pgen.1006071.
[60] Lee YL, Takeda H, Costa Monteiro Moreira G, Karim L, Mullaart E, Coppieters W, et al. A 12 kb multi-allelic copy number variation encompassing a GC gene enhancer is associated with mastitis resistance in dairy cattle. PLoS Genetics. 2021;17 7:e1009331. doi:10.1371/journal.pgen.1009331.
[61] Qiu Y, Ding R, Zhuang Z, Wu J, Yang M, Zhou S, et al. Genome-wide detection of CNV regions and their potential association with growth and fatness traits in Duroc pigs. BMC Genomics. 2021;22 1:332. doi:10.1186/s12864-021-07654-7.
[62] Liu M, Fang L, Liu S, Pan MG, Seroussi E, Cole JB, et al. Array CGH-based detection of CNV regions and their potential association with reproduction and other economic traits in Holsteins. BMC Genomics. 2019;20 1 doi:10.1186/s12864-019-5552-1.
[63] Stafuzza NB, Silva RMO, Fragomeni BO, Masuda Y, Huang Y, Gray K, et al. A genome-wide single nucleotide polymorphism and copy number variation analysis for number of piglets born alive. BMC Genomics. 2019;20 1:321. doi:10.1186/s12864-019-5687-0.
[64] Yuan C, Lu Z, Guo T, Yue Y, Wang X, Wang T, et al. A global analysis of CNVs in Chinese indigenous fine-wool sheep populations using whole-genome resequencing. BMC Genomics. 2021;22 1:78. doi:10.1186/s12864-021-07387-7.
[65] Salmon Hillbertz NH, Isaksson M, Karlsson EK, Hellmen E, Pielberg GR, Savolainen P, et al. Duplication of FGF3, FGF4, FGF19 and ORAOV1 causes hair ridge and predisposition to dermoid sinus in Ridgeback dogs. Nature Genetics. 2007;39 11:1318-20. doi:10.1038/ng.2007.4.
[66] Pruitt KD, Brown GR, Hiatt SM, Thibaud-Nissen F, Astashyn A, Ermolaeva O, et al. RefSeq: an update on mammalian reference sequences. Nucleic Acids Research. 2014;42 Database issue:D756-63. doi:10.1093/nar/gkt1114.
[67] Lye ZN and Purugganan MD. Copy Number Variation in Domestication. Trends in

	Plant Science. 2019;24 4:352-65. doi:10.1016/j.tplants.2019.01.003.<br>[68] DeBolt S. Copy number variation shapes genome diversity in Arabidopsis over immediate family generational scales. Genome Biology and Evolution. 2010;2:441-53. doi:10.1093/gbe/evq033.<br>[69] Gresham D, Desai MM, Tucker CM, Jenq HT, Pai DA, Ward A, et al. The repertoire and dynamics of evolutionary adaptations to controlled nutrient-limited environments in yeast. PLoS Genet. 2008;4 12:e1000303. doi:10.1371/journal.pgen.1000303.<br>[70] Farslow JC, Lipinski KJ, Packard LB, Edgley ML, Taylor J, Flibotte S, et al. Rapid Increase in frequency of gene copy-number variants during experimental evolution in Caenorhabditis elegans. BMC Genomics. 2015;16:1044. doi:10.1186/s12864-015-2253-2.<br>[71] Axelsson E, Ratnakumar A, Arendt M-L, Maqbool K, Webster MT, Perloski M, et al. The genomic signature of dog domestication reveals adaptation to a starch-rich diet. Nature. 2013;495 7441:360-4. doi:10.1038/nature11837.<br>[72] Reiter T, Jagoda E and Capellini TD. Dietary Variation and Evolution of Gene Copy Number among Dog Breeds. PLoS One. 2016;11 2:e0148899. doi:10.1371/journal.pone.0148899.<br><br>4) As the two sites: [candidate genes] UTP25 and FBRSL1 showed significant changes/linkages to progressing selection for breast muscle yield, across generations, a sound discussion on their roles in ducks in terms of meat yield, meat quality, and breast meat composition etc… can be welcome!<br>Response: Thanks for your comments. We have adapted your advice and added some discussion about the potential functions of UTP25 and FBRSL1 in muscles in line 239-248 of the revision.<br>The new section in the Discussion is reproduced here: “In this study, we identified two genes UTP25 and FBRSL1 significantly associated with breast muscle weight. FBRSL1 belongs to the Polycomb group (PcG) gene and is essential for many biological processes in mammals, including stem cell maintenance and differentiation [53, 54]. Our results showed that the expression level of FBRSL1 in the breast muscle of Pekin duck Z2 line decreased with the increase of age. This may hint at FBRSL1 was strongly selected to promote the development of Pekin duck breast muscle. UTP25 (also named DIEXF in human) reportedly affects digestive organ expansion by regulating p53 pathway [55, 56]. However, the function of UTP25 has not been characterized in animal muscles. UTP25 was strongly selected in the continuous breeding process of Pekin duck Z2 line, and further investigation would be necessary to elucidate its functions.”<br>[53] Bathla S, Rawat P, Baithalu R, Yadav ML, Naru J, Tiwari A, et al. Profiling of urinary proteins in Karan Fries cows reveals more than 1550 proteins. J Proteomics. 2015;127 Pt A:193-201. doi:10.1016/j.jprot.2015.05.026.<br>[54] Chen Z, Yao Y, Ma P, Wang Q and Pan Y. Haplotype-based genome-wide association study identifies loci and candidate genes for milk yield in Holsteins. PLoS One. 2018;13 2:e0192695. doi:10.1371/journal.pone.0192695.<br>[55] Zhang S-J, Liu C-J, Yu P, Zhong X, Chen J-Y, Yang X, et al. Evolutionary Interrogation of Human Biology in Well-Annotated Genomic Framework of Rhesus Macaque. Molecular Biology and Evolution. 2014;31 5:1309-24. doi:10.1093/molbev/msu084.<br>[56] Chen J, Ruan H, Ng SM, Gao C, Soo HM, Wu W, et al. Loss of function of def selectively up-regulates Delta113p53 expression to arrest expansion growth of digestive organs in zebrafish. Genes & Development. 2005;19 23:2900-11.
**Additional Information:**	
**Question**	**Response**
Are you submitting this manuscript to a special series or article collection?	No
**Experimental design and statistics**<br><br>Full details of the experimental design and statistical methods used should be given	Yes

Powered by Editorial Manager® and ProduXion Manager® from Aries Systems Corporation

in the Methods section, as detailed in our Minimum Standards Reporting Checklist. Information essential to interpreting the data presented should be made available in the figure legends.<br><br>Have you included all the information requested in your manuscript?	
**Resources**<br><br>A description of all resources used, including antibodies, cell lines, animals and software tools, with enough information to allow them to be uniquely identified, should be included in the Methods section. Authors are strongly encouraged to cite Research Resource Identifiers (RRIDs) for antibodies, model organisms and tools, where possible.<br><br>Have you included the information requested as detailed in our Minimum Standards Reporting Checklist?	Yes
**Availability of data and materials**<br><br>All datasets and code on which the conclusions of the paper rely must be either included in your submission or deposited in publicly available repositories (where available and ethically appropriate), referencing such data using a unique identifier in the references and in the "Availability of Data and Materials" section of your manuscript.<br><br>Have you have met the above requirement as detailed in our Minimum Standards Reporting Checklist?	Yes

Manuscript
Click here to access/download;Manuscript;Manuscript-Pekin duck- copyedits_SCE(1).docx

Resequencing of a Pekin duck breeding population provides insights into the genomic response to short-term artificial selection

Authors

Simeng Yu[1], Zihua Liu[2], Ming Li[2], Dongke Zhou[2], Ping Hua[2], Hong Cheng[2], Wenlei Fan[1], Yaxi Xu[1], Dapeng Liu[1], Suyun Liang[1], Yunsheng Zhang[1], Ming Xie[1], Jing Tang[1], Yu Jiang[2], Shuisheng Hou[1], Zhengkui Zhou[1*]

[1]State Key Laboratory of Animal Nutrition; Key Laboratory of Animal (Poultry) Genetics Breeding and Reproduction, Ministry of Agriculture and Rural Affairs; Institute of Animal Science, Chinese Academy of Agricultural Sciences, Beijing 100193, China

[2]Key Laboratory of Animal Genetics, Breeding and Reproduction of Shaanxi Province, College of Animal Science and Technology, Northwest A&F University, Yangling 712100, China

[*]Corresponding author. E-mail: zhouzhengkui@caas.cn.

Simeng Yu [0000-0003-1093-546X];
Wenlei Fan [0000-0003-0092-0512];
Yaxi Xu [0000-0001-9879-6147];
Dapeng Liu [0000-0001-5865-4937];
Jing Tang [0000-0001-7653-8438];
Yu Jiang [0000-0003-4821-3585];
Zhengkui Zhou [0000-0002-5192-2465]

Abstract

Background: Short-term, intense artificial selection drives fast phenotypic changes in domestic animals and leaves imprints on their genomes. However, the genetic basis of this selection response is poorly understood. To better address this we employed the Pekin duck Z2 pure line, in which the breast muscle weight was increased nearly threefold after ten generations of breeding. We *de-novo* assembled a high-quality reference genome of a female Pekin duck of this line (GCA_003850225.1) and identified 8.60 million genetic variants in 119 individuals among 10 generations of the breeding population. **Results:** We identified 53 selected regions between the first and tenth generations, and 93.8% of the identified variations were enriched in regulatory and noncoding regions. Integrating the selection signatures and genome-wide association approach, we found that two regions covering 0.36 Mb containing *UTP25* and *FBRSL1* were most likely to contribute to breast muscle weight improvement. The major allele frequencies of these two loci increased gradually with each generation following the same trend. Additionally, we found that a copy number variation region containing the entire *EXOC4* gene could explain 1.9% of the variance in breast muscle weight, indicating that the nervous system may play a role in economic trait improvement. **Conclusions:** Our study not only provides insights into genomic dynamics under intense artificial selection but also provides resources for genomics-enabled improvements in duck breeding.

Keywords: duck; genome; artificial selection; breast muscle weight; selection signatures

Background

Artificial selection experiments can help to understand the mechanism that allows populations to adapt to strong selection pressure[1, 2]. In combination with population-level genome sequencing, attempts have been made to identify alleles whose frequencies change systematically during selection experiments[3-5]. However, most studies were focused on lower organisms and were retrospective or used a single time point to characterize the dynamic changes in allele frequencies[1, 6-8]. Selecting a suitable domestic animal model for continuous high-intensity artificial selection trials may improve our understanding of the genetic basis of complex traits in domestic animals.

Studies of two representative bidirectional selected resource populations in which chicken body weight[9-13] and abdominal fat[14-18] were selected have shown that continuous artificial selection results in obvious phenotypic and genomic differentiation in poultry. Artificial selection signals have also been identified in animals, such as rabbits[19], sheep[20], goats[21, 22] and pigs[23]. However, many domestic animal populations established for selection experiments are not as complete as chicken populations because of the long generation interval and sequencing costs in these species. Ducks (*Anas platyrhynchos*) (NCBI:txid8839) are among the most economically important waterfowl; they can provide meat, eggs and down for humans, and show important characteristics, such as a short generation interval, high reproductive ability and a long but traceable history of artificial selection[24]. Moreover, ducks, like other birds, have smaller genomes than non-avian terrestrial vertebrates. These characteristics make the duck an effective model for studying genomic footprints of artificial selection.

High-quality reference genomes are the foundation for genetic research and molecular marker breeding, which can support innovations in sustainable animal production[25, 26]. To date, reference genomes have been published for a variety of domestic animals such as ducks, chickens, pigs, cattle, sheep and goats [24, 27-31], providing important resources for livestock and poultry genetic breeding. To completely capture the variation present in the genome of our study population, we chose a female individual from our population for PacBio long-read sequencing and genome assembly to obtain a high-quality chromosome-level reference genome of Pekin duck (GCA_003850225.1) (**Fig. 1A**).

Herein, we utilized a Pekin duck pure line selected for breast muscle weight for 10 generations. The foundation stock was the local conserved population in Beijing, China. After 10 generations of high-intensity artificial selection, the breast muscle weight of the Pekin duck Z2 line increased from 80 g to 220 g. These data were investigated to elucidate the dynamic response patterns in the Pekin duck genome under artificial selection.

Data Description

To understand the genetic basis of short-term, intense artificial selection, we utilized a Pekin duck pure line selected for breast muscle weight at 6 weeks of age. We implemented the whole-genome resequencing of 119 ducks across 10 generations (one generation per year, 2005-2014) and mapped the resequencing data to the high-quality reference genomes assembled in this study. Using 8.60 million of single nucleotide polymorphisms (SNPs), we tested for dynamic changes in population structure under short-term intense selection and we assessed the genome for signatures of short-term intense selection associated with breast muscle weight.

Analyses

An improved Pekin duck genome assembly

To carry out the *de novo* assembly of the Pekin duck genome, we adopted a combination of PacBio long-read sequencing, BioNano optical mapping and Hi-C technologies. We first generated 65.9 Gb of PacBio long reads with 50× genome coverage, 49 Gb of BioNano high-quality reads with 41× genome coverage, and 106 Gb of Hi-C reads with 82× genome coverage (**Table S1**). Then, these data were used to assemble the new duck genome (**Fig. 1A**). Assembly was performed in a stepwise fashion (**Supplementary Fig. S1**), to generate assemblies with improvements for each process. First, we used the initial PacBio subreads to construct 2682 contigs, yielding a contig N50 of 4.17 Mb (**Table 1**). Second, we used optical mapping (BioNano Genomics Irys) data to link the PacBio contigs into scaffolds, resulting in the population of 1,788 scaffolds, and the N50 was 6.24 Mb (**Table 1**). Then, the Hi-C data (**Supplementary Table S2**) were used to cluster the scaffolds at the chromosomal scale, resulting in 1852 scaffolds

(**Supplementary Fig. S2**). The final assembled genome length was 1.12 Gb, with scaffold N50 and contig N50 values of 76.13 Mb and 4.10 Mb, respectively (**Table 1**).

Assembly	Number of scaffolds	Scaffold N50 (Mb)	Genome size
PacBio	—	—	1,154,570,803
PacBio+BioNano	1788	6.24	1,153,069,495
PacBio+BioNano+Hi-C	1852	41.14	1,134,893,859
GCA_003850225.1	1330	76.13	1,134,894,103

We next evaluated the quality of the new assembly. BUSCO (BUSCO, RRID:SCR 015008)[32] (v5.1.2) assessments of the new assembly revealed 93.3% completeness (**Supplementary Fig. S3**). The continuity of our new assembly yielded a 62-fold improvement compared to that of BGI_duck_1.0 (76.13 vs. 1.23 Mb) (**Supplementary Table S3**). GCA_003850225.1 (IASCAAS_PekinDuck_PBH1.5) had fewer gaps than BGI_duck_1.0 (0.26% vs. 3.17%), which also indicated that our new assembly presented a higher level of integrity (**Fig. 1B-D, Supplementary Table S3**). After the annotation of the newly assembled reference genome, we obtained 22,079 annotated genes, representing an increase of 34.22% relative to BGI_duck_1.0. All of the above results indicated that the quality of our newly assembled Pekin duck genome was greatly improved compared with that of the BGI_duck_1.0 assembly. Furthermore, we mapped the resequencing data of 119 Pekin duck individuals (**Supplementary Table S4**) to BGI_duck_1.0 and GCA_003850225.1, and the mapping rate was significantly improved (**Fig. 1E**) (t test, $P <$ 2.22e-16) when using the new assembly.

To track the dynamic change process in the Pekin duck genome driven by intense selection, we conducted the resequencing of 30 individuals (15 males, 15 females) from G1 to G10 in intervals of 3 generations. We mapped all of the paired-end reads to the GCA_003850225.1 assembly with an average coverage rate of 94.77%, and an average depth of 7.08 × (6.26-8.01 ×) (**Supplementary Table S4**). These sequencing data enabled us to identify a total of 8.60 million single-nucleotide polymorphisms (SNPs) and 81 high-confidence copy number variation regions (CNVRs) among the 119 individuals.

Breeding process in the Pekin duck Z2 line

Breast muscle weight was calculated according to the following equations. First, breast muscle volume (BMV) = BB×KL×BMT, and BMW = 0.6228 × BMV + 17.042[33], where BB is breast breadth, KL is keel length, and BMT is breast muscle thickness. Herein, we used a Vernier caliper to measure BB and KL, while BMT was measured with B-ultrasound scanning technology (**Fig. 2A**). BMW was then estimated according to the BMV derived from the BB, KL, and BMT values. The statistical results showed that after ten generations of high-intensity artificial selection, the breast muscle weight of Pekin duck increased from 80 g to 220 g (**Fig. 2B, Supplementary Fig. S4**).

Dynamic changes in population structure under short-term intense selection

To examine genetic differentiation at the whole genome level in ten generations, we performed principal component analysis (PCA)[34] using the whole-genome SNP data of G1, G4, G7 and G10. Individuals across generations separated along the two principal component dimensions, and ducks between the G1 and G10 generations could be clearly separated into two clusters (**Fig. 2C**). In the G10 generation, population diversity showed a decreasing trend (**Fig. 2C, bottom right**). However, there were no significant differences in the proportions of nonsynonymous mutations and synonymous mutations (dN/dS) in the coding region between the four generations (**Supplementary Table S5**). The dN/dS ratio of all generations was below 1, indicating that the Pekin duck population was subjected to continuous negative selection.

To identify the dynamic changes in duck genomic variations, we calculated the allele frequency difference (ΔAF) between the G1 and G10 generations for each SNP and sorted these values into 5% bins (ΔAF = 0 to 0.05, etc.). We evaluated the enrichment of the SNPs in each bin in exons, introns, and untranslated regions (UTRs) to illustrate the numbers and distributions of sites that played a major role during artificial selection in the sequenced genomes. We observed a large number of allele frequency shifts in the entire data set, but no SNPs with $\Delta AF > 0.55$ were identified (**Fig. 3**), implying that the directional selection event related to the breast muscle weight of Pekin ducks was in accord with polygenetic and soft selective sweep patterns[35]. We found

significant enrichments of high ΔAF SNPs ($\Delta AF > 0.3$) in both UTRs and introns (χ^2 test, $P <$ 0.05), whereas in exons, the excess was only 2 SNPs (**Fig. 3, Supplementary Table S6**). We found that exonic SNPs tended to be significantly enriched in bins with $\Delta AF < 0.1$ (**Supplementary Table S6**). Therefore, changes in noncoding regions played an important role during the breeding process in the Pekin duck Z2 line.

Identification of signals under artificial selection

We employed a joint analysis strategy to calculate fixation index (*F*st)[36] values and cross-population extended haplotype homozygosity (XP-EHH)[37] values (10 kb window, 5 kb step) to identify potential selected regions between the G1 and G10 populations (**Fig. 4A and B, Supplementary Fig. S5**). Using the empirical quantiles of the top 1% of SNPs and taking the intersection of the *F*st (*F*st > 0.09) and XP-EHH (XP-EHH > 1.47, XP-EHH < -1.48) analysis values, we identified a total of 187 regions as potentially containing selective signals (**Supplementary Table S7**). Among these candidate regions, 59 genes were found across ~1.22 Mb (**Supplementary Table S8**). In addition, the results illustrated that the allele frequency of the potential selected regions identified by different approaches showed an upward trend over the ten generations (**Supplementary Fig. S6A and B**). The genetic diversity of the potential selected regions exhibited the opposite change trend among different generations (**Supplementary Fig. S7**). To exclude the potential selected regions that may be due to genetic drift, we then employed a genome-wide association study (GWAS) to identify the overlapping selection signatures associated with breast muscle weight. We applied a Bonferroni threshold of a $-\log_{10} P > 8.94$ value for outliers to identify selected SNPs associated with breast muscle weight, and a total of 22 SNPs reached the association analysis threshold (**Fig. 4C, Supplementary Table S9**). The allele frequencies of the 22 SNPs increased gradually over 10 generations (**Fig. 4D, Supplementary Fig. S6C**), indicating that these loci were subjected to continuous selection. After overlapping the associated SNPs with candidate selected regions, we identified 2 regions associated with breast muscle weight. These signals were located on chromosomes 3 and 16 (Chr3: 0.18-0.44 Mb; Chr16: 3.40-3.50 Mb). The genetic diversity of the two selected regions was significantly different between generations (**Fig. 4E**).

To accurately detect the genomic footprints left by artificial selection, we examined the linkage

disequilibrium (LD, expressed as r^2) of the top SNPs (Chr3:416,692 bp; Chr16:3,467,244 bp) and surrounding SNPs within these candidate regions. Then, we detected corrected signals (r^2 > 0.4) in the 0.34-0.43 Mb region of chromosome 3 and the 3.46-3.49 Mb region of chromosome 16, which were associated with breast muscle weight (**Fig. 4F and G**). The extent of LD between the variants within the candidate regions and the lead SNPs increased gradually over 10 generations (**Supplementary Fig. S8**). These regions spanned 120 kb and contained 2,072 SNPs. Notably, we found that a large proportion (93.87%, 1,945/2,072) of the SNPs in the candidate regions were located in noncoding regions (**Supplementary Fig. S9**). Thus, continuous breeding for breast muscle weight in Pekin ducks has a polygenic basis with many loci responding to continuous artificial selection and noncoding sequences may play an important role in Pekin duck breast muscle weight improvement.

Genotyping the ducks using the lead SNP located at Chr3:416,6929 (T>C) revealed that individuals carrying the variant T alleles exhibited heavier breast muscles (**Fig. 4I**). The top SNP on chromosome 16 (Chr16:3,467,244 T>G) showed the same trend (**Fig. 4I**). In addition, two genes (*UTP25* and *FBRSL1*) were identified in the two putative selected regions **(Fig. 4H**). Combined analysis with the global transcriptomic data of ducks, revealed that *UTP25* and *FBRSL1* were widely expressed in various tissues of Pekin ducks (**Supplementary Fig. S10**). We then tracked the expression levels of *UTP25* and *FBRSL1* genes in the breast muscles of different developmental periods of Pekin duck Z2 line. The results illustrated that the expression level of *FBRSL1* decreased with the increase of days, while *UTP25* was continuously expressed in breast muscle (**Supplementary Fig. S11**).

Identification of CNVRs under artificial selection

Copy number variations (CNVs) show higher mutational rates than SNPs[38], typically involve larger genomic regions and potentially affect a wide range of phenotypic traits[39-41]. Based on our new assembly, we obtained 81 CNVRs with high credibility and accuracy on autosomes (**Fig. 5A, Supplementary Table S10**). In total, we identified 2 duplicated CNVRs and 1 deletion with significant allele frequency changes between the G1 and G10 populations (**Fig. 5A and B**). The CNVRs detected in most individuals were duplications located on chromosome 1 at 200.83-201.27 Mb (CNV1) and chromosome 2 at 123.14-123.16 Mb (CNV2) (**Fig. 5B**), and these two

CNVRs were annotated in two genes *EXOC4* and *TRPA1* (**Fig. 5C**). In addition, the CNVR identified in most individuals (78%) was a homozygous copy number loss or hemizygous copy number loss on chromosome 4 at 54.81-54.82 Mb (CNV3) (**Supplementary Table S11**) and the allele frequency of this CNVR increased gradually over the 10 generations (**Fig. 5D**). Interestingly, the *EXOC4* gene was completely encompassed by CNV1. Combining this analysis with transcriptome data showed that *EXOC4* was widely expressed in various periods and tissues and the expression level in breast muscle, sebum and brain was higher than that in other tissues (**Supplementary Fig. S12A**). Similar to CNV1, only one gene *TRPA1* was located in CNV2. A study has shown that *TRPA1* is associated with growth traits (including body weight, body length, body height, etc.) in bovine[42].In Pekin ducks, we did not observe the high expression level of TRPA1 in breast muscle (**Supplementary Fig. S12B**), however, the body weight increased with the increase of breast muscle weight (**Supplementary Fig. S4**).

Discussion

Sequencing technology and comparative genomics have furthered our understanding of selection and variation. Based on long-term selection and phenotypic differentiation in livestock populations, several major genes controlling economically important traits in livestock have been identified[43-46]. However, the genetic mechanism of short-term, intense artificial selection remains unclear. Our work demonstrated how the response to short-term intense artificial selection targeting a complex trait—8-week breast muscle weight—in the Pekin duck Z2 line has been predominantly achieved by recruiting a large number of loci in the genome to undergo frequency shifts. This result was supported by the conclusions of previous studies on chicken experimental models[3, 4, 13].

Based on our new assembly, we analyzed the SNP information of each generation. We found that noncoding sequences have played a prominent role during Pekin duck breeding. This is can potentially be explained as on the one hand, compared with coding sequences, *cis*-regulatory elements of pleiotropic loci are now considered to be the main source of phenotypic differentiation[47-49]. And on the other hand, mutation bias reduces the mutation rate of functionally constrained regions[50]. After a new gene is formed by gene duplication, and establishes a clear function, the mutation frequency of its coding region is restricted[51]. Thus,

most of the mutations found in coding regions are synonymous mutations, and the mutation frequency in these regions is lower than that in noncoding region.

We did not find completely fixed loci in the genome of the Pekin duck Z2 line, although this population was subjected to high intensity artificial selection for ten generations. However, we detected allele frequency shifts at many loci throughout the Pekin duck genome. Two possible explanations may account for this finding. First, it is more common for complex quantitative traits to involve a combination of multiple standing variations at many loci. Such polygenic adaptive patterns achieve fast phenotypic optimization through allele frequency shifts at many loci, but do not necessarily lead to the fixation of any variation[13, 35]. Once selection pressure relaxes, the phenotype regresses toward the original (preselection) stat[52]. Secondly, no single genetic variation was shown to be either a necessary or sufficient condition for population breeding in this work.

In this study, we identified two genes *UTP25* and *FBRSL1* significantly associated with breast muscle weight. *FBRSL1* belongs to the Polycomb group (PcG) gene and is essential for many biological processes in mammals, including stem cell maintenance and differentiation[53, 54]. Our results showed that the expression level of *FBRSL1* in the breast muscle of Pekin duck Z2 line decreased with the increase of age. This may hint at *FBRSL1* was strongly selected to promote the development of Pekin duck breast muscle. *UTP25* (also named DIEXF in human) reportedly affects digestive organ expansion by regulating p53 pathway[55, 56]. However, the function of *UTP25* has not been characterized in animal muscles. *UTP25* was strongly selected in the continuous breeding process of Pekin duck Z2 line, and further investigation would be necessary to elucidate its functions.

It can be anticipated that CNVs may be more important hotspots to reveal selection-induced molecular changes in Pekin ducks. First of all, CNVs are widespread in domesticated animals, such as pigeon, sheep, pig, chicken, cattle, horse, and dog [57-66]. Moreover, the identified CNVs were largely found in genes that encode growth factors and receptors, and genes related to development and play a role mainly through duplication[67]. Secondly, CNV may have a role in rapid adaptation under strong selective pressure. This phenomenon was found in our experimental population that 81 copy number variations were identified in the Pekin duck genome under ten generations of high-intensity selection. Similar phenomena were found in experimental evolution

studies in microbes under nutrient limitation and multicellular systems[68-70]. Finally, and most importantly, there is evidence for CNVs under selection in domesticated species. During domestication, CNVs underlying domestication traits increase in frequency in the population in response to selection, and genomic signatures for selection can sometimes be detected associated with these CNVs[57, 71, 72]. In this study, we identified 2 duplication CNVRs were annotated in two genes EXOC4 and TRPA1 respectively. *EXOC4* may affect the development of breast muscle in Pekin ducks by affecting glucose transport and insulin synthesis. Studies have found that *EXOC4* was involved in insulin synthesis and glucose transport in skeletal muscle[73-76]. As a component of the exocyst complex, *EXOC4* is required for targeting of Glut4 to the plasma membrane by insulin[75]. Our results should that after ten years of artificial selection, the number of individuals with multiple copies of *EXOC4* gene increases in the population of Pekin ducks. We speculate that the duplication makes fold increase of Exoc4 proteins, and a large number of Exoc4 proteins facilitate glucose transport to cells. Since cells become more efficient at taking up glucose, the excess glucose can be converted to fat and amino acids and stored by the body. Therefore, this may be one of the factors affecting the change of breast muscle weight of Pekin ducks. However, this requires confirmation. There is solid evidence demonstrating that *TRPA1* is expressed throughout the mammalian body and has potential beneficial effects on systemic metabolism, including glucose metabolism[77]. Growing experimental evidence suggests that *TRPA1* plays an important role in weight gain, obesity and insulin secretion[42, 77-81]. In Pekin ducks, we did not observe the high expression level of *TRPA1* in breast muscle, but the body weight increased with the increase of breast muscle weight. Previously, our group has also demonstrated that there is a high genetic and phenotypic correlation between breast muscle weight and body weight in Pekin ducks (0.83 and 0.80)[33]. We surmise that *TRPA1* indirectly affects the breast muscle weight of Pekin ducks by affecting body weight.

In summary, our research enabled us to understand the genetic variation mechanism of farm animal genomes under intense artificial selection, and will provide useful information for the establishment of an efficient molecular breeding system for livestock.

Materials and Methods

1. Subject details and sampling

All duck samples for this study were collected from Pekin Duck Breeding Base, Changping District, Beijing. The Z2 line originated from the initial conserved population of Pekin duck in Beijing. Pekin duck Z2 line was selected as the research object, because it has many characteristics, including: **a** it is bred in closed group, with pure pedigree and clear genetic background; **b** the selection pressure of this line is constant, which is favorable for the accumulation of alleles gradually; **c** at the age of 6 weeks each generation, 15 male and 15 female ducks were randomly selected from a large population for slaughter test to measure their breast muscle weight and retain blood samples; **d** the breeding of all generations was completed in Pekin duck breeding Base, Changping District, Beijing, and the performance measurement was done in spring, resulting in little difference in environmental effect between generations. Furthermore, all ducks were kept in similar environment and had free access to water and feed pellets[33]. We measured the breast muscle weight of the whole population in vivo at the age of 6 weeks of each generation, and selected ducks with the heavier breast muscle weight as parents to produce the next generation. Roughly 750 individuals in each generation were retained for breeding, of which 35 % – 40 % retention rate for female ducks and 7 % – 8 % retention rate for male ducks. The inbreeding was strictly avoided by calculating the inbreeding coefficient of each generation. The breast muscle weight (BMW) trait was estimated by breast muscle volume (BMV), breast width (BB), keel length (KL) and breast muscle thickness (BMT) (**Fig. 2A, Supplementary Fig. S5**). The correlation equation between these traits was: $BMV=BB\times KL\times BMT$; $BMW = 0.6228\times BMV+17.042$ [19]. BB and KL were measured by vernier calipers, while BMT was measured by ultrasound scanning technology. The correlation parameters of BMW and BMV are real and reliable numerical results based on the measured breast muscle weight of years of slaughter experiments and fitted by linear regression equation model.

In this study, we collected the blood of 30 ducks and phenotypic data of each individual (15 males and 15 females per generation) in the first, fourth, seventh and tenth generations, respectively. A total of 120 duck samples were obtained (one sample was lost in the fourth generation), and a total of 119 samples were obtained finally.

In addition, we randomly selected an adult female Pekin duck from Pekin duck Z2 line to collect its blood for PacBio sequencing, and collected its breast muscle tissue for BioNano and Hi-C sequencing. Furthermore, phenotype and pedigree data from all samples were collated for subsequent analysis. All individuals are collected wing venous blood and rapidly frozen at -20 ℃. Phenol-chloroform method was used to extract blood DNA. The quality and quantity of the DNA were examined via Nanodrop and agarose gel electrophoresis. Then, Illumina HiSeq X Ten platform (Illumina HiSeq X Ten, RRID:SCR 016385) was used to sequence the paired-end sequencing libraries with an inserted fragment length of approximately 500 bp in 8 ×.

2. Reference genome assembly and annotation

We used the combined strategy of long-reads single-molecule sequencing (PacBio, RRID:SCR_017988)[82, 83], optical mapping (BioNano)[84, 85] and chromosome interaction mapping (Hi-C)[86] which improved contiguity and completeness relative to the BGI_duck_1.0[87]. We first used Canu (Canu, RRID:SCR_015880)) [88] (v1.7.1) to correct and trim the subreads of PacBio with the default parameters. Then we assembled the high-quality sequence obtained in the previous step into contigs and adjusted the “correctedErrorRate” parameter to 0.05. Pilon (Pilon, RRID:SCR_014731)[89] (v1.23) was used to polish the assembled contigs twice. Then scaffolds were assembled using Irys optical mapping data. We first adopted IrysSolve (BioNano Genomics) to assembled the raw BioNano data into optical map with default parameters. Next, the runBNG pipelines[90] (v1.02) were used to construct the scaffolds based on the overlapping information between optical map and PacBio contigs.

Then we adopted Hi-C technology to anchor the scaffolds near-chromosome level. We first used bowtie2 to align the clean Hi-C raw reads to scaffolds. We totally generated ~1.06 Gb pair-end reads and ~593.5 million were uniquely mapped to the scaffolds (Supplementary Table 10). After filtering out reads with low mapping, multiple hits, duplications, and singletons, only valid pairs were retained for subsequent analysis. After that, HiC-Pro (HiC-Pro, RRID:SCR_017643)[91] (v2.10.0) was used to construct interaction matrix for valid interaction pairs of ~371.6 million, and HiCPlotter[92] (v0.8.1) was used to draw interaction heat map. Then Juicer was used to align the clean Hi-C reads to the draft assembly, and then the extracted data were automatically generated into a nearly chromosomal length assembly using the 3d-DNA pipeline. The final draft was

corrected using PBJelly (PBJelly, RRID:SCR_012091)[93].

The genome assembly was annotated by the NCBI Eukaryotic Genome Annotation Pipeline[66], an automated pipeline that annotates genes, transcripts and proteins on draft and finished genome assemblies.

3. Variant calling and filtering

The raw reads from Illumina sequencing were filtered before downstream analyses by removing adapter sequences, contaminated reads, and low-quality reads. Then the reads were mapped to the assembly (GCA_003850225.1) with Burrows-Wheeler alignment(BWA aln, RRID:SCR_010910)[94] (v0.7.17-r1198) using the default parameters. SAMtools (Samtools, RRID:SCR_002105)[95] (v1.13-14) software was used to convert mapping results into the BAM format and to filter the unmapped and non-unique reads. The paired reads that were mapped to the exact same position on the reference genome were identified with MarkDuplicates in Picard[96] (Picard, RRID:SCR_006525) to avoid any influence on variant detection. After the comparative evaluation of the depth and coverage of the results, we used the HaplotypeCaller program of GATK (GATK, RRID:SCR_001876)[97] (v1.90) software to call SNP and InDel to ensure the accuracy. Meanwhile, this method can avoid the interference of false positive sites in the follow-up analysis. For SNPs and InDels, we restricted the variant form to biallelic variants by setting the option of GATK to "-T SelectVariants -SelectType SNP -- RestrictTallelesto Biallelic". For the total variants, we set the GATK option '-T SelectVariants -select " AF < 1.00 " ' to limit the allele frequency. We then filtered the output by using VCFtools VCFtools (VCFtools, RRID:SCR_001235)[98] (v0.1.14). SNPs that did not meet the following criteria were excluded: (1) 3 × < mean sequencing depth (over all included individuals) < 30 ×; (2) a minor allele frequency > 0.05 and a max allele frequency < 0.99; (3) maximum missing rate < 0.1; and (4) only two alleles.

We used the CNVcaller (CNVcaller, RRID:SCR_015752) [99] software to detect copy number variations across 119 individuals, and this method also took into account the depth of reads and pair relationship, so as to identify the copy number variation interval. We firstly specified a 1000bp sliding window and a 500 bp step to count the GC, repeat, and gap contents of each window in the reference genome to generate the reference genome database. Then we calculate the

absolute number of copies per window. Thirdly, we used the "CNV.Discovery.sh" script to detect the copy number variation region of the genomes with the parameter settings "-f 0.05 -h 5 -r 0.01 -p primaryCNVR -m mergeCNVR". Finally, we used the "Genotype.py" script to genotype the copy numbers of each sample and generate the VCF file.

4. Analysis of population genetic differences

Smartpca program of EIGENSOFT (Eigensoft, RRID:SCR_004965) [100] (v4.2) software was used for principal component analysis (PCA) of whole genome SNPs. We plotted the first two eigenvectors in two dimensions with our own R script for G1 and G4, G4 and G7, G7 and G10, as well as G1 and G10 populations, respectively.

For estimations of allele frequencies of single SNPs, we used VCFTools[98] (v0.1.14) to filter the raw sequencing data of G1 and G10 generations. The parameters are set to be "--max-missing 0.9 --maf 0.01 --min-meanDP 5 --max-meanDP 30". After filtering, 8,433,767 reliable SNPs were obtained for allele frequency estimation. The per-SNP absolute allele frequency difference (ΔAF) between G1 generation and G10 generation was then calculated using the formula: ΔAF= abs ($RefAF_{G10}$-$RefAF_{G1}$). We next binned SNPs by ΔAF in steps of 0.05 (i.e. ΔAF=0–0.05, 0.05–0.10, etc. until 0.95-1.00) and intersected these binned SNPs with coding exons, introns and UTRs.

5. Genome-wide association analysis of traits

A genome-wide association analysis (GWAS) was performed using a mixed linear model (MLM) of EMMAX[101] program using genome-wide SNP data and breast muscle weight of 119 individuals from the re-sequenced population. The analysis model was:

$y = Xb + G\mathrm{a} + e$.

Where, y is the phenotypic value (the breast muscle weight of per duck), X is the matrix corresponding to the fixed effect, and b is the fixed effect size. Fixed effects include sex effects. G is the genetic matrix corresponding to the population kinship, and e is the random residual. PCA was performed based on all SNPs and the top three components were set as fixed effects in the mixed model to correct for population stratification. We defined Bonferroni correction threshold of 0.01/N ($-\log_{10} P = 8.94$) to identify the significant loci of the GWAS results, where N was the number of whole-genome SNPs.

For the identified associated genes, we also referred to the Pekin duck Panoramic transcription

map (transcriptome data of all tissues in the three development stages) established by our previous study[46] to check whether these genes were expressed in the development stage of breast weight muscle, so as to further confirm that them did participate in the regulation of breast muscle development.

6. Identification of selected regions

We used the VCFtools [98](v0.1.14) software to calculate *F*st between G1 and G10 generations by selecting parameters of 10 kb windows with a 5 kb step size. We used selscan software[102](v1.2.0a) to calculate the XP-EHH values of G1 and G10 groups with the same parameter settings of 10 kb sliding windows and 5 kb step size, in which the G1 generation was taken as the reference group and the G10 generation as the query group. The overlapping regions of two windows with statistical values above the top 1 % of the quantile would be selected as preliminary candidate regions.

7. Linkage disequilibrium analysis

In addition, to further narrow the candidate interval, we also used the Haploview (Haploview, RRID:SCR_003076)[103] to analyze the linkage disequilibrium of the candidate region (Related to **Fig. 4**). We adopted the square correlation coefficient (r^2) as the coefficient to measure the linkage disequilibrium between the leader SNP and the surrounding SNPs. The parameters settings were "--ld-window 99999 --ld-window- kb 1000 --ld-window-r2 0 --r2". Then we integrated and plotted the LD data with GWAS results. (Related to **Supplementary Fig. S4**).

8. Identification of selected CNVRs

Based our new assembly, we used CNV caller[99], to identify genome-wide CNVs and CNVRs. To avoid false positives, two parameters, silhouette coefficient (silhouette coefficient > 0.7) and minor allele frequency (MAF>0.05), were adopted to filter the CNVRs obtained, and then we got 81 CNVRs with high credibility and accuracy on autosomes. Subsequently, we used the relative frequency difference (RFD)[104], to detect the copy number variations occurred on the Pekin duck genome during population differentiation. After that, we adopted the topmost 5% RFD value (absolute RFD value > 4.1) as the threshold to screen the potentially selected CNVRs.

Data Availability

The assembly and annotation of Pekin duck has been deposited in GenBank under the Bioproject accession code PRJNA496533 (accession No. RHJV01000000). All supporting data are available in the *GigaScience* GigaDB database [105].

Figure Legends

Fig. 1 Overview of the assembly quality and characteristics of the Pekin duck genome.
A Circular diagram depicting the characteristics of the GCA_003850225.1 assembly. The tracks from the outer to inner circles represent the following: chromosomes, gene density (window size of 200 kb), SNP density (window size of 200 kb), TE density (window size of 200 kb), CNV density (window size of 3 Mb) and GC content (%) (window size of 200 kb). **B, C** Tree maps of fragmentation differences between long-read (GCA_003850225.1) and short-read (BGI_duk1.0) Pekin duck genome assemblies. The size of each rectangle of each chromosome is scaled to that of the contig sequence. The larger and fewer the internal boxes are, the more contiguous the contigs. **D** Comparison of the sequence contig length distribution between long-read (GCA_003850225.1) and short-read (BGI_duk_1.0) Pekin duck genome assemblies. **E** Comparison of mapping rates when the Pekin duck population (119 birds from 10 generations) whole-genome resequencing data were mapped to GCA_003850225.1 and the short-read genome assembly (BGI_duk_1.0). A two-tailed paired t test was used for statistical assessment. ***$P < 0.001$.

Fig. 2 Phenotypic and population genetic structure variation over ten generations.
A Measurement of duck breast muscle volume (BMV) in vivo at 6 weeks of age. The three measured values were: ① breast breadth (BB), ② keel length (KL), and ③ breast muscle thickness (BMT). The weight of breast muscle was calculated based on the following formulas: BMV = BB×KL×BMT, and BMW = 0.6228×BMV+17.042. **B** Principal component analysis (PCA) of ten generations. The red circles represent first-generation (G1) individuals, the yellow circles represent fourth-generation (G4) individuals, the green circles represent seventh-generation (G7) individuals, and the blue circles represent tenth-generation (G10) individuals. **C** Changes in breast muscle weight in the Pekin duck Z2 line over ten generations.

Fig. 3 Δallele frequency analyses. The majority of SNPs showed low Δ*AF* values between the first and tenth generation ducks. The black line indicates the number of SNPs in nonoverlapping Δ*AF* bins (left y axis). The colored lines denote the M values (log2-fold changes) of the relative frequencies of SNPs in coding regions (yellow), UTRs (green), and introns (blue), according to Δ*AF* bins (right y-axis).

Fig. 4 Overlapping selection signals in the genomes of the first and tenth generations. A Manhattan plot of selected regions between the first generation (G1) and the tenth generation (G10). Pairwise fixation index values (*F*st) are calculated in 10-kb sliding windows and 5-kb steps. The significance threshold for *F*st is 0.103 (1%). **B** Manhattan plot of selected regions between the first generation (G1) and the tenth generation (G10). Cross-population extended haplotype homozygosity (XP-EHH) values were calculated in 10-kb sliding windows and 5-kb steps. The significance thresholds for XP-EHH were 1.475 and -1.480 (1%). **C** Manhattan plots for a GWAS of breast muscle weight. The gray horizontal dashed lines indicate the Bonferroni-corrected significance threshold of the GWAS ($-\log_{10} P = 8.94$), and the selection signals are indicated with a gray background. **D** Allele frequency trajectories of 22 SNPs. **E** The variation trend of genetic diversity in selected regions among generations (Chr3:0.18-0.44 Mb; Chr16:3.40-3.50 Mb). The indicated *P* values are based on one-way ANOVA. *** indicates $P < 0.001$, ** indicates $P < 0.01$, * indicates $P < 0.05$, and ns indicates that the *P* value was not significant. **F** Regions containing loci associated with breast muscle weight ranging from 0.18 to 0.44 Mb along chromosome 3 and 3.40 to 3.50 Mb along chromosome 16. All genotyped SNPs are color coded according to their pairwise LD with the leader SNP (Chr3:416692; Chr16:3467244) calculated by comparing the first generation (G1) and tenth-generation (G10) populations. SNPs are colored based on the strength of the LD values (r^2 values) considering the most strongly associated SNP and the other SNPs in the region. **G** The blue line diagrams refer to the fixation indexes (*F*st) on selected regions (Chr3:0.18-0.44 Mb; Chr16:3.40-3.50 Mb) between G1 and G10. *F*st values are calculated in 10-kb sliding windows in 5-kb steps. The selection signals that overlapped with characterized GWAS loci are indicated with a gray background. The green line diagrams refer to XP-EHH for selected regions (Chr3:0.18-0.44 Mb; Chr16:3.40-3.50 Mb) between G1 and G10. XP-EHH values are calculated in 10-kb sliding windows in 5-kb steps. The selection signals that

overlapped with characterized GWAS loci are indicated with a gray-blue background. **H** Schematic diagram showing the genes distributed within the candidate regions (Chr3:0.34-0.43 Mb and Chr16:3.46-3.49 Mb). **I** Associations between genotypes of two leader SNPs in candidate regions and breast muscle weight. Box plots indicate the median (centerline), 25th-75th percentiles (limits), and minimum and maximum values (whiskers). The indicated *P* values are based on one-way ANOVA. *** indicates $P < 0.001$, ** indicates $P < 0.01$, * indicates $P < 0.05$, and ns indicates that the *P* value was not significant.

Fig. 5 Genome-wide screening of selected copy number variations (CNVs) between the first and tenth generations. **A** The relative frequency difference (RFD) between the first generation (G1) and the tenth generation (G10) is plotted against the position on each of the autosomes. The two horizontal dashed lines indicate the genome-wide thresholds of selection signals, which showed the highest absolute RFD value of 5% (> 4.1). **B** Examples of copy number variant form of selected CNVs. The abscissa represents the selected copy number variation region and the ordinates on both sides represent standardized absolute copy numbers (0, homozygous deletion; 1, normal copy number, i.e. normal diploid; 0.5, loss of heterozygosity; 1.5, heterozygous repetition; 2, homozygous repetition; more than 2 denotes complex multicopy). CNV1 (Chr1:200.83-201.27 Mb) and CNV2 (Chr2:123.14-123.16 Mb) mainly consisted of multiple copies, and CNV3 (Chr4:54.81-54.82 Mb) was a deletion. **C** Schematic diagram showing the genes distributed within the candidate regions. **D** Frequency changes in candidate CNVs over 10 generations.

Table 1 Assembly statistics of the GCA_003850225.1 genome.

Additional Files

Supplemental Figure S1. The pipeline for multi-level chromosome assembly. Canu was used for constructing initial contigs. Then polishing was performed with Pilon using PacBio-only long reads. Hybrid scaffolding of the PacBio-corrected contigs and the BioNano-based consensus map was performed using the hybrid scaffolding module within runBNG software. The Hi-C sequencing data were first aligned to the assembled contigs/scaffolds using the Bowtie end-to-end algorithm, and then the assembled scaffolds were clustered, ordered, and directed into chromosome level using 3d-DNA. The final draft was corrected using PBJelly.

Supplemental Figure S2. Hi–C interactions among 29 chromosomes with a 40-kb resolution.

Supplemental Figure S3. BUSCO completeness assessment for the new assembly. In summary, it covered 93.3% (7780/8338) of complete BUSCO genes and 1.3% (111/8338) of fragmented BUSCO genes.

Supplemental Figure S4. Generational average phenotypic values of prime traits of Pekin duck Z2 line. The abscissa denotes the generation.

Supplemental Figure S5. Distribution of *F*st and XP-EHH of 10-kb windows size for whole-genome-wide variants between G1 and G10 generation. Bins of *F*st and XP-EHH are presented along the x axes. μ, mean; δ, standard deviation.

Supplemental Figure S6. The variation of allele frequency of putative regions over ten generations (correspond to Figure 2). (A) and (B) Red and green lines indicate the frequency variation of representative loci in selective regions of *F*st and XP-EHH, respectively (corresponding to a 1% significance level of *F*st in 10-kb sliding windows and a 1% significance level of XP-EHH in 10-kb sliding windows; and each line represents the top SNP of a sliding window). **(C)** Blue lines indicate the frequency variation of 22 SNPs reached the Bonferroni significance threshold of the GWAS ($-\log_{10} P > 8.94$).

Supplementary Figure S7. The variation trend of genetic diversity in potential selective regions among generations. This figure shows the trend of genetic diversity in the common selection signatures obtained by *F*st and XP-EHH tests across generations. The indicated *P* values are based on one-way ANOVA. ** indicates $P < 0.01$, * indicates $P < 0.05$ and ns indicates that *P* value was not significant.

Supplemental Figure S8. Linkage disequilibrium (LD) in two candidate regions of 10 generations. Each diamond contains a level of LD (r^2) between all SNP pairs.

Supplemental Figure S9. SNPs distribution in candidate regions.

Supplemental Figure S10. Expression levels of the *UTP25* and *FBRSL1* genes in different tissues of Pekin duck. (A) *UTP25* expression levels in Pekin duck tissues. **(B)** *FBRSL1* expression levels in Pekin duck tissues. The expression data were obtained from a global gene expression database for ducks generated from transcriptome analyses. The database lists the expression levels of all genes in breast muscle, skin, liver, fat (abdominal fat), brain, heart, kidney, lung, spleen, sternum and shank tissues at different developmental periods in Pekin ducks.

Supplemental Figure S11. Expression levels of the *UTP25* and *FBRSL1* genes at different developmental periods of Pekin duck Z2 line.

Supplemental Figure S12. Expression levels of the *EXOC4* and *TRPA1* genes in different tissues of Pekin duck. (A) *EXOC4* expression levels in Pekin duck tissues. **(B)** *TRPA1* expression levels in Pekin duck tissues. The expression data were obtained from a global gene expression database for ducks generated from transcriptome analyses. The database lists the expression levels of all genes in breast muscle, skin, liver, fat (abdominal fat), brain, heart, kidney, lung, spleen, sternum and shank tissues at different developmental periods in Pekin ducks.

Supplemental Table S1. Summary of sequencing data.

Supplemental Table S2. Summary of Hi-C reads mapping results.

Supplemental Table S3. Comparison of genome assemblies between the GCA_003850225.1 and BGI_duck_1.0.

Supplemental Table S4. Number of ducks used for 10 generations genome re-sequencing and mapping summary.

Supplemental Table S5. Genomic SNP statistics.

Supplemental Table S6. Distributions of SNP counts in the different delta allele frequency bins for coding sequences, introns and UTRs.

Supplemental Table S7. Overlapping regions between the analysis results of *F*st and XP-EHH. Pairwise fixation index values (*F*st) and cross-population extended haplotype homozygosity (XP-EHH) values are calculated in 10-kb windows sliding in 5-kb steps. The top 1% of the overlap regions between the analysis results of *F*st and XP-EHH were considered as

candidate regions.

Supplemental Table S8. Summary of putative regions on the genome. These regions sum to 2.06 Mb and ~2.14 Mb were annotated into 65 genes.

Supplemental Table S9. The significant SNPs of GWAS analyses. We performed GWAS analyses of breast muscle weight. The Bonferroni significance threshold of the GWAS was $-\log_{10} P > 8.94$. There were 22 SNPs that passed the Bonferroni significance threshold. These SNPs are located in non-coding sequences.

Supplemental Table S10. Putative CNVRs under artificial selection.

Supplemental Table S11. The copy number variation genotypes of three candidate CNVRs in each individual.

Abbreviations

BB: breast breadth; BMT: breast muscle thickness; BMV: breast muscle volume; BMW: breast muscle weight; bp: base pairs; BUSCO: Benchmarking Universal Single-Copy Orthologs; BWA: BurrowsWheeler Aligner; CNV: Copy number variation; *F*st: Fixation index; g: gram; G:generation; CNVR: copy number variation regions; Gb: gigabase pairs; GC: guanine-cytosine; GWAS: genome-wide association study; Hi-C: High-throughput Chromosome Conformation Capture; kb: kilobase pairs; KL: keel length; PCA: principle component analysis; LD: linkage disequilibrium; Mb: megabase pairs; MLM: mixed linear model; NCBI: National Center for Biotechnology Information; PacBio: Pacific Biosciences; RFD: relative frequency difference; SNP: single-nucleotide polymorphism; TE: transposable element; UTR: untranslated regions; XP-EHH: population extended haplotype homozygosity; ΔAF: allele frequency difference.

Competing interests

The authors have declared that no competing interests exist.

Ethics statement

All animals used in the study were treated following the guidelines for the experimental animals established by the Council of China Animal Welfare. Protocols of the experiments were approved by the Science Research Department of the Institute of Animal Sciences, Chinese Academy of Agricultural Sciences (CAAS) (Beijing, China).

Funding

This work was supported by grants from the National Natural Science Foundation of China (31972523), the Young Top-notch Talent Project of the National Ten Thousand Talent Program, the China Agriculture Research System of MOF and MARA (CARS-42-5) and the CAAS Innovation Team Project (ASTIP-IAS-9, CAAS-ZDRW202104).

Authors' contributions

Z.Zhou. and S.H. conceived the project and designed the research. managed the project. S.H., Z.Zhou., Z.G., J.Hu., M.Xie., W.Huang., Y.Zhang., and Q.Zhang. constructed the population. M.Xie., J.T., W.Huang., J.Hu., Y.Zhang., Z.G., G.Xing, W.F., Y.Xu, S.Liang. and D.Liu. collected the phenotype data. Y. Jiang., Z.Liu., M.Li., H.C., Z.Zhou. performed the genome assembly. S.Y., Z.Zhou., Z.Liu., D.Zhou. and P.Hua. performed bioinformatics analysis. S.Y., and Z.Zhou. wrote the manuscript.

Acknowledgments

We are grateful to all the members who involved in sample collection and processing.

References

1. Fuller RC, Baer CF and Travis J. How and When Selection Experiments Might Actually be Useful. Integr Comp Biol. 2005;45 3:391-404. doi:10.1093/icb/45.3.391.
2. Lou RN, Therkildsen NO and Messer PW. The Effects of Quantitative Trait Architecture on Detection Power in Short-Term Artificial Selection Experiments. G3 (Bethesda). 2020;10 9:3213-27. doi:10.1534/g3.120.401287.
3. Zhang H, Liang Q, Wang N, Wang Q, Leng L, Mao J, et al. Microevolutionary dynamics of chicken genomes under divergent selection for adiposity. iScience. 2020;23 6:101193. doi:10.1016/j.isci.2020.101193.
4. Zan Y, Sheng Z, Lillie M, Ronnegard L, Honaker CF, Siegel PB, et al. Artificial Selection Response due to Polygenic Adaptation from a Multilocus, Multiallelic Genetic Architecture. Molecular Biology and Evolution. 2017;34 10:2678-89. doi:10.1093/molbev/msx194.
5. Marks HL. Long-term selection for body weight in Japanese quail under different environments. Poultry Science. 1996;75 10:1198-203. doi:10.3382/ps.0751198.
6. Castro JP, Yancoskie MN, Marchini M, Belohlavy S, Hiramatsu L, Kučka M, et al. An integrative genomic analysis of the Longshanks selection experiment for longer limbs in mice. Elife. 2019;8 doi:10.7554/eLife.42014.
7. Kelly JK and Hughes KA. Pervasive Linked Selection and Intermediate-Frequency Alleles Are Implicated in an Evolve-and-Resequencing Experiment of Drosophila simulans. Genetics. 2019;211 3:943-61. doi:10.1534/genetics.118.301824.
8. Seabra SG, Fragata I, Antunes MA, Faria GS, Santos MA, Sousa VC, et al. Different Genomic Changes Underlie Adaptive Evolution in Populations of Contrasting History. Molecular Biology and Evolution. 2018;35 3:549-63. doi:10.1093/molbev/msx247.
9. Siegel PB. Selection for Body Weight at Eight Weeks of Age: 1. Short term Response and Heritabilities1. Poultry Science. 1962;41 3:954-62. doi:10.3382/ps.0410954.
10. Johansson AM, Pettersson ME, Siegel PB and Carlborg O. Genome-wide effects of long-term divergent selection. PLoS Genetics. 2010;6 11:e1001188. doi:10.1371/journal.pgen.1001188.
11. Dunnington EA and Siegel PB. Long-Term Divergent Selection for Eight-Week Body Weight in White Plymouth Rock Chickens. Poultry Science. 1996;75 10:1168-79. doi:10.3382/ps.0751168.
12. Lillie M, Sheng ZY, Honaker CF, Andersson L, Siegel PB and Carlborg Ö. Genomic signatures of 60 years of bidirectional selection for 8-week body weight in chickens. Poultry Science. 2018;97 3:781-90. doi:10.3382/ps/pex383.
13. Mette L, F HC, B SP and Örjan C. Bidirectional Selection for Body Weight on Standing Genetic Variation in a Chicken Model. G3·Genes|Genomes|Genetics. 2019;9 4 doi:10.25387/g3.7674281.
14. Wu X, Zhang Q, Xu S, Jin P, Luan P, Li Y, et al. Differential expression of six chicken genes associated with fatness traits in a divergently selected broiler population. Molecular and Cellular Probes. 2016;30 1:1-5. doi:10.1016/j.mcp.2015.12.003.
15. Wang HB, Li H, Wang QG, Zhang XY, Wang SZ, Wang YX, et al. Profiling of chicken adipose tissue gene expression by genome array. BMC Genomics. 2007;8:193. doi:10.1186/1471-2164-8-193.
16. Zhang H, Hu X, Wang Z, Zhang Y, Wang S, Wang N, et al. Selection signature analysis

implicates the PC1/PCSK1 region for chicken abdominal fat content. PLoS One. 2012;7 7:e40736-e. doi:10.1371/journal.pone.0040736.

17. Zhang H, Wang S-Z, Wang Z-P, Da Y, Wang N, Hu X-X, et al. A genome-wide scan of selective sweeps in two broiler chicken lines divergently selected for abdominal fat content. BMC Genomics. 2012;13 doi:10.1186/1471-2164-13-704.

18. Yang Z-M, Wang W, Du Z-Q, Cheng B, Wang Y, Yao J, et al. Expression Profiling of Preadipocyte MicroRNAs by Deep Sequencing on Chicken Lines Divergently Selected for Abdominal Fatness. PLos One. 2015;10 2 doi:10.1371/journal.pone.0117843.

19. Carneiro M, Rubin CJ, Di Palma F, Albert FW, Alfoldi J, Barrio AM, et al. Rabbit genome analysis reveals a polygenic basis for phenotypic change during domestication. Science. 2014;345 6200:1074-9. doi:10.1126/science.1253714.

20. Li X, Yang J, Shen M, Xie XL, Liu GJ, Xu YX, et al. Whole-genome resequencing of wild and domestic sheep identifies genes associated with morphological and agronomic traits. Nature Communications. 2020;11 1:2815. doi:10.1038/s41467-020-16485-1.

21. Zheng Z, Wang X, Li M, Li Y, Yang Z, Wang X, et al. The origin of domestication genes in goats. Science Advances. 2020;6:eaaz5216. doi:10.1126/sciadv.aaz5216.

22. Dong Y, Zhang X, Xie M, Arefnezhad B, Wang Z, Wang W, et al. Reference genome of wild goat (capra aegagrus) and sequencing of goat breeds provide insight into genic basis of goat domestication. BMC Genomics. 2015;16:431. doi:10.1186/s12864-015-1606-1.

23. Ai H, Fang X, Yang B, Huang Z, Chen H, Mao L, et al. Adaptation and possible ancient interspecies introgression in pigs identified by whole-genome sequencing. Nature Genetics. 2015;47 3:217-25. doi:10.1038/ng.3199.

24. Li J, Zhang J, Liu J, Zhou Y, Cai C, Xu L, et al. A new duck genome reveals conserved and convergently evolved chromosome architectures of birds and mammals. GigaScience. 2021;10 1:giaa142. doi:10.1093/gigascience/giaa142.

25. Zhang L, Hu J, Han X, Li J, Gao Y, Richards CM, et al. A high-quality apple genome assembly reveals the association of a retrotransposon and red fruit colour. Nature Communications. 2019;10 1:1494. doi:10.1038/s41467-019-09518-x.

26. Peace CP. DNA-informed breeding of rosaceous crops: promises, progress and prospects. Hortic Res. 2017;4:17006. doi:10.1038/hortres.2017.6.

27. Li M, Tian S, Jin L, Zhou G, Li Y, Zhang Y, et al. Genomic analyses identify distinct patterns of selection in domesticated pigs and Tibetan wild boars. Nature Genetics. 2013;45 12:1431-8. doi:10.1038/ng.2811.

28. Bickhart DM, Rosen BD, Koren S, Sayre BL, Hastie AR, Chan S, et al. Single-molecule sequencing and chromatin conformation capture enable de novo reference assembly of the domestic goat genome. Nature Genetics. 2017;49 4:643-50. doi:10.1038/ng.3802.

29. Zhu F, Yin Z-T, Wang Z, Smith J, Zhang F, Martin F, et al. Three chromosome-level duck genome assemblies provide insights into genomic variation during domestication. Nature Communications. 2021;12 1 doi:10.1038/s41467-021-26272-1.

30. Li M, Sun C, Xu N, Bian P, Tian X, Wang X, et al. De Novo Assembly of 20 Chicken Genomes Reveals the Undetectable Phenomenon for Thousands of Core Genes on Microchromosomes and Subtelomeric Regions. Molecular Biology and Evolution. 2022;39 4 doi:10.1093/molbev/msac066.

31. Wang K, Hu H, Tian Y, Li J, Scheben A, Zhang C, et al. The chicken pan-genome reveals gene

content variation and a promoter region deletion in IGF2BP1 affecting body size. Molecular Biology and Evolution. 2021; doi:10.1093/molbev/msab231.
32. Simao FA, Waterhouse RM, Ioannidis P, Kriventseva EV and Zdobnov EM. BUSCO: assessing genome assembly and annotation completeness with single-copy orthologs. Bioinformatics. 2015;31 19:3210-2. doi:10.1093/bioinformatics/btv351.
33. Xu Y, Hu J, Zhang Y, Guo Z, Huang W, Xie M, et al. Selection response and estimation of the genetic parameters for multidimensional measured breast meat yield related traits in a long-term breeding Pekin duck line. Asian-Australasian Journal Animal Science. 2018;31 10:1575-80. doi:10.5713/ajas.17.0837.
34. Reich D, Price AL and Patterson N. Principal component analysis of genetic data. Nature Genetics. 2008;40 5:491-2. doi:10.1038/ng0508-491.
35. Pritchard JK, Pickrell JK and Coop G. The genetics of human adaptation: hard sweeps, soft sweeps, and polygenic adaptation. Current Biology 2010;20 4:R208-15. doi:10.1016/j.cub.2009.11.055.
36. Wright S. The genetical strucyure of populations. Annals of Eugenics. 1949;15 1:323-54. doi:10.1111/j.1469-1809.1949.tb02451.x.
37. Sabeti PC, Varilly P, Fry B, Lohmueller J, Hostetter E, Cotsapas C, et al. Genome-wide detection and characterization of positive selection in human populations. Nature. 2007;449 7164:913-8. doi:10.1038/nature06250.
38. Cooper GM, Nickerson DA and Eichler EE. Mutational and selective effects on copy-number variants in the human genome. Nature Genetics. 2007;39 7 Suppl:S22-9. doi:10.1038/ng2054.
39. Bickhart DM, Hou Y, Schroeder SG, Alkan C, Cardone MF, Matukumalli LK, et al. Copy number variation of individual cattle genomes using next-generation sequencing. Genome Res. 2012;22 4:778-90. doi:10.1101/gr.133967.111.
40. Weischenfeldt J, Symmons O, Spitz F and Korbel JO. Phenotypic impact of genomic structural variation: insights from and for human disease. Nature Reviews Genetics. 2013;14 2:125-38. doi:10.1038/nrg3373.
41. Xu Y, Shi T, Cai H, Zhou Y, Lan X, Zhang C, et al. Associations of MYH3 gene copy number variations with transcriptional expression and growth traits in Chinese cattle. Gene. 2014;535 2:106-11. doi:10.1016/j.gene.2013.11.057.
42. Wu J, Wang J, Yue B, Xing-Tang F, Zhang C, Ma Y, et al. Research on association between variants and haplotypes of TRPV1 and TRPA1 genes with growth traits in three cattle breeds. Animal Biotechnology. 2019;30 3:202-11. doi:10.1080/10495398.2018.1470530.
43. Van Laere A-S, Nguyen M, Braunschweig M, Carine N, Collette C, Moreau L, et al. A regulatory mutation in IGF2 causes a major QTL effect on muscle growth in the pig. Nature. 2003;425:832-6. doi:10.1038/nature02064.
44. Clop A, Marcq F, Takeda H, Pirottin D, Tordoir X, Bibe B, et al. A mutation creating a potential illegitimate microRNA target site in the myostatin gene affects muscularity in sheep. Nature Genetics. 2006;38 7:813-8. doi:10.1038/ng1810.
45. Karim L, Takeda H, Lin L, Druet T, Arias JA, Baurain D, et al. Variants modulating the expression of a chromosome domain encompassing PLAG1 influence bovine stature. Nature Genetics. 2011;43 5:405-13. doi:10.1038/ng.814.
46. Zhou Z, Li M, Cheng H, Fan W, Yuan Z, Gao Q, et al. An intercross population study reveals genes associated with body size and plumage color in ducks. Nature Communications. 2018;9

1:2648. doi:10.1038/s41467-018-04868-4.

47. Carroll SB. Evo-devo and an expanding evolutionary synthesis: a genetic theory of morphological evolution. Cell. 2008;134 1:25-36. doi:10.1016/j.cell.2008.06.030.

48. Stern DL and Orgogozo V. The loci of evolution: how predictable is genetic evolution? Evolution. 2008;62 9:2155-77. doi:10.1111/j.1558-5646.2008.00450.x.

49. Wittkopp PJ and Kalay G. Cis-regulatory elements: molecular mechanisms and evolutionary processes underlying divergence. Nature Reviews Genetics. 2011;13 1:59-69. doi:10.1038/nrg3095.

50. Monroe JG, Srikant T, Carbonell-Bejerano P, Becker C, Lensink M, Exposito-Alonso M, et al. Mutation bias reflects natural selection in Arabidopsis thaliana. Nature. 2022; doi:10.1038/s41586-021-04269-6.

51. Nei M, Suzuki Y and Nozawa M. The neutral theory of molecular evolution in the genomic era. Annual Review of Genomics and Human Genetics 2010;11:265-89. doi:10.1146/annurev-genom-082908-150129.

52. Dunnington EA, Honaker CF, McGilliard ML and Siegel PB. Phenotypic responses of chickens to long-term, bidirectional selection for juvenile body weight--historical perspective. Poultry Science. 2013;92 7:1724-34. doi:10.3382/ps.2013-03069.

53. Bathla S, Rawat P, Baithalu R, Yadav ML, Naru J, Tiwari A, et al. Profiling of urinary proteins in Karan Fries cows reveals more than 1550 proteins. J Proteomics. 2015;127 Pt A:193-201. doi:10.1016/j.jprot.2015.05.026.

54. Chen Z, Yao Y, Ma P, Wang Q and Pan Y. Haplotype-based genome-wide association study identifies loci and candidate genes for milk yield in Holsteins. PLoS One. 2018;13 2:e0192695. doi:10.1371/journal.pone.0192695.

55. Zhang S-J, Liu C-J, Yu P, Zhong X, Chen J-Y, Yang X, et al. Evolutionary Interrogation of Human Biology in Well-Annotated Genomic Framework of Rhesus Macaque. Molecular Biology and Evolution. 2014;31 5:1309-24. doi:10.1093/molbev/msu084.

56. Chen J, Ruan H, Ng SM, Gao C, Soo HM, Wu W, et al. Loss of function of def selectively up-regulates Delta113p53 expression to arrest expansion growth of digestive organs in zebrafish. Genes & Development. 2005;19 23:2900-11.

57. Bruders R, Van Hollebeke H, Osborne EJ, Kronenberg Z, Maclary E, Yandell M, et al. A copy number variant is associated with a spectrum of pigmentation patterns in the rock pigeon (Columba livia). PLoS Genetics. 2020;16 5:e1008274. doi:10.1371/journal.pgen.1008274.

58. Ghosh S, Qu Z, Das PJ, Fang E, Juras R, Cothran EG, et al. Copy number variation in the horse genome. PLoS Genetics. 2014;10 10:e1004712. doi:10.1371/journal.pgen.1004712.

59. Guo Y, Gu X, Sheng Z, Wang Y, Luo C, Liu R, et al. A Complex Structural Variation on Chromosome 27 Leads to the Ectopic Expression of HOXB8 and the Muffs and Beard Phenotype in Chickens. PLoS Genetics. 2016;12 6:e1006071. doi:10.1371/journal.pgen.1006071.

60. Lee YL, Takeda H, Costa Monteiro Moreira G, Karim L, Mullaart E, Coppieters W, et al. A 12 kb multi-allelic copy number variation encompassing a GC gene enhancer is associated with mastitis resistance in dairy cattle. PLoS Genetics. 2021;17 7:e1009331. doi:10.1371/journal.pgen.1009331.

61. Qiu Y, Ding R, Zhuang Z, Wu J, Yang M, Zhou S, et al. Genome-wide detection of CNV regions and their potential association with growth and fatness traits in Duroc pigs. BMC Genomics.

2021;22 1:332. doi:10.1186/s12864-021-07654-7.
62. Liu M, Fang L, Liu S, Pan MG, Seroussi E, Cole JB, et al. Array CGH-based detection of CNV regions and their potential association with reproduction and other economic traits in Holsteins. BMC Genomics. 2019;20 1 doi:10.1186/s12864-019-5552-1.
63. Stafuzza NB, Silva RMO, Fragomeni BO, Masuda Y, Huang Y, Gray K, et al. A genome-wide single nucleotide polymorphism and copy number variation analysis for number of piglets born alive. BMC Genomics. 2019;20 1:321. doi:10.1186/s12864-019-5687-0.
64. Yuan C, Lu Z, Guo T, Yue Y, Wang X, Wang T, et al. A global analysis of CNVs in Chinese indigenous fine-wool sheep populations using whole-genome resequencing. BMC Genomics. 2021;22 1:78. doi:10.1186/s12864-021-07387-7.
65. Salmon Hillbertz NH, Isaksson M, Karlsson EK, Hellmen E, Pielberg GR, Savolainen P, et al. Duplication of FGF3, FGF4, FGF19 and ORAOV1 causes hair ridge and predisposition to dermoid sinus in Ridgeback dogs. Nature Genetics. 2007;39 11:1318-20. doi:10.1038/ng.2007.4.
66. Pruitt KD, Brown GR, Hiatt SM, Thibaud-Nissen F, Astashyn A, Ermolaeva O, et al. RefSeq: an update on mammalian reference sequences. Nucleic Acids Research. 2014;42 Database issue:D756-63. doi:10.1093/nar/gkt1114.
67. Lye ZN and Purugganan MD. Copy Number Variation in Domestication. Trends in Plant Science. 2019;24 4:352-65. doi:10.1016/j.tplants.2019.01.003.
68. DeBolt S. Copy number variation shapes genome diversity in Arabidopsis over immediate family generational scales. Genome Biology and Evolution. 2010;2:441-53. doi:10.1093/gbe/evq033.
69. Gresham D, Desai MM, Tucker CM, Jenq HT, Pai DA, Ward A, et al. The repertoire and dynamics of evolutionary adaptations to controlled nutrient-limited environments in yeast. PLoS Genet. 2008;4 12:e1000303. doi:10.1371/journal.pgen.1000303.
70. Farslow JC, Lipinski KJ, Packard LB, Edgley ML, Taylor J, Flibotte S, et al. Rapid Increase in frequency of gene copy-number variants during experimental evolution in Caenorhabditis elegans. BMC Genomics. 2015;16:1044. doi:10.1186/s12864-015-2253-2.
71. Axelsson E, Ratnakumar A, Arendt M-L, Maqbool K, Webster MT, Perloski M, et al. The genomic signature of dog domestication reveals adaptation to a starch-rich diet. Nature. 2013;495 7441:360-4. doi:10.1038/nature11837.
72. Reiter T, Jagoda E and Capellini TD. Dietary Variation and Evolution of Gene Copy Number among Dog Breeds. PLoS One. 2016;11 2:e0148899. doi:10.1371/journal.pone.0148899.
73. Ewart M-A, Clarke M, Kane S, Chamberlain LH and Gould GW. Evidence for a role of the exocyst in insulin-stimulated Glut4 trafficking in 3T3-L1 adipocytes. Journal of Biological Chemistry. 2005;280 5:3812-6. doi:10.1074/jbc.m409928200.
74. Fulcher F, Smith B, Russ M and Patel Y. Dual role for myosin II in GLUT4-mediated glucose uptake in 3T3-L1 adipocytes. Experimental Cell Research. 2008;314 17:3264-74. doi:10.1016/j.yexcr.2008.08.007.
75. Inoue M, Chang L, Hwang J, Chiang S-H and Saltiel AR. The exocyst complex is required for targeting of Glut4 to the plasma membrane by insulin. Nature. 2003;422 6932:629-33.
76. Kee AJ, Yang L, Lucas CA, Greenberg MJ, Martel N, Leong GM, et al. An actin filament population defined by the tropomyosin Tpm3.1 regulates glucose uptake. Traffic. 2015;16 7:691-711. doi:10.1111/tra.12282.
77. Derbenev AV and Zsombok A. Potential therapeutic value of TRPV1 and TRPA1 in diabetes

mellitus and obesity. Semin Immunopathol. 2016;38 3:397-406. doi:10.1007/s00281-015-0529-x.

78. Mahajan N, Khare P, Kondepudi KK and Bishnoi M. TRPA1: Pharmacology, natural activators and role in obesity prevention. European Journal of Pharmacology. 2021;912:174553. doi:10.1016/j.ejphar.2021.174553.

79. Kagawa Y, Ozaki-Masuzawa Y, Hosono T and Seki T. Garlic oil suppresses high-fat diet induced obesity in rats through the upregulation of UCP-1 and the enhancement of energy expenditure. Experimental and Therapeutic Medicine. 2020;19 2:1536-40. doi:10.3892/etm.2019.8386.

80. Khare P, Jagtap S, Jain Y, Baboota RK, Mangal P, Boparai RK, et al. Cinnamaldehyde supplementation prevents fasting-induced hyperphagia, lipid accumulation, and inflammation in high-fat diet-fed mice. Biofactors. 2016;42 2:201-11. doi:10.1002/biof.1265.

81. Kim MJ, Son HJ, Song SH, Jung M, Kim Y and Rhyu MR. The TRPA1 agonist, methyl syringate suppresses food intake and gastric emptying. PLoS One. 2013;8 8:e71603. doi:10.1371/journal.pone.0071603.

82. Eid J, Fehr A, Gray J, Luong K, Lyle J, Otto G, et al. Real-Time DNA sequencing from single polymerase molecules. Science. 2009;323 5910:133-8. doi:10.1126/science.1162986.

83. Gordon D, Huddleston J, Chaisson MJ, Hill CM, Kronenberg ZN, Munson KM, et al. Long-read sequence assembly of the gorilla genome. Science. 2016;352 6281:aae0344. doi:10.1126/science.aae0344.

84. Schwartz DC, Li X, Hernandez LI, Ramnarain SP, Huff EJ and Wang YK. Ordered restriction maps of Saccharomyces cerevisiae chromosomes constructed by optical mapping. Science. 1993;262 5130:110-4.

85. Hastie AR, Dong L, Smith A, Finklestein J, Lam ET, Huo N, et al. Rapid genome mapping in nanochannel arrays for highly complete and accurate de novo sequence assembly of the complex Aegilops tauschii genome. PLoS One. 2013;8 2:e55864. doi:10.1371/journal.pone.0055864.

86. Lieberman-Aiden E, van Berkum NL, Williams L, Imakaev M, Ragoczy T, Telling A, et al. Comprehensive mapping of long-range interactions reveals folding principles of the human genome. Science. 2009;326 5950:289-93. doi:10.1126/science.1181369.

87. Huang Y, Li Y, Burt DW, Chen H, Zhang Y, Qian W, et al. The duck genome and transcriptome provide insight into an avian influenza virus reservoir species. Nature Genetics. 2013;45 7:776-83. doi:10.1038/ng.2657.

88. Koren S, Walenz BP, Berlin K, Miller JR, Bergman NH and Phillippy AM. Canu: scalable and accurate long-read assembly via adaptivek-mer weighting and repeat separation. Genome Res. 2017;27 5:722-36. doi:10.1101/gr.215087.116.

89. Walker BJ, Abeel T, Shea T, Priest M, Abouelliel A, Sakthikumar S, et al. Pilon: an integrated tool for comprehensive microbial variant detection and genome assembly improvement. PLoS One. 2014;9 11:e112963. doi:10.1371/journal.pone.0112963.

90. Yuan Y, Bayer PE, Lee H-T and Edwards D. runBNG: a software package for BioNano genomic analysis on the command line. Bioinformatics. 2017;33 19:3107-9. doi:10.1093/bioinformatics/btx366.

91. Servant N, Varoquaux N, Lajoie BR, Viara E, Chen CJ, Vert JP, et al. HiC-Pro: an optimized and flexible pipeline for Hi-C data processing. Genome Biol. 2015;16:259. doi:10.1186/s13059-015-0831-x.

92. Akdemir KC and Chin L. HiCPlotter integrates genomic data with interaction matrices. Genome Biol. 2015;16:198. doi:10.1186/s13059-015-0767-1.

93. English AC, Richards S, Han Y, Wang M, Vee V, Qu J, et al. Mind the gap: upgrading genomes with Pacific Biosciences RS long-read sequencing technology. PLoS One. 2012;7 11:e47768. doi:10.1371/journal.pone.0047768.

94. Li H and Durbin R. Fast and accurate short read alignment with Burrows-Wheeler transform. Bioinformatics. 2009;25 14:1754-60. doi:10.1093/bioinformatics/btp324.

95. Danecek P, Bonfield JK, Liddle J. et al. Twelve years of SAMtools and BCFtools. Gigascience. 2021;10(2):giab008. doi: 10.1093/gigascience/giab008.

96. Picard Toolkit” 2019. Broad Institute, GitHub Repository. https://github.com/broadinstitute/picard.

97. McKenna A, Hanna M, Banks E, Sivachenko A, Cibulskis K, Kernytsky A, et al. The Genome Analysis Toolkit: a MapReduce framework for analyzing next-generation DNA sequencing data. Genome Res. 2010;20 9:1297-303. doi:10.1101/gr.107524.110.

98. Danecek P, Auton A, Abecasis G, Albers CA, Banks E, DePristo MA, et al. The variant call format and VCFtools. Bioinformatics. 2011;27 15:2156-8. doi:10.1093/bioinformatics/btr330.

99. Xihong W, Zhuqing Z, Yudong C, Ting C, Chao L, Weiwei F, et al. CNVcaller: highly efficient and widely applicable software for detecting copy number variations in large populations. Gigascience. 2017; 12:1-12. doi:10.1093/gigascience/gix115.

100. Price AL, Patterson NJ, Plenge RM, Weinblatt ME, Shadick NA and Reich D. Principal components analysis corrects for stratification in genome-wide association studies. Nature Genetics. 2006;38 8:904-9. doi:10.1038/ng1847.

101. Kang HM, Sul JH, Service SK, Zaitlen NA, Kong S-Y, Freimer NB, et al. Variance component model to account for sample structure in genome-wide association studies. Nature Genetics. 2010;42 4:348-54. doi:10.1038/ng.548.

102. Szpiech ZA and Hernandez RD. selscan: An Efficient Multithreaded Program to Perform EHH-Based Scans for Positive Selection. Molecular Biology and Evolution. 2014;31 10:2824-7. doi:10.1093/molbev/msu211.

103. Barrett JC, Fry B, Maller J and Daly MJ. Haploview: analysis and visualization of LD and haplotype maps. Bioinformatics. 2005;21 2:263-5. doi:10.1093/bioinformatics/bth457.

104. Zhou Z, Jiang Y, Wang Z, Gou Z, Lyu J, Li W, et al. Resequencing 302 wild and cultivated accessions identifies genes related to domestication and improvement in soybean. Nature Biotechnology. 2015;33 4:408-14. doi:10.1038/nbt.3096.

105. Yu S, Liu Z, Li M, Zhou D, Hua P, Cheng H, et al. Supporting data for "Resequencing of a Pekin duck breeding population provides insights into the genomic response to short-term artificial selection" GigaScience Database. 2023. http://dx.doi.org/10.5524/102361

Figure

Click here to access/download;Figure;Figure1.tif

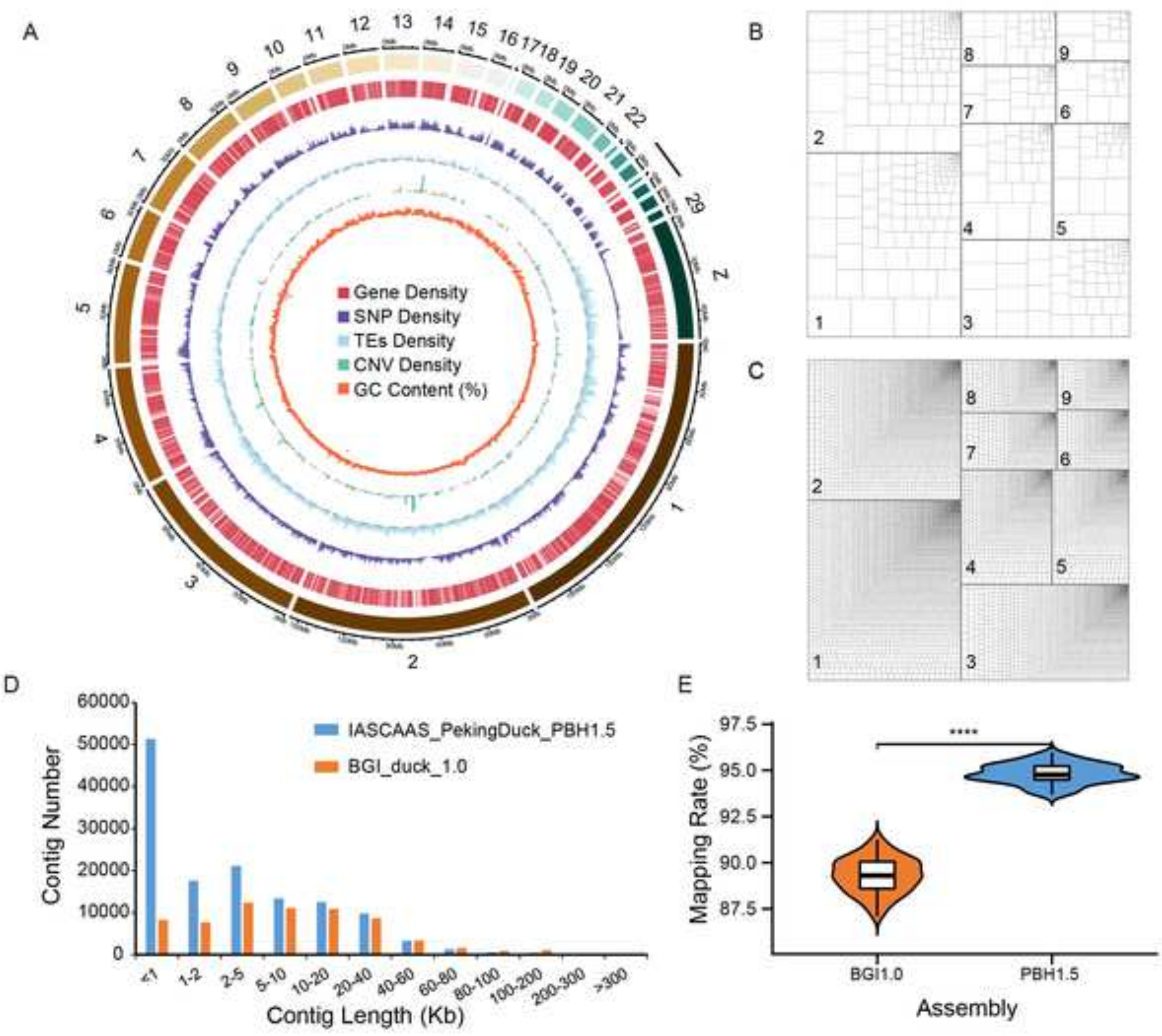

Click here to access/download;Figure;Figure2.tif

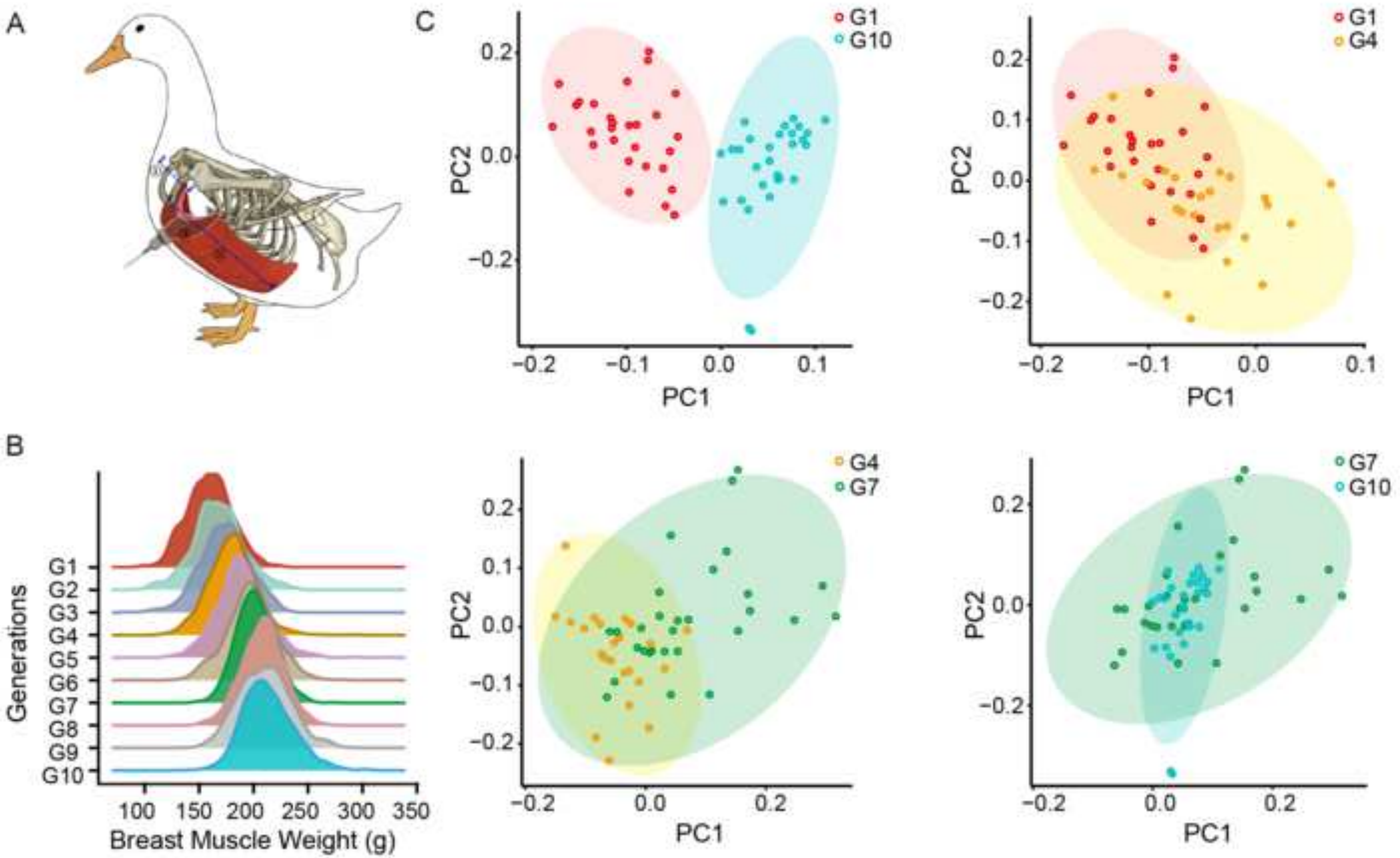

Click here to access/download;Figure;Figure3.tif

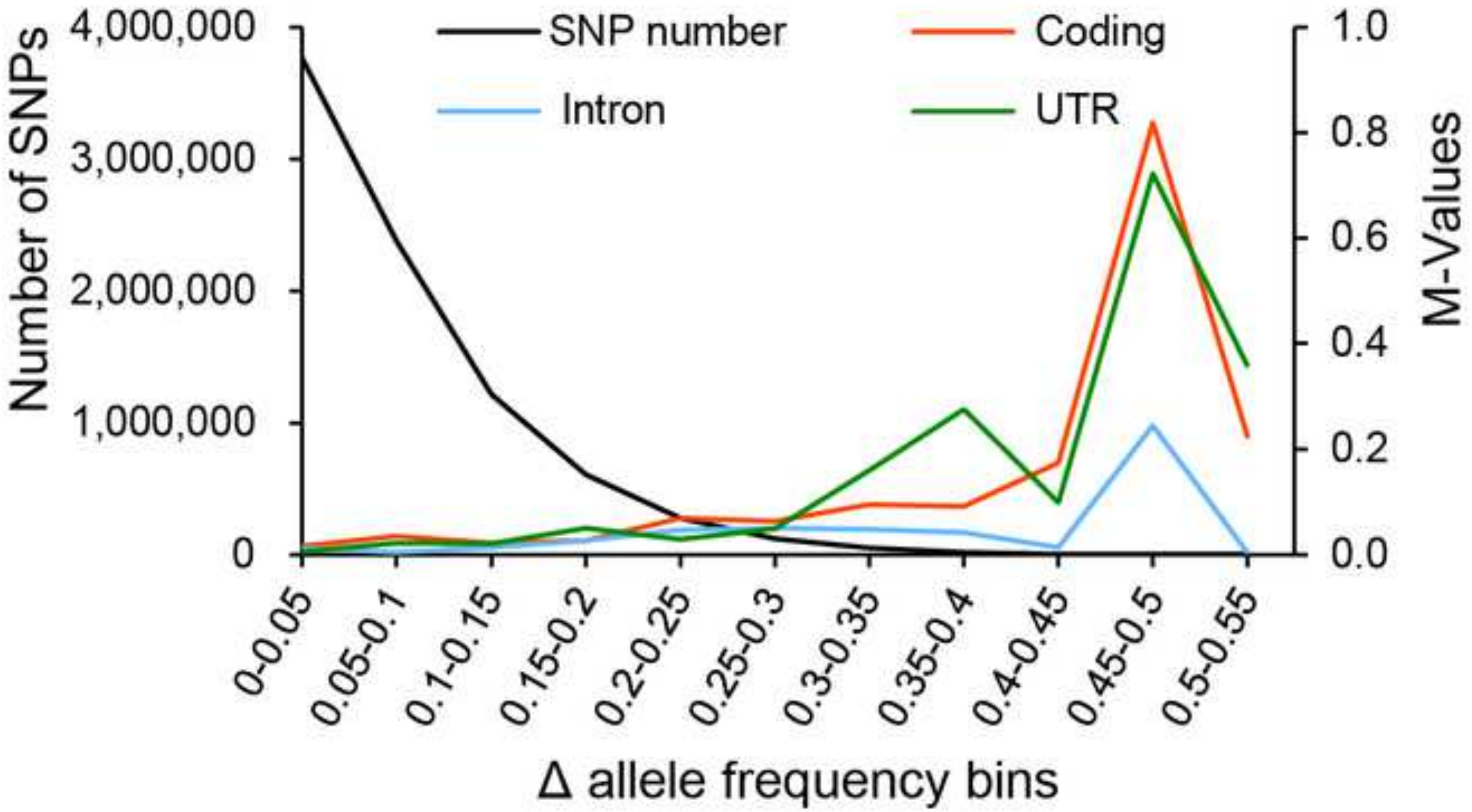

 Click here to access/download;Figure;Figure4.tif

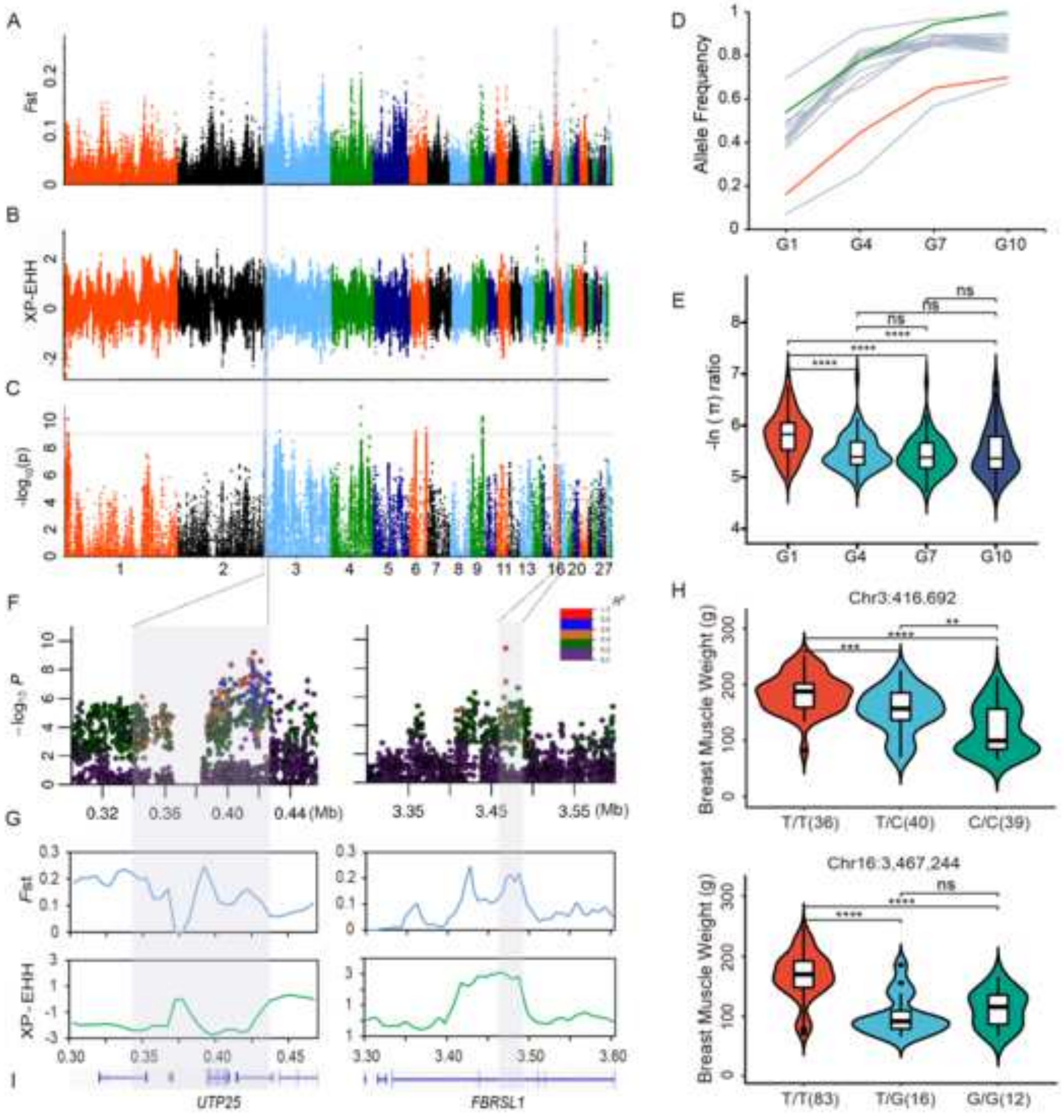

Click here to access/download;Figure;Figure5.Revised.tif

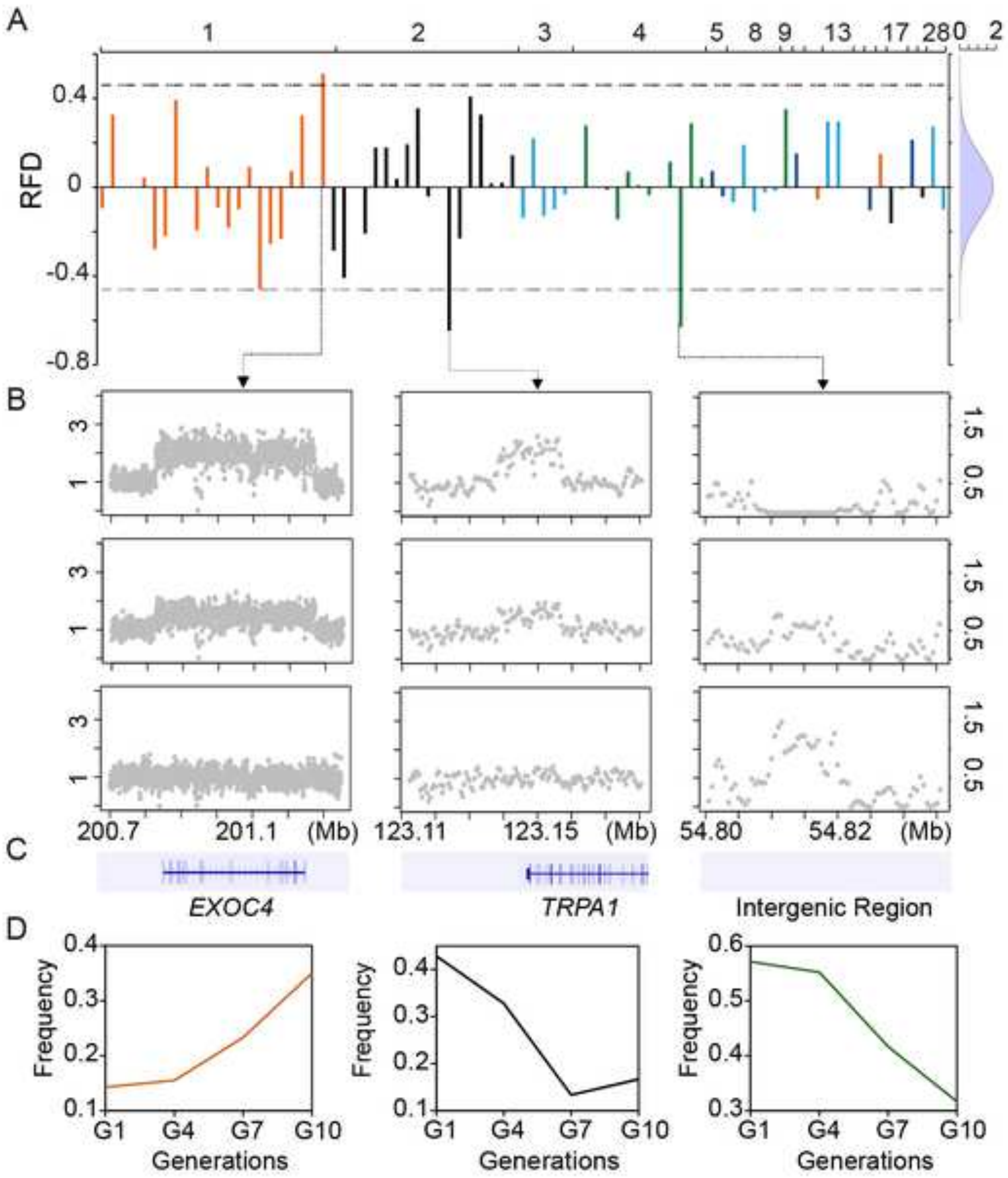

Click here to access/download
Supplementary Material
Response to Reviewer Comments.docx

Click here to access/download
Supplementary Material
Supplementary Figure.docx

Click here to access/download
Supplementary Material
SupplementaryTable.xlsx
