# Supplementary material for: Resequencing of a Pekin duck breeding population provides insights into the genomic response to short-term artificial selection
Source: Gigascience. 2023 Mar 27;12:giad016. doi: 10.1093/gigascience/giad016 (PMC10041536; doi:10.1093/gigascience/giad016)
Supplement: giad016_Supplemental_Figures_and_Tables [file giad016_supplemental_figures_and_tables.zip › Supplementary Figure.docx]

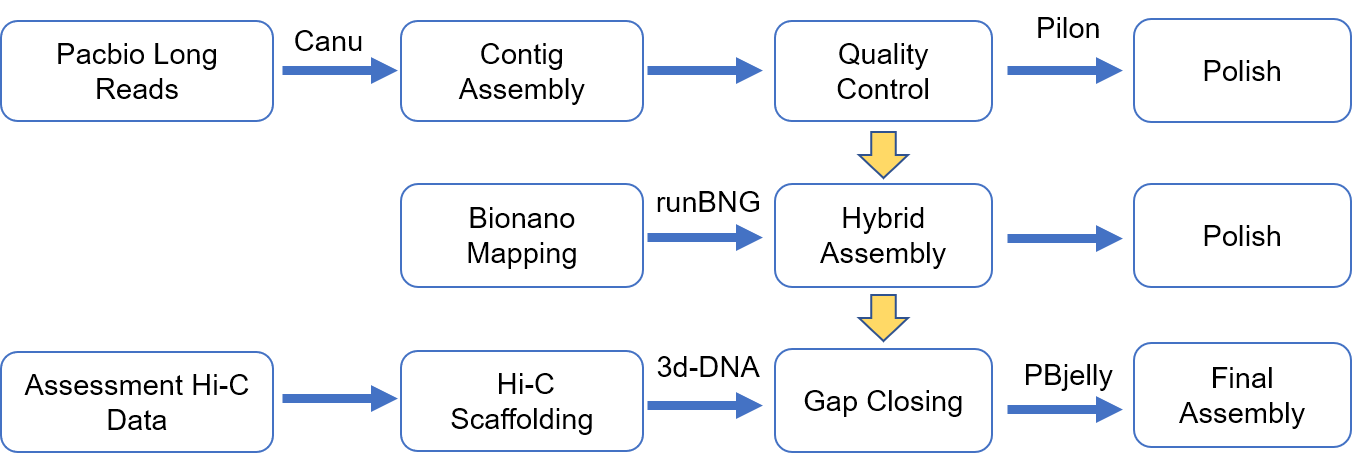


**Supplementary Figure S1.** **The pipeline for multi-level chromosome assembly.** Canu was used for constructing initial contigs. Then polishing was performed with Pilon using PacBio-only long reads. Hybrid scaffolding of the PacBio-corrected contigs and the BioNano-based consensus map was performed using the hybrid scaffolding module within runBNG software. The Hi-C sequencing data were first aligned to the assembled contigs/scaffolds using the Bowtie end-to-end algorithm, and then the assembled scaffolds were clustered, ordered, and directed into chromosome level using 3d-DNA. The final draft was corrected using PBJelly.


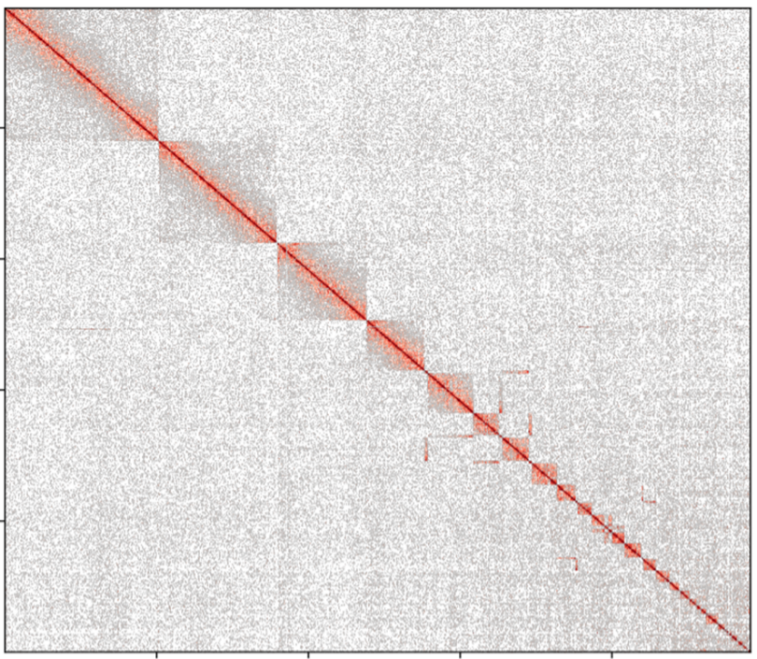


**Supplementary Figure** **S2. Hi–C interactions among 29 chromosomes with a 40-kb resolution.** Strong contacts are shown in red, and weak contacts are shown in grey.


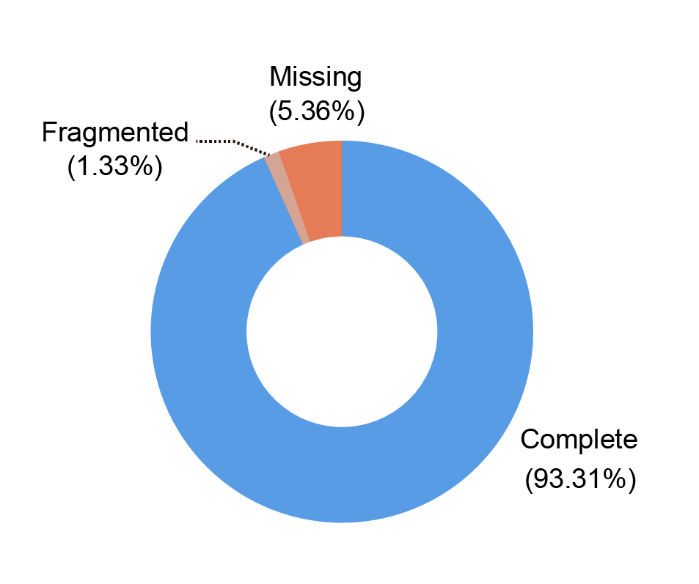


**Supplementary Figure S3. BUSCO completeness assessment for the new assembly.** In summary, it covered 93.3% (7780/8338) of complete BUSCO genes and 1.3% (111/8338) of fragmented BUSCO genes.


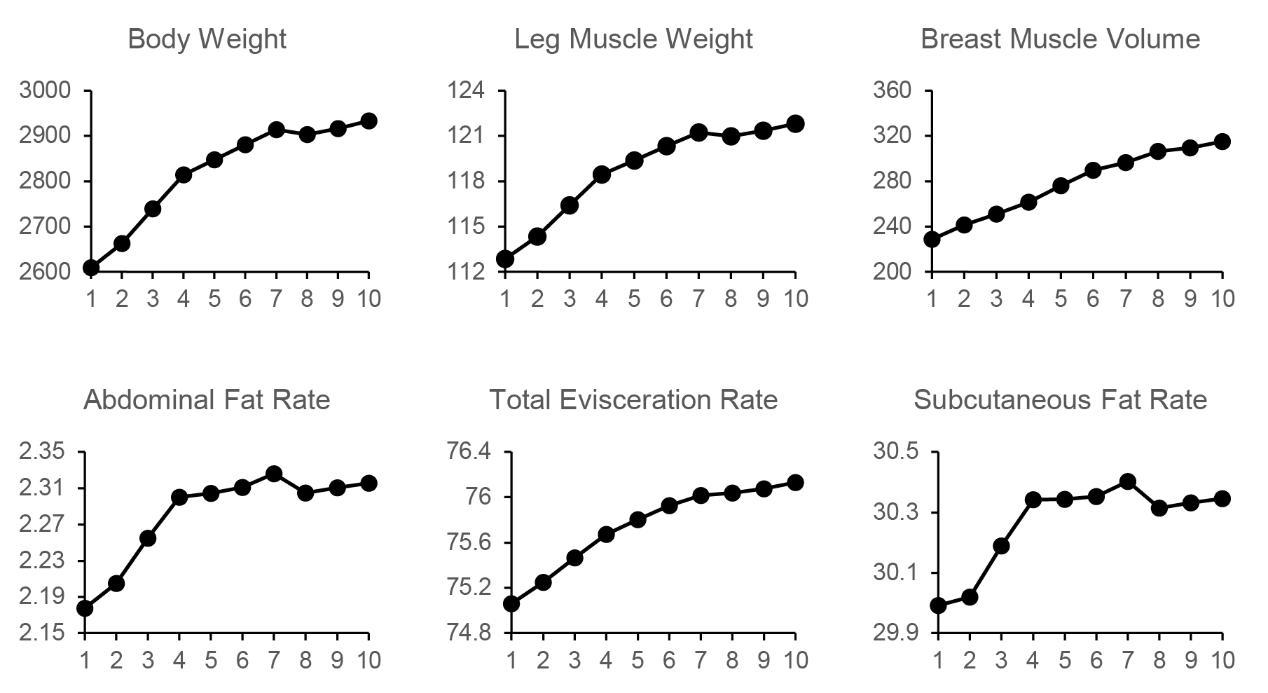


**Supplementary Figure S4.** **Generational average phenotypic values of prime traits of Pekin duck Z2 line.** The abscissa denotes the generation.


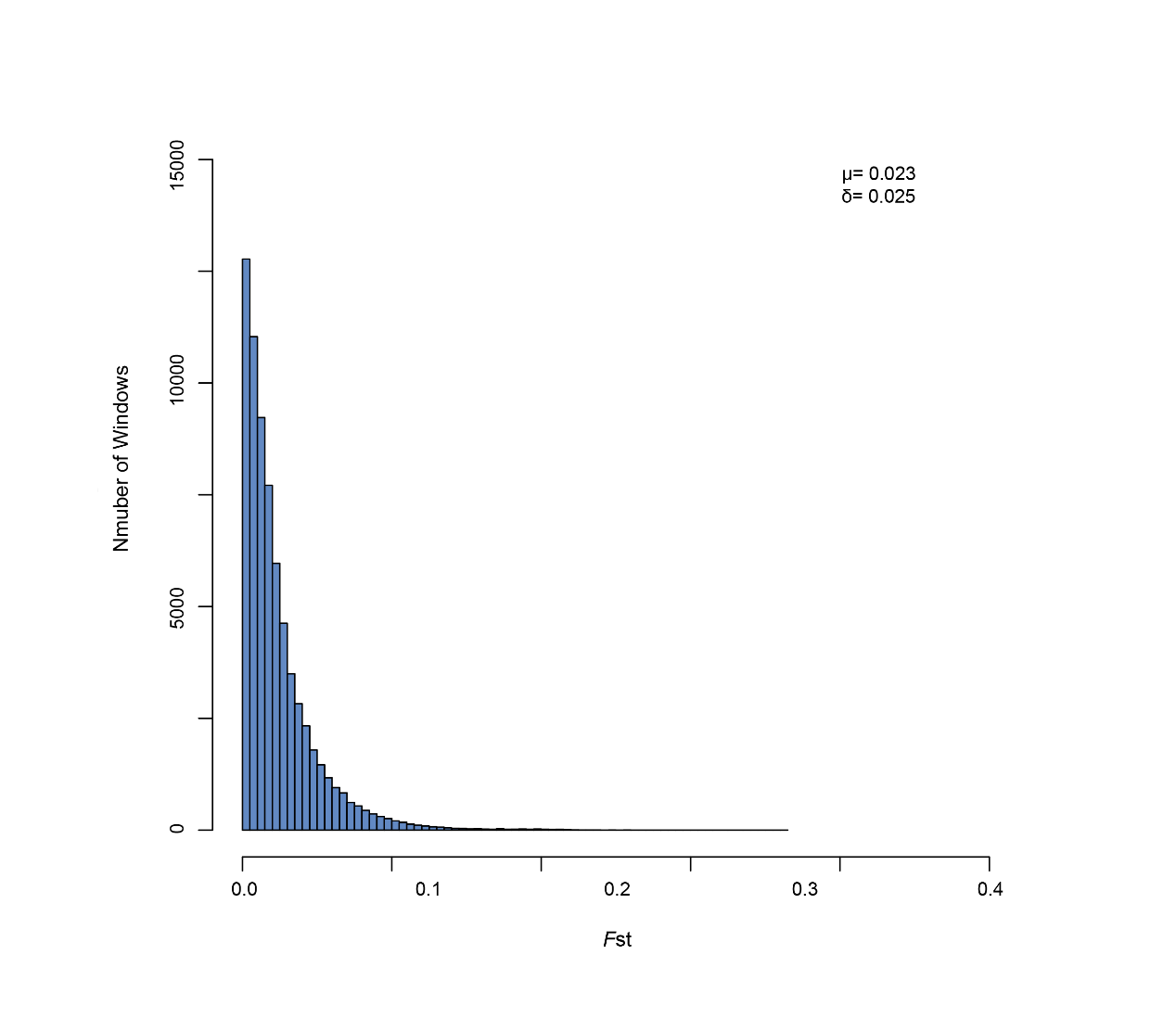


A


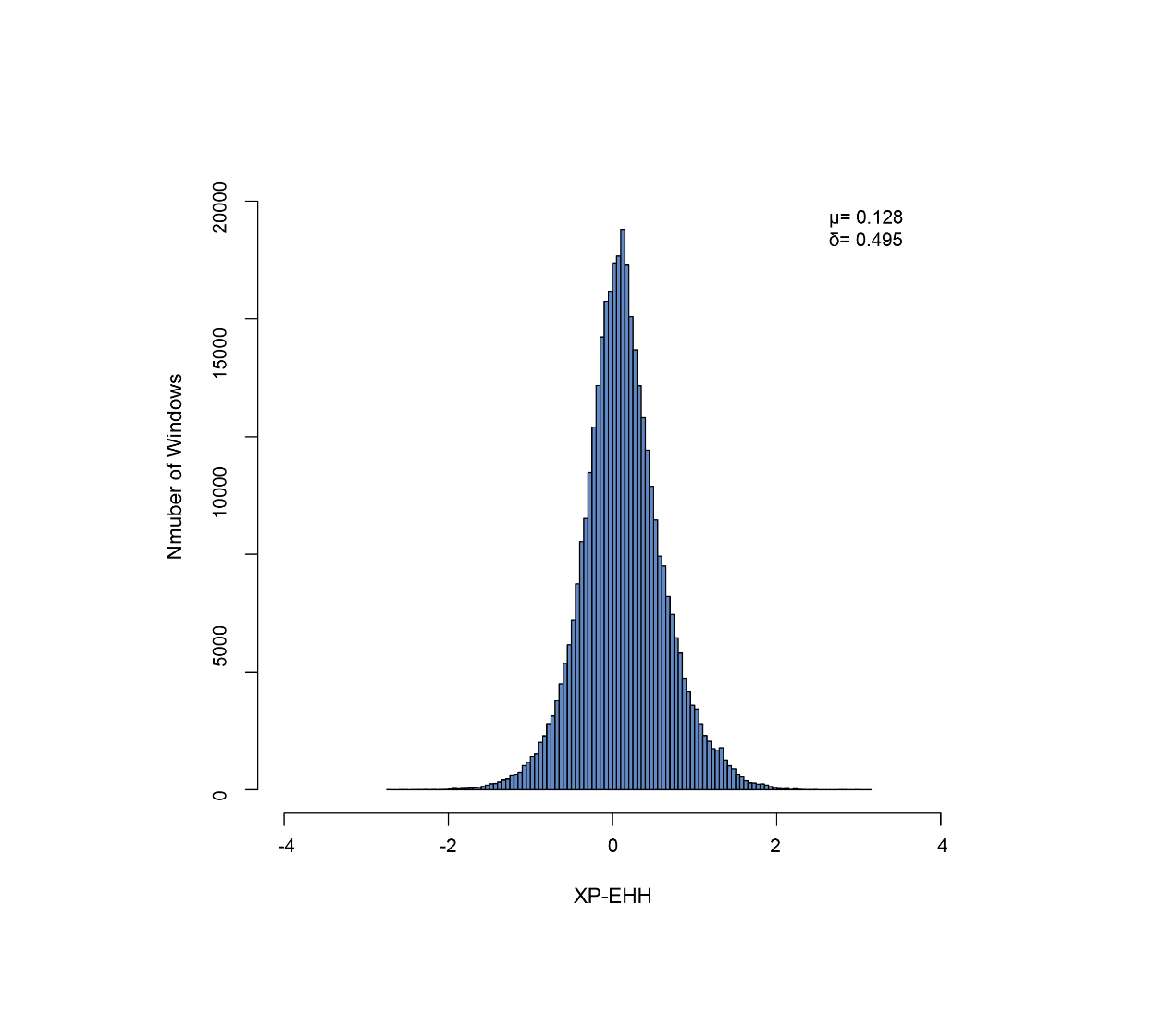


B

**Supplementary Figure S5. Distribution of *F*st and XP-EHH of 10-kb windows size for whole-genome-wide variants between G1 and G10 generation.** Bins of *F*st and XP-EHH are presented along the x axes. μ, mean; δ, standard deviation.

A

B

C


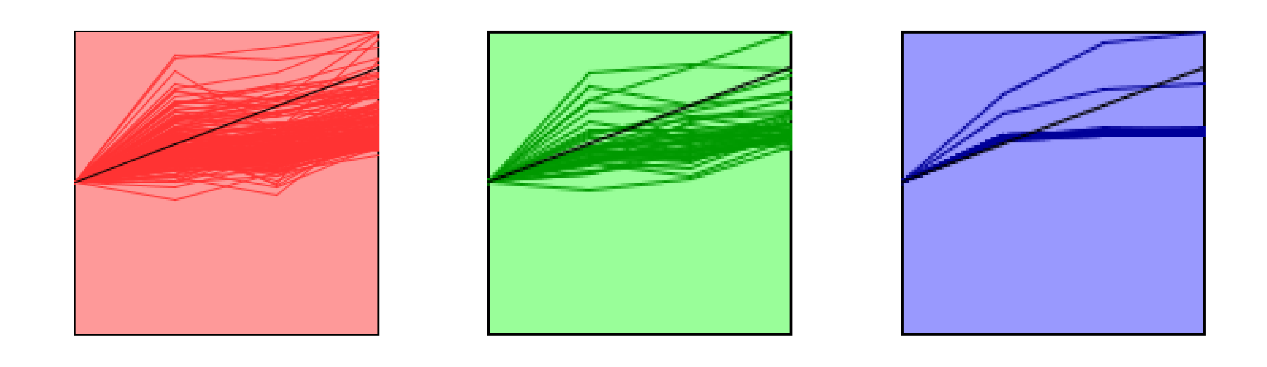


**Supplementary Figure S6. The variation of allele frequency of putative regions over ten generations (correspond to Figure 2). (A) and (B)** Red and green lines indicate the frequency variation of representative loci in selective regions of *F*st and XP-EHH, respectively (corresponding to a 1% significance level of *F*st in 10-kb sliding windows and a 1% significance level of XP-EHH in 10-kb sliding windows; and each line represents the top SNP of a sliding window). **(C)** Blue lines indicate the frequency variation of 22 SNPs reached the Bonferroni significance threshold of the GWAS (−log_10_ *P* = 8.94).


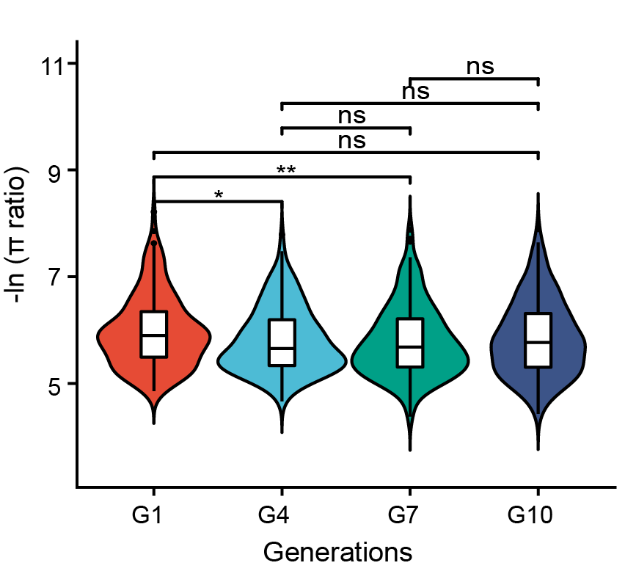


**Supplementary Figure S7. The variation trend of genetic diversity in potential selective regions among generations.** This figure shows the trend of genetic diversity in the common selection signatures obtained by *F*st and XP-EHH tests across generations. The indicated *P* values are based on one-way ANOVA. ** indicates *P* < 0.01, * indicates *P* < 0.05 and ns indicates that *P* value was not significant.


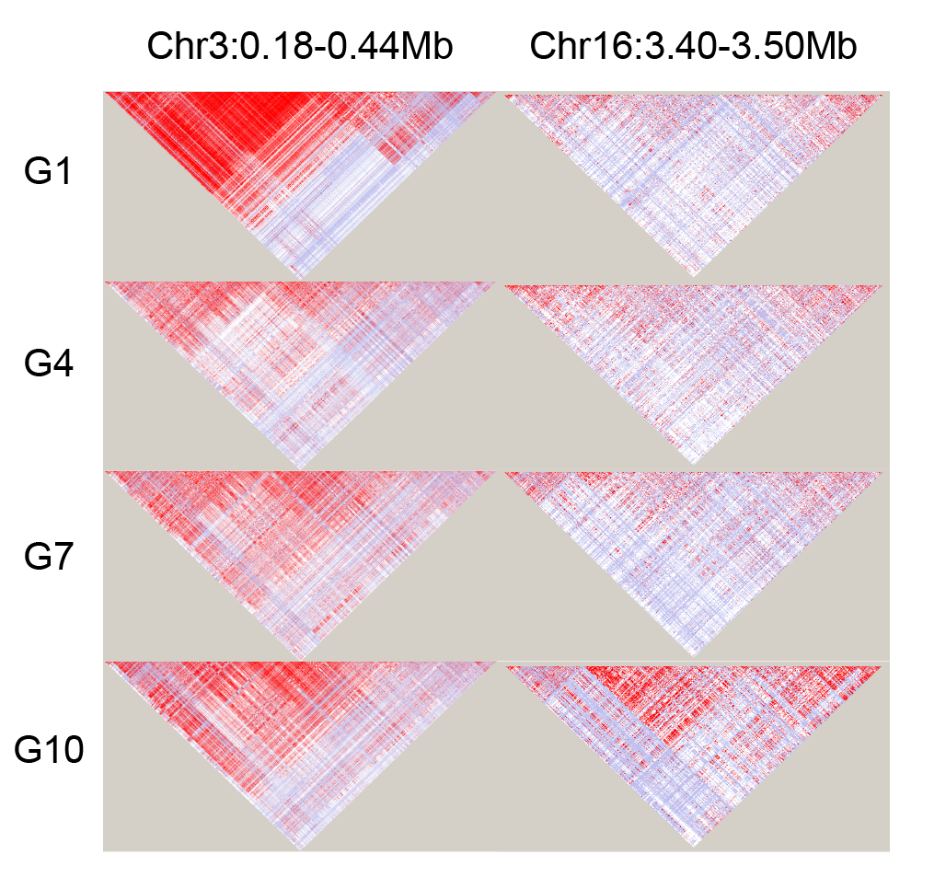


**Supplementary Figure S8.** **Linkage disequilibrium (LD) in two candidate regions of 10 generations.** Each diamond contains a level of LD (*r*^2^) between all SNP pairs.

**Supplementary Figure S9.** **SNPs distribution in candidate regions.**

A

B

**Supplementary Figure S10.** **Expression levels of the *UTP25* and *FBRSL1* genes in different tissues of Pekin duck. (A)** *FBRSL1* expression levels in Pekin duck tissues. **(B)** *UTP25* expression levels in Pekin duck tissues. The expression data were obtained from a global gene expression database for ducks generated from transcriptome analyses. The database lists the expression levels of all genes in breast muscle, skin, liver, fat (abdominal fat), brain, heart, kidney, lung, spleen, sternum and shank tissues at different developmental periods in Pekin ducks.

A

B

**Supplemental Figure S11. Expression levels of the *UTP25* and *FBRSL1* genes at different developmental periods of Pekin duck Z2 line.**

A

B

**Supplementary Figure S12. Expression levels of the *EXOC4* and *TRPA1* genes in different tissues of Pekin duck. (A)** *EXOC4* expression levels in Pekin duck tissues. **(B)** *TRPA1* expression levels in Pekin duck tissues. The expression data were obtained from a global gene expression database for ducks generated from transcriptome analyses. The database lists the expression levels of all genes in breast muscle, skin, liver, fat (abdominal fat), brain, heart, kidney, lung, spleen, sternum and shank tissues at different developmental periods in Pekin ducks.
