# Supplementary material for: Resequencing of a Pekin duck breeding population provides insights into the genomic response to short-term artificial selection
Source: Gigascience. 2023 Mar 27;12:giad016. doi: 10.1093/gigascience/giad016 (PMC10041536; doi:10.1093/gigascience/giad016)
Supplement: giad016_Response_to_Reviewer_Comments_Original_Submission [file giad016_response_to_reviewer_comments_original_submission.pdf]

Author's Response To Reviewer Comments

Close

Dear Dr. Zhang,
Thank you very much for handling our manuscript “Resequencing of a Pekin duck breeding population provides insights into the genomic response to short-term artificial selection” (GIGA-D-22-00268). We have carefully revised the manuscript according to the reviewers’ comments and your instructions. We appreciate all of the comments from the reviewers and have enclosed a revised manuscript as well as our point-by-point responses to the reviewers’ comments.
We believe that the revised manuscript has been greatly improved and hope it will meet the requirements of your journal. Thank you again for all of your assistance.

Sincerely yours,
Zhengkui Zhou and other coauthors

Reviewers’ Comments:
Reviewer #1:
1) In lines 35-38, The authors stated: "However, the genetic basis of short-term, intense artificial selection remains poorly understood. Although many studies have elucidated genetic mechanisms involved in intense artificial selection[4-8], most of them have not been genome-scale investigations." This might be true ten years ago, but there're many genomic-scale studies nowadays. Please cite important review papers instead and revise this paragraph.
Response: Thank you so much for your elaborative review and giving the precious advices. We have cited new important papers instead and rephrased this paragraph in line 35-42 of the revision into “Artificial selection experiments can help to understand the mechanism that allows populations to adapt to strong selection pressure[1, 2]. In combination with population-level genome sequencing, attempts have been made to identify alleles whose frequencies change systematically during selection experiments[3-5]. However, most studies were focused on lower organisms and were retrospective or used a single time point to characterize the dynamic changes in allele frequencies[1, 6-8]. Selecting a suitable domestic animal model for continuous high-intensity artificial selection trials may improve our understanding of the genetic basis of complex traits in domestic animals.”
References:

[1] Fuller RC, Baer CF and Travis J. How and When Selection Experiments Might Actually be Useful. Integr Comp Biol. 2005;45 3:391-404. doi:10.1093/icb/45.3.391.
[2] Lou RN, Therkildsen NO and Messer PW. The Effects of Quantitative Trait Architecture on Detection Power in Short-Term Artificial Selection Experiments. G3 (Bethesda). 2020;10 9:3213-27. doi:10.1534/g3.120.401287.
[3] Zhang H, Liang Q, Wang N, Wang Q, Leng L, Mao J, et al. Microevolutionary dynamics of chicken genomes under divergent selection for adiposity. iScience. 2020;23 6:101193. doi:10.1016/j.isci.2020.101193.
[4] Zan Y, Sheng Z, Lillie M, Ronnegard L, Honaker CF, Siegel PB, et al. Artificial Selection Response due to Polygenic Adaptation from a Multilocus, Multiallelic Genetic Architecture. Molecular Biology and Evolution. 2017;34 10:2678-89. doi:10.1093/molbev/msx194.
[5] Marks HL. Long-term selection for body weight in Japanese quail under different environments. Poult Sci. 1996;75 10:1198-203. doi:10.3382/ps.0751198.
[6] Castro JP, Yancoskie MN, Marchini M, Belohlavy S, Hiramatsu L, Kučka M, et al. An integrative genomic analysis of the Longshanks selection experiment for longer limbs in mice. Elife. 2019;8 doi:10.7554/eLife.42014.
[7] Kelly JK and Hughes KA. Pervasive Linked Selection and Intermediate-Frequency Alleles Are Implicated in an Evolve-and-Resequencing Experiment of Drosophila simulans. Genetics. 2019;211 3:943-61. doi:10.1534/genetics.118.301824.
[8] Seabra SG, Fragata I, Antunes MA, Faria GS, Santos MA, Sousa VC, et al. Different Genomic Changes

Underlie Adaptive Evolution in Populations of Contrasting History. Molecular Biology and Evolution. 2018;35 3:549-63. doi:10.1093/molbev/msx247.

2) In lines 59-65, those sentences were repeated almost exactly in the Data Description section. Please rephrase.
Response: We have rephrased these sentences in line 63-67 of the revision into "Herein, we adopted a Pekin duck pure line selected for breast muscle weight for 10 generations. The foundation stock was the local conserved population in Beijing, China. After 10 generations of high-intensity artificial selection, the breast muscle weight of the Pekin duck Z2 line increased from 80 g to 220 g. These data were investigated to elucidate the dynamic response patterns in the Pekin duck genome under artificial selection."

3) In line 84, "to assist in splicing the PacBio contigs into scaffolds", "splicing" should be the wrong word.
Response: Thanks for pointing out this error. We have modified the wrong words and expressions in line 84. The modified expression is "to link the PacBio contigs into scaffolds".

4) In lines 85-87, please explain why the scaffold numbers were greater after clustering by the Hi-C data than those using optical mapping while the scaffold N50 was significantly improved but contig N50 decreased.
Response: Thanks for the pertinent evaluations. The software we used for Hi-C data-assisted assembly was 3d-DNA.This software has a process of re-interrupting contigs during chromosome loading. Thus, more fragmented fragments were generated. After manual error correction and two iterations of error correction, the quality of scaffolds was improved. However, many fragments that were considered inaccurate and could not be corrected by chromatin interaction were also retained and counted. Thus, the contig N50 decreased.

5) In lines 104-106, the authors stated: "we conducted the resequencing of 30 individuals (15 males, 15 females) from G1 to G10 in intervals of 3 generations.", I wonder how the total number could add up to 119.
Response: Apologize for our unclear statement. Our sampling schedule was to collect the blood samples of 30 ducks and phenotypic data of each individual (15 males and 15 females per generation) in the first, fourth, seventh and tenth generations, respectively. A total of 120 duck samples were obtained (one sample was lost in the fourth generation), and a total of 119 samples were obtained finally. We have added the description of the sampling plan to the 'Materials and Methods' section, on lines 309-312 of the revision.

6) In lines 189-190, the authors stated: "In total, we identified 2 duplicated CNVRs and 1 deletion between the G1 and G10 populations (Fig. 5A and B)." This can be misinterpreted as these CNVRs only occurred in the G10 population but not in the G1 population. Please rephrase.
Response: Apologize for our unclear description about the result. We have rephrased the sentence in lines 195-196 into "In total, we identified 2 duplicated CNVRs and 1 deletion with significant allele frequency changes between the G1 and G10 populations (Fig. 5A and B)" make its meaning clearer and more specific.

7) In line 201, I doubt that "intelligence" should be applied to animals in this case, and I found that the interpretation in lines 202-205 was pretty unconvincing, especially while EXOC4 is also highly expressed in mammalian skeletal muscle. TRPA1 is also involved in skeletal muscle repair in mammals. However, the functions of these genes in muscle were not properly considered in the manuscript.
Response: Thank you for the comment, we agree with you. We have deleted the interpretation in lines 203-205 in the original manuscript and rephrased the sentence into "Combined with analysis with transcriptome data, showed that EXOC4 was widely expressed in various periods and tissues and the expression level in breast muscle, sebum and brain was higher than that in other tissues (Supplementary Fig. S12A)". We also added the gene function of EXOC4 and TRPA1 to the Discussion section in the revision. A new section on the Discussion is reproduced here: "In this study, we identified 2 duplication CNVRs were annotated in two genes EXOC4 and TRPA1 respectively. EXOC4 may affect the development of breast muscle in Pekin ducks by affecting glucose transport and insulin synthesis. Studies have found that EXOC4 was involved in insulin synthesis and glucose transport in skeletal muscle [73-76]. As a component of the exocyst complex, EXOC4 is required for targeting of Glut4 to the plasma membrane by insulin [75]. Our results should that after ten years of artificial selection, the number of individuals with multiple copies

of EXOC4 gene increases in the population of Pekin ducks. We speculate that the duplication makes fold increase of EXOC4 proteins, and a large number of EXOC4 proteins facilitate glucose transport to cells. Since cells become more efficient at taking up glucose, the excess glucose can be converted to fat and amino acids and stored by the body. Therefore, this may be one of the factors affecting the change of breast muscle weight of Pekin ducks. However, this requires confirmation. There is solid evidence demonstrating that TRPA1 is expressed throughout the mammalian body and has potential beneficial effects on systemic metabolism, including glucose metabolism [77]. Growing experimental evidence suggests that TRPA1 plays an important role in weight gain, obesity and insulin secretion [42, 77-81]. In Pekin ducks, we did not observe the high expression level of TRPA1 in breast muscle, but the body weight increased with the increase of breast muscle weight. Previously, our group has also demonstrated that there is a high genetic and phenotypic correlation between breast muscle weight and body weight in Pekin ducks (0.83 and 0.80) [33]. We surmise that TRPA1 indirectly affects the breast muscle weight of Pekin ducks by affecting body weight."

References:

[33] Xu Y, Hu J, Zhang Y, Guo Z, Huang W, Xie M, et al. Selection response and estimation of the genetic parameters for multidimensional measured breast meat yield related traits in a long-term breeding Pekin duck line. Asian-Australasian Journal Animal Science. 2018;31 10:1575-80. doi:10.5713/ajas.17.0837.

[42] Wu J, Wang J, Yue B, Xing-Tang F, Zhang C, Ma Y, et al. Research on association between variants and haplotypes of TRPV1 and TRPA1 genes with growth traits in three cattle breeds. Animal Biotechnology. 2019;30 3:202-11. doi:10.1080/10495398.2018.1470530.

[73] Ewart M-A, Clarke M, Kane S, Chamberlain LH and Gould GW. Evidence for a role of the exocyst in insulin-stimulated Glut4 trafficking in 3T3-L1 adipocytes. Journal of Biological Chemistry. 2005;280 5:3812-6. doi:10.1074/jbc.m409928200.

[74] Fulcher F, Smith B, Russ M and Patel Y. Dual role for myosin II in GLUT4-mediated glucose uptake in 3T3-L1 adipocytes. Experimental Cell Research. 2008;314 17:3264-74. doi:10.1016/j.yexcr.2008.08.007.

[75] Inoue M, Chang L, Hwang J, Chiang S-H and Saltiel AR. The exocyst complex is required for targeting of Glut4 to the plasma membrane by insulin. Nature. 2003;422 6932:629-33. doi:10.1038/nature01533.

[76] Kee AJ, Yang L, Lucas CA, Greenberg MJ, Martel N, Leong GM, et al. An actin filament population defined by the tropomyosin Tpm3.1 regulates glucose uptake. Traffic. 2015;16 7:691-711. doi:10.1111/tra.12282.

[77] Derbenev AV and Zsombok A. Potential therapeutic value of TRPV1 and TRPA1 in diabetes mellitus and obesity. Seminars In Immunopathology. 2016;38 3:397-406. doi:10.1007/s00281-015-0529-x.

[78] Mahajan N, Khare P, Kondepudi KK and Bishnoi M. TRPA1: Pharmacology, natural activators and role in obesity prevention. European Journal of Pharmacology. 2021;912:174553. doi:10.1016/j.ejphar.2021.174553.

[79] Kagawa Y, Ozaki-Masuzawa Y, Hosono T and Seki T. Garlic oil suppresses high-fat diet induced obesity in rats through the upregulation of UCP-1 and the enhancement of energy expenditure. Experimental and Therapeutic Medicine. 2020;19 2:1536-40. doi:10.3892/etm.2019.8386.

[80] Khare P, Jagtap S, Jain Y, Baboota RK, Mangal P, Boparai RK, et al. Cinnamaldehyde supplementation prevents fasting-induced hyperphagia, lipid accumulation, and inflammation in high-fat diet-fed mice. Biofactors. 2016;42 2:201-11. doi:10.1002/biof.1265.

[81] Kim MJ, Son HJ, Song SH, Jung M, Kim Y and Rhyu MR. The TRPA1 agonist, methyl syringate suppresses food intake and gastric emptying. PLoS One. 2013;8 8:e71603. doi:10.1371/journal.pone.0071603.

8) Potential functions of UTP25 and FBRSL1 in muscles were not considered and discussed.

Response: Thanks for your suggestion. We have adapted your advice and added some discussion about the potential functions of UTP25 and FBRSL1 in breast muscles in line 239-248 of the revision. Expression levels of the UTP25 and FBRSL1 genes at different developmental periods of Pekin duck Z2 line were tracked to increase the persuasiveness of the results and discussions. Thanks again for your comments and best wishes.

The new section in the Results is reproduced here: "We then tracked the expression levels of UTP25 and FBRSL1 genes in the breast muscles of different developmental periods of Pekin duck Z2 line. The results illustrated that the expression level of FBRSL1 decreased with the increase of days, while UTP25 was continuously expressed in breast muscle (Supplementary Fig. S11)."

The new section in the Discussion is reproduced here: "In this study, we identified two genes UTP25 and FBRSL1 significantly associated with breast muscle weight. FBRSL1 belongs to the Polycomb group (PcG)

gene and is essential for many biological processes in mammals, including stem cell maintenance and differentiation [53, 54]. Our results showed that the expression level of FBRSL1 in the breast muscle of Pekin duck Z2 line decreased with the increase of age. This may hint at FBRSL1 was strongly selected to promote the development of Pekin duck breast muscle. UTP25 (also named DIEXF in human) reportedly affects digestive organ expansion by regulating p53 pathway [55, 56]. However, the function of UTP25 has not been characterized in animal muscles. UTP25 was strongly selected in the continuous breeding process of Pekin duck Z2 line, and further investigation would be necessary to elucidate its functions."

References:

[53] Bathla S, Rawat P, Baithalu R, Yadav ML, Naru J, Tiwari A, et al. Profiling of urinary proteins in Karan Fries cows reveals more than 1550 proteins. J Proteomics. 2015;127 Pt A:193-201. doi:10.1016/j.jprot.2015.05.026.

[54] Chen Z, Yao Y, Ma P, Wang Q and Pan Y. Haplotype-based genome-wide association study identifies loci and candidate genes for milk yield in Holsteins. PLoS One. 2018;13 2:e0192695. doi:10.1371/journal.pone.0192695.

[55] Zhang S-J, Liu C-J, Yu P, Zhong X, Chen J-Y, Yang X, et al. Evolutionary Interrogation of Human Biology in Well-Annotated Genomic Framework of Rhesus Macaque. Molecular Biology and Evolution. 2014;31 5:1309-24. doi:10.1093/molbev/msu084.

[56] Chen J, Ruan H, Ng SM, Gao C, Soo HM, Wu W, et al. Loss of function of def selectively up-regulates Delta113p53 expression to arrest expansion growth of digestive organs in zebrafish. Genes & Development. 2005;19 23:2900-11.

Supplemental Figure S13. Expression levels of the UTP25 and FBRSL1 genes at different developmental periods of Pekin duck Z2 line.

9) Gene annotation methods were not described in the Methods section.

Response: The genome assembly was annotated by the NCBI Eukaryotic Genome Annotation Pipeline, an automated pipeline that annotates genes, transcripts and proteins on draft and finished genome assemblies. We did not manually correct the annotation of any gene. As suggested by the reviewer, we have described the pipeline used for the reference genome annotation in the Methods section of the revision and cited the relevant reference.

10) What are the three panels in Fig. 5B?

Response: Sorry for the confusing figure. Fig.5B is examples of copy number variation of selected CNV in different generations. We have added a detailed explanation of the meaning of Fig.5B to the Figure Legends section of the manuscript.

Reviewer #2:

1) While the Breast muscle yield of the Pekin line Z2 significantly changed from 80g to 220g over 10 generation of selection, details of this line with respect to its prime trait, 6Week's live weight, feed efficiency and other breast yield as a proportion of total meat-yield should be provided in the MS. It is obvious to detail that when breast yield improved more than 2 fold over the 10 generation's period, how good were the other meaty-cuts with reference to breast muscle yield. The Breeding process of Z2 (page 7) needs appropriate elaboration too.

Response: Thank you so much for your elaborative review and giving the precious advices, which help us to improve our manuscript. In the Supplementary Materials, we have added supplementary figures showing the changes of other prime traits of Pekin Duck Z2 line among generations. As evident from the supplementary figure that the live weight, breast muscle volume, leg muscle weight, subcutaneous fat rate, abdominal fat rate and total evisceration rate increased gradually among the ten generations. The supplementary figure is reproduced here:

Supplementary Figure S4. Generational average phenotypic values of prime traits of Pekin duck Z2 line. The abscissa denotes the generation.
The breeding process of Z2 line was described in detail in the section of Materials and Methods. Please refer to the 287-308 lines of the revision. We reproduce the detailed description here: “Z2 line originated from the initial conserved population of Pekin duck in Beijing. Pekin duck Z2 line was selected as the research object, because it has many characteristics, including: a it is bred in closed group, with pure pedigree and clear genetic background; b the selection pressure of this line is constant, which is favorable for the accumulation of alleles gradually; c at the age of 6 weeks each generation, 15 male and 15 female ducks were randomly selected from a large population for slaughter test to measure their breast muscle weight and retain blood samples; d the breeding of all generations was completed in Pekin duck breeding Base, Changping District, Beijing, and the performance measurement was done in spring, resulting in little difference in environmental effect between generations. Besides that, all ducks were kept in similar environment and had free access to water and feed pellets [33]. We measured the breast muscle weight of the whole population in vivo at the age of 6 weeks of each generation, and selected ducks with the heavier breast muscle weight as parents to produce the next generation. About 750 individuals in each generation were retained for breeding, of which 35 % – 40 % retention rate for female ducks and 7 % – 8 % retention rate for male ducks. The inbreeding was strictly avoided by calculating the inbreeding coefficient of each generation. The breast muscle weight (BMW) trait was estimated by breast muscle volume (BMV), breast width (BB), keel length (KL) and breast muscle thickness (BMT) (Fig. 2A, Supplementary Fig. S5). The correlation equation between these traits was: $BMV=BB\times KL\times BMT$; $BMW = 0.6228\times BMV+17.042$ [19]. BB and KL were measured by vernier calipers, while BMT was measured by ultrasound scanning technology. The correlation parameters of BMW and BMV are real and reliable numerical results based on the measured breast muscle weight of years of slaughter experiments and fitted by linear regression equation model.”

2) While most narration on Re-sequencing of the generation specific DNA samples are relevant, making a lot of descriptions by including many digital large images appear redundant, and therefore, inclusion of too many images to drive home the points need to be looked at thoroughly. Why need more than 2 or 3 images, when the facts can be well-cited as texts!
Response: Sorry for our redundant information. We have deleted and merged the redundant attached images.

3) For a study of such elaborate dimension, the discussion appears little too condensed, and therefore, be optimized to discuss, how the last 10 generations of selection brought so much of perceptible changes at molecular level, which were concerning more to non-coding regions than coding sequences. Discussions also may be beefed up citing experience of other genome studies (even beyond chickens and turkeys, to other food-purpose livestocks), for why CNV regions could be more important hotspots to reveal selection-induced molecular changes in Pekin ducks.
Response: Thank you for your valuable advice. We have optimized the discussion section based on your suggestions. The new section in the Discussion is reproduced here: “It can be anticipated that CNVs may be more important hotspots to reveal selection-induced molecular changes in Pekin ducks. First of all, CNVs are widespread in domesticated animals, such as pigeon, sheep, pig, chicken, cattle, horse, and dog [57-66]. Moreover, the identified CNVs were largely found in genes that encode growth factors and receptors, and genes related to development and play a role mainly through duplication [67]. Secondly, CNV may have a role in rapid adaptation under strong selective pressure. This phenomenon was found in our experimental population that 81 copy number variations were identified in the Pekin duck genome under ten generations of high-intensity selection. Similar phenomena were found in experimental evolution studies in microbes under nutrient limitation and multicellular systems [68-70]. Finally, and most importantly, there is evidence for CNVs under selection in domesticated species. During domestication, CNVs underlying domestication traits increase in frequency in the population in response to selection, and genomic signatures for selection can sometimes be detected associated with these CNVs [57, 71, 72].”
References:
[57] Bruders R, Van Hollebeke H, Osborne EJ, Kronenberg Z, Maclary E, Yandell M, et al. A copy number variant is associated with a spectrum of pigmentation patterns in the rock pigeon (Columba livia). PLoS Genetics. 2020;16 5:e1008274. doi:10.1371/journal.pgen.1008274.
[58] Ghosh S, Qu Z, Das PJ, Fang E, Juras R, Cothran EG, et al. Copy number variation in the horse genome. PLoS Genetics. 2014;10 10:e1004712. doi:10.1371/journal.pgen.1004712.

[59] Guo Y, Gu X, Sheng Z, Wang Y, Luo C, Liu R, et al. A Complex Structural Variation on Chromosome 27 Leads to the Ectopic Expression of HOXB8 and the Muffs and Beard Phenotype in Chickens. PLoS Genetics. 2016;12 6:e1006071. doi:10.1371/journal.pgen.1006071.
[60] Lee YL, Takeda H, Costa Monteiro Moreira G, Karim L, Mullaart E, Coppieters W, et al. A 12 kb multi-allelic copy number variation encompassing a GC gene enhancer is associated with mastitis resistance in dairy cattle. PLoS Genetics. 2021;17 7:e1009331. doi:10.1371/journal.pgen.1009331.
[61] Qiu Y, Ding R, Zhuang Z, Wu J, Yang M, Zhou S, et al. Genome-wide detection of CNV regions and their potential association with growth and fatness traits in Duroc pigs. BMC Genomics. 2021;22 1:332. doi:10.1186/s12864-021-07654-7.
[62] Liu M, Fang L, Liu S, Pan MG, Seroussi E, Cole JB, et al. Array CGH-based detection of CNV regions and their potential association with reproduction and other economic traits in Holsteins. BMC Genomics. 2019;20 1 doi:10.1186/s12864-019-5552-1.
[63] Stafuzza NB, Silva RMO, Fragomeni BO, Masuda Y, Huang Y, Gray K, et al. A genome-wide single nucleotide polymorphism and copy number variation analysis for number of piglets born alive. BMC Genomics. 2019;20 1:321. doi:10.1186/s12864-019-5687-0.
[64] Yuan C, Lu Z, Guo T, Yue Y, Wang X, Wang T, et al. A global analysis of CNVs in Chinese indigenous fine-wool sheep populations using whole-genome resequencing. BMC Genomics. 2021;22 1:78. doi:10.1186/s12864-021-07387-7.
[65] Salmon Hillbertz NH, Isaksson M, Karlsson EK, Hellmen E, Pielberg GR, Savolainen P, et al. Duplication of FGF3, FGF4, FGF19 and ORAOV1 causes hair ridge and predisposition to dermoid sinus in Ridgeback dogs. Nature Genetics. 2007;39 11:1318-20. doi:10.1038/ng.2007.4.
[66] Pruitt KD, Brown GR, Hiatt SM, Thibaud-Nissen F, Astashyn A, Ermolaeva O, et al. RefSeq: an update on mammalian reference sequences. Nucleic Acids Research. 2014;42 Database issue:D756-63. doi:10.1093/nar/gkt1114.
[67] Lye ZN and Purugganan MD. Copy Number Variation in Domestication. Trends in Plant Science. 2019;24 4:352-65. doi:10.1016/j.tplants.2019.01.003.
[68] DeBolt S. Copy number variation shapes genome diversity in Arabidopsis over immediate family generational scales. Genome Biology and Evolution. 2010;2:441-53. doi:10.1093/gbe/evq033.
[69] Gresham D, Desai MM, Tucker CM, Jenq HT, Pai DA, Ward A, et al. The repertoire and dynamics of evolutionary adaptations to controlled nutrient-limited environments in yeast. PLoS Genet. 2008;4 12:e1000303. doi:10.1371/journal.pgen.1000303.
[70] Farslow JC, Lipinski KJ, Packard LB, Edgley ML, Taylor J, Flibotte S, et al. Rapid Increase in frequency of gene copy-number variants during experimental evolution in Caenorhabditis elegans. BMC Genomics. 2015;16:1044. doi:10.1186/s12864-015-2253-2.
[71] Axelsson E, Ratnakumar A, Arendt M-L, Maqbool K, Webster MT, Perloski M, et al. The genomic signature of dog domestication reveals adaptation to a starch-rich diet. Nature. 2013;495 7441:360-4. doi:10.1038/nature11837.
[72] Reiter T, Jagoda E and Capellini TD. Dietary Variation and Evolution of Gene Copy Number among Dog Breeds. PLoS One. 2016;11 2:e0148899. doi:10.1371/journal.pone.0148899.

4) As the two sites: [candidate genes] UTP25 and FBRSL1 showed significant changes/linkages to progressing selection for breast muscle yield, across generations, a sound discussion on their roles in ducks in terms of meat yield, meat quality, and breast meat composition etc… can be welcome!
Response: Thanks for your comments. We have adapted your advice and added some discussion about the potential functions of UTP25 and FBRSL1 in muscles in line 239-248 of the revision.
The new section in the Discussion is reproduced here: "In this study, we identified two genes UTP25 and FBRSL1 significantly associated with breast muscle weight. FBRSL1 belongs to the Polycomb group (PcG) gene and is essential for many biological processes in mammals, including stem cell maintenance and differentiation [53, 54]. Our results showed that the expression level of FBRSL1 in the breast muscle of Pekin duck Z2 line decreased with the increase of age. This may hint at FBRSL1 was strongly selected to promote the development of Pekin duck breast muscle. UTP25 (also named DIEXF in human) reportedly affects digestive organ expansion by regulating p53 pathway [55, 56]. However, the function of UTP25 has not been characterized in animal muscles. UTP25 was strongly selected in the continuous breeding process of Pekin duck Z2 line, and further investigation would be necessary to elucidate its functions."
[53] Bathla S, Rawat P, Baithalu R, Yadav ML, Naru J, Tiwari A, et al. Profiling of urinary proteins in Karan Fries cows reveals more than 1550 proteins. J Proteomics. 2015;127 Pt A:193-201. doi:10.1016/j.jprot.2015.05.026.

[54] Chen Z, Yao Y, Ma P, Wang Q and Pan Y. Haplotype-based genome-wide association study identifies loci and candidate genes for milk yield in Holsteins. PLoS One. 2018;13 2:e0192695. doi:10.1371/journal.pone.0192695.
[55] Zhang S-J, Liu C-J, Yu P, Zhong X, Chen J-Y, Yang X, et al. Evolutionary Interrogation of Human Biology in Well-Annotated Genomic Framework of Rhesus Macaque. Molecular Biology and Evolution. 2014;31 5:1309-24. doi:10.1093/molbev/msu084.
[56] Chen J, Ruan H, Ng SM, Gao C, Soo HM, Wu W, et al. Loss of function of def selectively up-regulates Delta113p53 expression to arrest expansion growth of digestive organs in zebrafish. Genes & Development. 2005;19 23:2900-11.

Close
