# Supplementary material for: Resequencing of a Pekin duck breeding population provides insights into the genomic response to short-term artificial selection
Source: Gigascience. 2023 Mar 27;12:giad016. doi: 10.1093/gigascience/giad016 (PMC10041536; doi:10.1093/gigascience/giad016)
Supplement: giad016_Reviewer_1_Report_Original_Submission — Gene Ng -- 11/15/2022 Reviewed [file giad016_reviewer_1_report_original_submission.pdf]

Reviewer Report

Title: Resequencing of a Pekin duck breeding population provides insights into the genomic response to short-term artificial selection

Version: Original Submission **Date:** 11/15/2022

Reviewer name: Gene Ng

Reviewer Comments to Author:

The authors assembled a new genome for Pekin duck and resequenced highly selected populations, a few candidate genes for breast muscle weight improvement were found. There are many major shortcomings that prevent me from recommending the publication of the current draft.
Comments:
1) In lines 35-28, The authors stated: "However, the genetic basis of short-term, intense artificial selection remains poorly understood. Although many studies have elucidated genetic mechanisms involved in intense artificial selection[4-8], most of them have not been genome-scale investigations." This might be true ten years ago, but there're many genomic-scale studies nowadays. Please cite important review papers instead and revise this paragraph.
2) In lines 59-65, those sentences were repeated almost exactly in the Data Description section. Please rephrase.
3) In line 84, "to assist in splicing the PacBio contigs into scaffolds", "splicing" should be the wrong word.
4) In lines 85-87, please explain why the scaffold numbers were greater after clustering by the Hi-C data than those using optical mapping while the scaffold N50 was significantly improved but contig N50 decreased.
5) In lines 104-106, the authors stated: "we conducted the resequencing of 30 individuals (15 males, 15 females) from G1 to G10 in intervals of 3 generations.", I wonder how the total number could add up to 119.
6) In lines 189-190, the authors stated: "In total, we identified 2 duplicated CNVRs and 1 deletion between the G1 and G10 populations (Fig. 5A and B)." This can be misinterpreted as these CNVRs only occurred in the G10 population but not in the G1 population. Please rephrase.
7) In line 201, I doubt that "intelligence" should be applied to animals in this case, and I found that the interpretation in lines 202-205 was pretty unconvincing, especially while EXOC4 is also highly expressed in mammalian skeletal muscle. TRPA1 is also involved in skeletal muscle repair in mammals. However, the functions of these genes in muscle were not properly considered in the manuscript.
8ï¼‰Potential functions of UTP25 and FBRSL1 in muscles were not considered and discussed.
9ï¼‰ Gene annotation methods were not described in the Methods section.
10ï¼‰ What are the three panels in Fig. 5B?

Methods

Are the methods appropriate to the aims of the study, are they well described, and are necessary controls included? Choose an item.

Conclusions

Are the conclusions adequately supported by the data shown? Choose an item.

Reporting Standards

Does the manuscript adhere to the journal's guidelines on minimum standards of reporting? Choose an item.

Choose an item.

Statistics

Are you able to assess all statistics in the manuscript, including the appropriateness of statistical tests used? Choose an item.

Quality of Written English

Please indicate the quality of language in the manuscript: Choose an item.

Declaration of Competing Interests

Please complete a declaration of competing interests, considering the following questions:

- Have you in the past five years received reimbursements, fees, funding, or salary from an organisation that may in any way gain or lose financially from the publication of this manuscript, either now or in the future?
- Do you hold any stocks or shares in an organisation that may in any way gain or lose financially from the publication of this manuscript, either now or in the future?
- Do you hold or are you currently applying for any patents relating to the content of the manuscript?
- Have you received reimbursements, fees, funding, or salary from an organization that holds or has applied for patents relating to the content of the manuscript?
- Do you have any other financial competing interests?
- Do you have any non-financial competing interests in relation to this paper?

If you can answer no to all of the above, write 'I declare that I have no competing interests' below. If your reply is yes to any, please give details below.

I declare that I have no competing interests

I agree to the open peer review policy of the journal. I understand that my name will be included on my report to the authors and, if the manuscript is accepted for publication, my named report including any attachments I upload will be posted on the website along with the authors' responses. I agree for my

report to be made available under an Open Access Creative Commons CC-BY license (http://creativecommons.org/licenses/by/4.0/). I understand that any comments which I do not wish to be included in my named report can be included as confidential comments to the editors, which will not be published.

Choose an item.

To further support our reviewers, we have joined with Publons, where you can gain additional credit to further highlight your hard work (see: https://publons.com/journal/530/gigascience). On publication of this paper, your review will be automatically added to Publons, you can then choose whether or not to claim your Publons credit. I understand this statement.

Yes Choose an item.
