# Supplementary material for: Resequencing of a Pekin duck breeding population provides insights into the genomic response to short-term artificial selection
Source: Gigascience. 2023 Mar 27;12:giad016. doi: 10.1093/gigascience/giad016 (PMC10041536; doi:10.1093/gigascience/giad016)
Supplement: giad016_Reviewer_2_Report_Original_Submission — Surya kanta Mishra -- 12/11/2022 Reviewed [file giad016_reviewer_2_report_original_submission.pdf]

Reviewer Report

Title: Resequencing of a Pekin duck breeding population provides insights into the genomic response to short-term artificial selection

Version: Original Submission **Date:** 12/11/2022

Reviewer name: Surya kanta Mishra

Reviewer Comments to Author:

The Manuscript titled "Resequencing of a Pekin duck breeding population provides insights into the genomic response to short-term artificial selection" presented an interesting and thought-provoking piece of research work aimed to delineate some visible molecular-changes, concomitant to ongoing shorter-term high-intensity genetic selection for Breast-yield etc., in a Meat type duck line (Pekin ducks). Obviously, the planning and designing of the experiment with intermittent DNA sampling of the said population, for ultimate Re-sequencing of the individual duck DNA samples, called for a meticulous endeavour, which has been detailed in the project. It is heartening that the Authors [Simeng Yu et al.] have recorded significant GWAS based evidence that, 2 regions of the Pekin genome spanning 0.36 Mb, containing UTP25 and FBRSL1 were most likely to contribute to breast-muscle weight improvement. Further, a copy number variation region containing entire EXOC4 gene was identified, which could explain 1.9% of the variance in breast muscle weight!
The authors have included numerous graphs, images and tables to add credence to the MS. Accordingly, the reviewer opines that such an article deserves an appreciation, as being a lead article in duck genomics, documenting SNP -frequency changes) as a sequelae to Genetic selection.
While this MS moves further, the reviewer suggests the following to be included in the MS to make it explicit for certain facts, for appreciation of the Reading community.
1) While the Breast muscle yield of the Pekin line Z2 significantly changed from 80g to 220g over 10 generation of selection, details of this line with respect to its prime trait, 6Week's live weight, feed efficiency and other breast yield as a proportion of total meat-yield should be provided in the MS. It is obvious to detail that when breast yield improved more than 2 fold over the 10 generation's period, how good were the other meaty-cuts with reference to breast muscle yield. The Breeding process of Z2 (page 7) needs appropriate elaboration too.
2) While most narration on Re-sequencing of the generation specific DNA samples are relevant, making a lot of descriptions by including many digital large images appear redundant, and therefore, inclusion of too many images to drive home the points need to be looked at thoroughly. Why need more than 2 or 3 images, when the facts can be well-cited as texts!
3) For a study of such elaborate dimension, the discussion appears little too condensed, and therefore, be optimized to discuss, how the last 10 generations of selection brought so much of perceptible changes at molecular level, which were concerning more to non-coding regions than coding sequences. Discussions also may be beefed up citing experience of other genome studies (even beyond chickens and turkeys, to other food-purpose livestocks), for why CNV regions could be more important hotspots to reveal selection-induced molecular changes in Pekin ducks.

4) As the two sites: [candidate genes] UTP25 and FBRSL1 showed significant changes/linkages to progressing selection for breast muscle yield, across generations, a sound discussion on their roles in ducks in terms of meat yield, meat quality, and breast meat composition etc… can be welcome!

Methods

Are the methods appropriate to the aims of the study, are they well described, and are necessary controls included? Choose an item.

Conclusions

Are the conclusions adequately supported by the data shown? Choose an item.

Reporting Standards

Does the manuscript adhere to the journal's guidelines on [minimum standards of reporting?]() Choose an item.

Choose an item.

Statistics

Are you able to assess all statistics in the manuscript, including the appropriateness of statistical tests used? Choose an item.

Quality of Written English

Please indicate the quality of language in the manuscript: Choose an item.

Declaration of Competing Interests

Please complete a declaration of competing interests, considering the following questions:

- Have you in the past five years received reimbursements, fees, funding, or salary from an organisation that may in any way gain or lose financially from the publication of this manuscript, either now or in the future?
- Do you hold any stocks or shares in an organisation that may in any way gain or lose financially from the publication of this manuscript, either now or in the future?
- Do you hold or are you currently applying for any patents relating to the content of the manuscript?
- Have you received reimbursements, fees, funding, or salary from an organization that holds or has applied for patents relating to the content of the manuscript?
- Do you have any other financial competing interests?
- Do you have any non-financial competing interests in relation to this paper?

If you can answer no to all of the above, write 'I declare that I have no competing interests' below. If your reply is yes to any, please give details below.

No to all of above

I agree to the open peer review policy of the journal. I understand that my name will be included on my report to the authors and, if the manuscript is accepted for publication, my named report including any attachments I upload will be posted on the website along with the authors' responses. I agree for my report to be made available under an Open Access Creative Commons CC-BY license (http://creativecommons.org/licenses/by/4.0/). I understand that any comments which I do not wish to be included in my named report can be included as confidential comments to the editors, which will not be published.

Choose an item.

To further support our reviewers, we have joined with Publons, where you can gain additional credit to further highlight your hard work (see: https://publons.com/journal/530/gigascience). On publication of this paper, your review will be automatically added to Publons, you can then choose whether or not to claim your Publons credit. I understand this statement.

Yes Choose an item.
